# Analysis of a Functional Dimer Model of Ubiquinol Cytochrome *c* Oxidoreductase

**DOI:** 10.1016/j.bpj.2017.08.018

**Published:** 2017-10-03

**Authors:** Jason N. Bazil

**Affiliations:** 1 Department of Physiology, Michigan State University, East Lansing, Michigan

## Abstract

Ubiquinol cytochrome *c* oxidoreductase (*bc*_1_ complex) serves as an important electron junction in many respiratory systems. It funnels electrons coming from NADH and ubiquinol to cytochrome *c*, but it is also capable of producing significant amounts of the free radical superoxide. In situ and in other experimental systems, the enzyme exists as a dimer. But until recently, it was believed to operate as a functional monomer. Here we show that a functional dimer model is capable of explaining both kinetic and superoxide production rate data. The model consists of six electronic states characterized by the number of electrons deposited on the complex. It is fully reversible and strictly adheres to the thermodynamics governing the reactions. A total of nine independent data sets were used to parameterize the model. To explain the data with a consistent set of parameters, it was necessary to incorporate intramonomer Coulombic effects between hemes *b*_L_ and *b*_H_ and intermonomer Coulombic effects between *b*_L_ hemes. The fitted repulsion energies fall within the theoretical range of electrostatic calculations. In addition, model analysis demonstrates that the *Q* pool is mostly oxidized under normal physiological operation but can switch to a more reduced state when reverse electron transport conditions are in place.

## Introduction

Ubiquinol cytochrome *c* oxidoreductase (*bc*_1_ complex) is an essential enzyme for all mammalian cells. This enzyme family catalyzes a proton-coupled redox reaction that accounts for a significant fraction of energy required to maintain the proton motive force across biological membranes (1). The proton motive force is converted to a chemical potential energy in the form of the ATP hydrolysis potential (2), which is used to drive nearly all cellular processes. The enzyme serves as a hub for biological electron flow where many upstream catabolic pathways converge on the ubiquinone or quinone (Q) pool (3, 4). Quinones are lipophilic, mobile electron carrier analogs to the hydrophilic NADH molecule and cytochrome *c* hemeprotein. The complex catalyzes the exergonic two-electron oxidation of quinol to the one-electron reduction of cytochrome *c* while transferring two charge-equivalents across biological membranes. The net biochemical reaction is the oxidation of a quinol molecule, reduction of two cytochrome *c* hemeproteins, release of four chemical protons on the opposite side of charge transfer, and consumption of two matrix protons. The net biochemical reaction is depicted as

(1) $$QH_{2}+2c^{3+}+2H_{n}^{+}⇌Q+2c^{2+}+4H_{p}^{+}.$$

The enzyme operates using a Q-cycle mechanism whereby electron flow is bifurcated. Quinol is oxidized at the Q_p_ site, located on positive (or outer/intermembrane space) side of the membrane. The first electron is passed down the high potential chain to reduce cytochrome *c* at this outer surface. The other is sent down the low potential chain consisting of *b*-type hemes to reduce a quinone to form a stable semiquinone at the Q_n_ site, located on the negative (or inner/matrix) side of the membrane. Another turnover (i.e., a second quinol oxidation at the Q_p_ site) generates a second reduced cytochrome *c* molecule and a fully reduced quinol at the Q_n_ site (matrix side). In total, two quinol molecules are oxidized to form two reduced cytochrome *c* molecules and regenerate a quinol molecule at the Q_n_ site.

In mitochondria, the *bc*_1_ complex plays an important role in regulating energy transduction and free radical generation, but the precise mechanisms are still relatively obscure. Crystal structure data of the mammalian complex has helped to resolved many questions centered on the reaction mechanism. The first structures published showed the enzyme crystalized as a dimer and localized key catalytic components on the complex (5, 6, 7). Additional structures demonstrated that mobility of the iron sulfur protein (ISP) head domain was essential for catalytic function (8) and revealed some of the Q_n_-site details (9, 10). Structures from other organisms also helped shape the catalytic landscape (11, 12, 13). More details covering structural analysis of the *bc*_1_ complex and the Q-cycle mechanism are covered elsewhere (14, 15).

Although the Q-cycle mechanism is generally well accepted (16), the details of all of the biochemical reactions occurring on the *bc*_1_ complex are not. For example, the existence of a semiquinone during quinol oxidation at the Q_p_ site has been disputed for many years. Some groups postulated a mechanism involving a concerted two-electron oxidation (17, 18, 19). Other groups have provided evidence for the existence of a fleeting semiquinone serving as a reaction intermediary during the catalytic reactions of quinol between the Rieske ISP and heme *b*_L_ (20, 21). In favor of this hypothesis, Cape et al. (22) and Vennam et al. (23) have shown that this semiquinone does indeed exist, albeit only in conditions that maximize the likelihood of finding it. Other studies have also reported finding a semiquinone intermediary at the Q_p_ site (24, 25), but as pointed out by Pietras et al. (26), this matter is far from being resolved. In addition, the reaction at the Q_n_-site has also been the subject of some controversy. Mulkidjanian (27) argues in favor of an activated Q-cycle mechanism whereby quinol oxidation at the Q_n_ site primes the enzyme for catalysis at the Q_p_ site. The mode of free radical production is also not settled. Some experiments clearly show an increase in the rate of free radical production with membrane potential (28, 29), whereas others show a decrease in the presence of a membrane potential (30). Furthermore, the ability of the *bc*_1_ complex to operate as a functional dimer has been a point of contention. Three groups have produced compelling evidence to suggest intermonomer electron transfer occurs on a sufficiently rapid timescale to support the dimer mechanism (18, 31, 32, 33, 34, 35), whereas another calls into question the interpretation of those experiments (36, 37). However, there is now convincing evidence of dimeric function in vivo (38), although the effect of dimeric function on enzyme kinetics and free radical generation has not been fully explored.

Although other mathematical models of the *bc*_1_ complex exist (30, 39, 40, 41, 42, 43, 44, 45, 46, 47, 48, 49, 50, 51), they are too complex to integrate into larger-scale metabolic models (47, 48, 49), do not include free radical production (39, 40, 41, 42, 43, 44, 45, 46, 48), are not thermodynamically constrained (30, 42, 43, 44, 45, 46, 50, 51), or simulate a limited range of conditions (30, 41, 42, 43, 44, 45, 46, 48). A steady-state model capable of simulating both catalytic activity and important side reactions is an ideal choice for integration into next-generation mitochondrial bioenergetics models. Here we present a steady-state model of the kinetics of the *bc*_1_ complex capable of simultaneously simulating the rate of free radical production over a range of conditions. In addition, the method employed is generalized from our prior models (52, 53) and easily adaptable to other enzyme-mediated processes.

The model is used to test whether or not the Q cycle is sufficient to explain the available data, the mechanism of free radical production, and the feasibility of dimer operation. The model assumes that electron input at the Q_p_ and Q_n_ sites is independent, and that electrons are free to distribute themselves across either monomer to settle in the lowest energy state. We previously showed that a simple monomer model was sufficient to explain a majority of the kinetic data (53). However, simulating free radical production in addition to the available kinetic data requires a dimer model.

## Methods

### Generalized constitutive model equations

**Figure 1:**
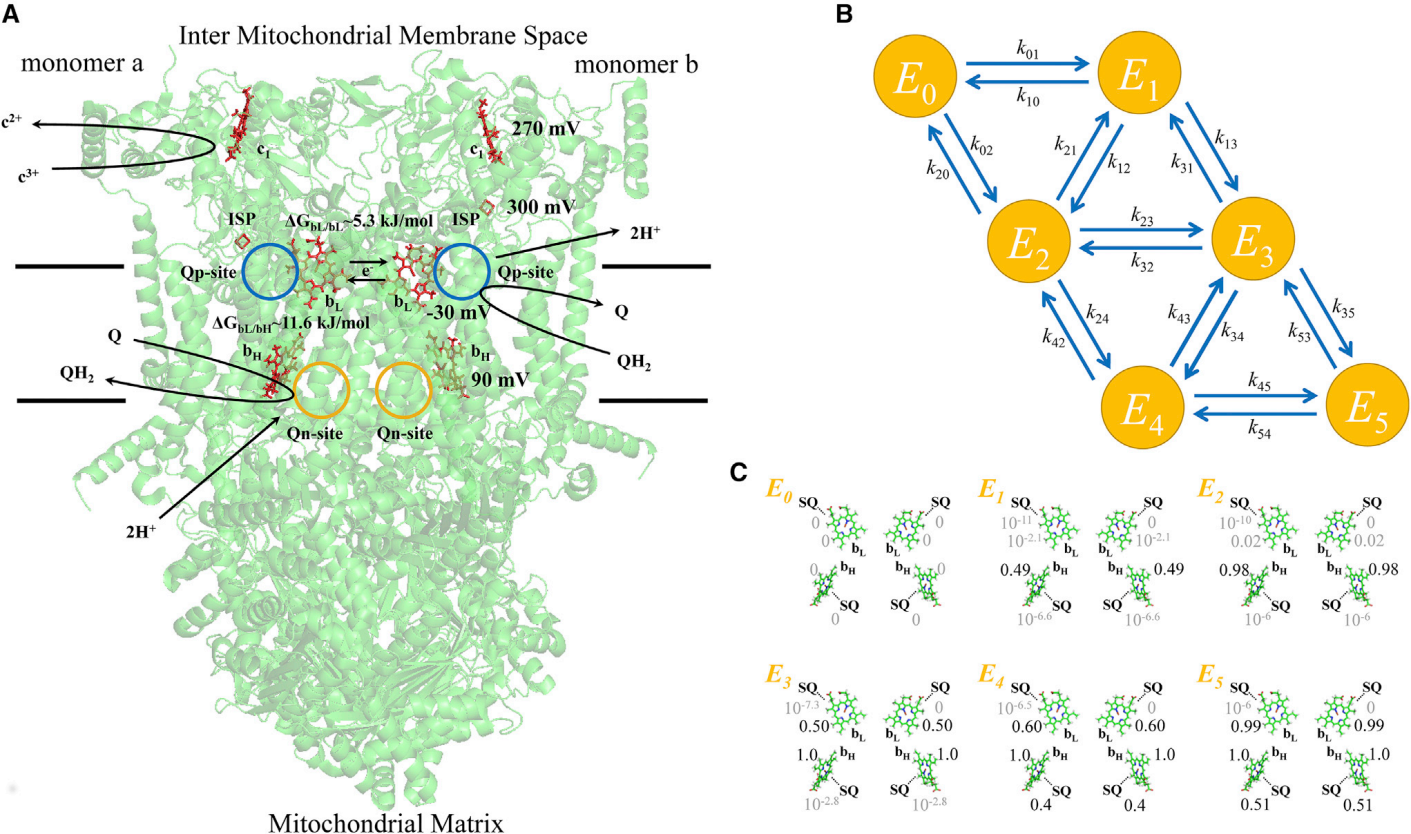
Model diagram of the *bc*_1_ dimer. (*A*) Dimeric model of the *bc*_1_ complex is shown with major redox centers and partial reactions. Midpoint potentials for the redox centers on the complex are from Table S1 and represented at pH 7. The model assumes only one Q_p_ site is active per turnover (31). Two turnovers at the Q_p_ site are required per turnover at the Q_n_ site. The model lumps oxidation of QH_2_ at the Q_p_ site into a two-electron step. The first electron is used to reduce cytochrome *c*, and the second electron is deposited on the *bc*_1_ complex. The monomer order during QH_2_ oxidation at the Q_p_ site is random. Quinone reduction at the Q_n_ site is also random. Electrons on the complex distribute themselves among the redox centers according to the Boltzmann distribution. Electron transport between *b* hemes and the Q_n_ site is electrogenic. Coulombic interaction energies between intramonomer *b*_L_ and *b*_H_ hemes and intermonomer *b*_L_ and *b*_L_ hemes are included. Blue and yellow circles are approximate locations of Q_p_- and Q_n_-site binding pockets, respectively. The dimer cartoon was generated from the crystal structure by Esser et al. (13) (PBD: 5KLV). The depicted proton uptake and release pathways are only for visual purposes. (*B*) State representation of the model is shown where *E*_*i*_ is the *i*th electronic state corresponding to the number of electrons residing on the complex. The state-transition rate constants, *k*_*ij*_, are given in the Supporting Material. Probabilities <0.4 are shown in gray. (*C*) For each enzyme state, *E*_*i*_, the probability of finding an electron on each redox center in the model is shown for the following conditions: Q pool 10% reduced, membrane potential of 0 mV, pH 7 on both sides of the membrane, no cytochrome *c* present, and under anoxia. Cartoon rendering of the *bc*_1_ complex was done using the software PyMOL (108). To see this figure in color, go online.

The model presented herein is more biophysically consistent with the known mechanism and crystal structure than our previous model of the *bc*_1_ complex (53), and the modeling approach is more similar to our Complex I model (52). An overall model scheme is presented in Fig. 1. The model is constructed using mass action kinetics and assumes rapid binding and unbinding of substrates and products. The model equations strictly adhere to the thermodynamics governing the reaction at each elementary step in the catalytic cycle. The oxidation and reduction reactions occur when the enzyme is in the appropriate enzyme-substrate complex. The enzyme-substrate complexes are computed using binding polynomials. Briefly, electrons are added to the complex via ubiquinol (QH_2_) oxidation in a two-electron step (32) at the Q_p_ site and removed via Q reduction at the Q_n_ site and cytochrome *c* reduction at the cytochrome *c*_1_ site. For simplicity, we lump QH_2_ oxidation at the Q_p_ site with cytochrome *c* reduction at the *c*_1_ site, so electrons enter the *bc*_1_ complex one at a time at the Q_p_ site. Electrons can also be removed via a secondary mechanism by molecular oxygen at the Q_p_ site to form superoxide:

(2) $$QH_{2}+c^{3+}+O_{2}\overset{\text{at}\;Q_{p}\;\text{site}}{⇌}Q+c^{2+}+O_{2}^{.-}+2H_{P}^{+}.$$

In addition, the mechanism is fully reversible. So, for example, in reverse electron transport, electrons can be added via QH_2_ oxidation at the Q_n_ site and removed by Q reduction at the Q_p_ site. The model is based on a functional dimer mechanism (31) with each monomer containing four redox centers. We assume the Rieske ISP and cytochrome *c*_1_ redox kinetics are not rate limiting under normal turnover conditions. This assumption is supported by experimental evidence showing QH_2_ oxidation at the Q_p_ site is the rate-limiting step (21). We also include the effect of pH on electron transport down the high potential chain. Accordingly, the four redox centers per monomer that influence the overall reaction rate are a semiquinone at the Q_p_ site, a heme b_L_, a heme *b*_H_, and a semiquinone at the Q_n_ site. In the dimer, the electrons equilibrate on the complex through the heme *b*_L_-*b*_L_ electronic bus bar (i.e., electron bridge) (35). In addition, electron mobility is restricted by Coulombic repulsion interactions (54). With eight redox centers in the dimer model, the theoretical maximum number of electrons on the complex is eight. However, model simulations reveal an extremely low occupancy of states past five electrons. In addition, the minimum number of states necessary to simulate the antimycin A-inhibited complex is six. Therefore, only six electronic states are considered (oxidized plus up to the five-electron state).

The fractional substates of these redox centers for each electronic state are computed using the Boltzmann distribution:

(3) $$s_{r}^{k}=\frac{e^{-\Delta G_{r}^{k}/RT}}{\sum_{r}e^{-\Delta G_{r}^{k}/RT}},$$

where *s*^*k*^_*r*_ is the fraction of that redox center(s) *r* existing in electronic state *k* that is reduced, and Δ*G*_*r*_^*k*^ is the standard free energy change for center(s) *r*. An example of the substate fractions given by the model under specific conditions is given in Fig. 1 *C*. For details of the conditions, see the figure legend. In Eq. 3, the redox center(s) *r* can consist of a single redox center or any combination of centers on the dimer. To compute the standard free energy change for combinations of redox centers, we assume additivity of the individual redox center standard free energies. The number of combinations of reduced redox centers for each state is given by the binomial coefficient where *n* is the number of redox centers and *k* is the number of electrons on the complex. The free energy change calculations for each redox center used in the model are given in Eqs. S14–S24 in the Supporting Material. Coulombic electrostatic energies are included in the dimer for the *b*_H_/*b*_L_ monomeric and *b*_L_/*b*_L_ dimeric interactions.

The remaining constitutive model equations are given in the Supporting Material, but a brief description of the model follows. To compute the steady-state turnover of the *bc*_1_ complex for a given set of conditions (substrate and product concentrations, pH, membrane potential, etc.), we first need to solve the linear system of equations describing the steady-state relationship between the electronic states (*E*_*i*_) considered in the model (see Eq. S25 in the Supporting Material). These equations are graphically depicted in Fig. 1 *B*. The edges connecting the electronic states represent partial reactions that govern how state *i* is connected to state *j*. These partial reactions encompass molecular processes such as QH_2_ oxidation at the Q_p_ site, Q reduction at the Q_n_ site, and superoxide formation at the Q_p_ site. The equations for these partial reactions are given in Eqs. S26–S43 in the Supporting Material. When state transitions occur (e.g., *E*_*i*_ → *E*_*j*_), the enzyme needs to be in the appropriate enzyme-substrate complex and substate form. For example, before Q_p_-site catalysis can occur, the enzyme needs to be bound with QH_2_ and ferricytochrome *c* (*c*^3+^). For QH_2_ to bind to the complex, the Q_p_ site needs to be unoccupied. Likewise, the *c*_1_ site needs to be unoccupied before *c*^3+^ can bind. (Recall that we lump QH_2_ oxidation and *c*^3+^ reduction into a single step for simplicity.) As another example, before QH_2_ can form and dissociate from the complex at the Q_n_ site, the enzyme needs to be in the appropriate substate form. This requires a semiquinone (SQ) bound at the Q_n_ site with a reduced *b*_H_ heme on the same monomer. The fraction during which a given electronic state exists for this substate is computed using Eq. 3. With a steady-state solution for the electronic states known, the steady-state turnover rates for cytochrome *c* reduction, superoxide production, QH_2_ oxidation, and Q reduction can be computed using Eqs. S44–S54 in the Supporting Material. For more details, we refer the reader to the Supporting Material.

### Experimental data

To calibrate the model, we used a wide variety of data from the literature. These data consist of kinetic data (41, 43, 44, 55, 56) in addition to data on superoxide production rates (28, 57) and dimeric function (31). The superoxide data consist of rich data sets that report the rate of superoxide production as a function of membrane potential and the effect of antimycin A on both cytochrome *c* reduction and superoxide production. We also included additional data to constrain the maximum rate of antimycin A-stimulated free radical production (58) in mammalian mitochondria. The monomer model cannot simultaneously fit these data with a single parameter set; thus, a new dimer model is necessary to explain the superoxide production and antimycin A-stimulation data. For more details about the data sets used for parameter estimation, see Table S2.

In many experiments used to parameterize the model, the Q analogs exhibited a nonenzymatic reaction with cytochrome *c* that is strongly pH dependent (59, 60). In most articles, the authors indicated that this oxidation rate was subtracted from the measured rate of cytochrome *c* reduction. Unfortunately, the rates were not reported in many of the studies, nor did the experimental methods give enough details to allow for this phenomenon to be accurately modeled. Therefore, we had to estimate the extent of oxidation for each data set with an adjustable parameter (see Table 1). This parameter is a measure of how much of the reduced Q analog was oxidized before the rate of the enzymatic reaction was recorded.

**Table 1:** Model Adjustable Parameters

Parameter	Definition	Value	Units	Sensitivity	Rank
Rate Constants					

$kf_{Q_{p}}^{0,1}$	quinol oxidation rate for *E*_0_	2.38E+03	s^−1^	1.57E−03	37
$kf_{Q_{p}}^{1,2}$	quinol oxidation rate for *E*_1_	1.26E+03	s^−1^	5.52E−01	4
$kf_{Q_{p}}^{2,3}$	quinol oxidation rate for *E*_2_	3.15E+03	s^−1^	2.33E−01	16
$kf_{Q_{p}}^{3,4}$	quinol oxidation rate for *E*_3_	2.27E+03	s^−1^	4.31E−02	26
$kf_{Q_{p}}^{4,5}$	quinol oxidation rate for *E*_4_	4.14E+00	s^−1^	3.50E−02	28
$\beta_{AA}^{r}$	antimycin A inhibition factor for semireverse mode of superoxide production	3.44E+02	—	3.68E−01	9
$\beta_{AA}^{f}$	antimycin A inhibition factor for semiforward mode of superoxide production	5.00E+00	—	3.82E−01	8
$kf_{Q_{n}}^{2,0}$	quinone reduction rate for *E*_2_	9.19E+09	s^−1^	4.43E−05	39
$kf_{Q_{n}}^{3,1}$	quinone reduction rate for *E*_3_	9.21E+09	s^−1^	2.23E−02	32
$kf_{Q_{n}}^{4,2}$	quinone reduction rate for *E*_4_	6.98E+03	s^−1^	1.05E−01	22
$kf_{Q_{n}}^{5,3}$	quinone reduction rate for *E*_5_	1.13E+04	s^−1^	1.92E−02	33

Binding Constants					

$K_{c^{3+}}$	*c*^3+^ binding constant	1.11E−06	M	2.61E−01	14
$K_{c^{2+}}$	*c*^2+^ binding constant	2.49E−06	M	9.17E−02	23
$K_{c^{3+}}^{{\text{Mg}}^{2+}}$	*c*^3+^ binding constant in presence of excess Mg^2+^	1.14E−05	M	4.74E−01	6
$K_{c^{2+}}^{{\text{Mg}}^{2+}}$	*c*^2+^ binding constant in presence of excess Mg^2+^	1.15E−05	M	2.38E−01	15
$K_{{\text{DQH}}_{2}}^{Q_{p}}$	DQH_2_ binding constant at Q_p_ site	2.76E−06	M	4.26E−01	7
$K_{\text{DQ}}^{Q_{p}}$	DQ binding constant at Q_p_ site	3.77E+00	M	5.95E−03	36
$K_{\text{DQ}}^{Q_{n}}$	DQ binding constant at Q_n_ site	1.00E+02	M	6.71E−06	41
$K_{{\text{DQH}}_{2}}^{Q_{n}}$	DQH_2_ binding constant at Q_n_ site	1.00E+00	M	1.50E−01	18
$K_{Q_{2}H_{2}}^{Q_{p}}$	Q_2_H_2_ binding constant at Q_p_ site	7.29E−06	M	3.49E−01	11
$K_{Q_{2}}^{Q_{p}}$	Q_2_ binding constant at Q_p_ site	1.57E+00	M	1.75E−02	34
$K_{Q_{2}}^{Q_{n}}$	Q_2_ binding constant at Q_n_ site	2.24E+01	M	1.38E−05	40
$K_{Q_{2}H_{2}}^{Q_{n}}$	Q_2_H_2_ binding constant at Q_n_ site	1.00E+00	M	7.17E−02	24
$K_{\text{NBH}}^{Q_{p}}$	NBH binding constant at Q_p_ site	1.23E−05	M	1.17E−01	20
$K_{\text{NB}}^{Q_{p}}$	NB binding constant at Q_p_ site	1.00E+00	M	4.16E−02	27
$K_{\text{NB}}^{Q_{n}}$	NB binding constant at Q_n_ site	3.41E+00	M	3.95E−04	38
$K_{\text{NBH}}^{Q_{n}}$	NBH binding constant at Q_n_ site	2.42E−01	M	2.59E−02	30
$K_{QH_{2}\text{mix}}^{Q_{p}}$	mixed Q_2_H_2_/Q_10_H_2_ binding constant at Q_p_ site	2.59E−08	M	8.63E−01	2
$K_{Q\text{mix}}^{Q_{p}}$	mixed Q_2_/Q_10_ binding constant at Q_p_ site	1.00E+00	M	4.88E−02	25
$K_{Q\;\text{mix}}^{Q_{n}}$	mixed Q_2_/Q_10_ binding constant at Q_n_ site	1.00E+00	M	2.13E−01	17
$K_{QH_{2}\;\text{mix}}^{Q_{n}}$	mixed Q_2_H_2_/Q_10_H_2_ binding constant at Q_n_ site	1.00E-02	M	8.62E−01	3

Thermodynamic					

$\Delta G_{b_{\text{H}}/b_{\text{L}}}^{\text{Coulomb}}$	*b*_H_/*b*_L_ monomeric Coulombic interaction energy	11.6	kJ/mol	1.36E+00	1
$\Delta G_{b_{\text{L}}/b_{\text{L}}}^{\text{Coulomb}}$	*b*_L_/*b*_L_ dimeric Coulombic interaction energy	5.3	kJ/mol	2.76E−01	13
$K_{S}^{Q10}$	*Q*_p_ site stability constant for Q_10_	2.28E−15	—	1.17E−01	19
$K_{S}^{Q\;\text{analog}}$	*Q*_p_ site stability constant for Q analogs	9.34E−09	—	3.18E−02	29

Initial Q-Pool Redox State (% Oxidized) for Data Sets					

Speck and Margoliash (44)	percent Q pool is oxidized	1.53	%	1.16E−01	21
Brandt and Okun (55)	percent Q pool is oxidized	5.01	%	2.37E−02	31
Esposti and Lenaz (41)	percent Q pool is oxidized	2.30	%	1.20E−02	35
Kubota et al. (43)	percent Q pool is oxidized	0.32	%	2.79E−01	12
Rottenberg et al. (28)	percent Q pool is oxidized	0.06	%	4.79E−01	5
Covian and Trumpower (78)	percent Q pool is oxidized	2.59	%	3.56E−01	10

Local sensitivity coefficients are normalized and averaged using Eqs. S55 and S56 in the Supporting Material. c^2+^, ferrocytochrome *c*; c^3+^, ferricytochrome c; DQH_2_, decylubiquinol; NBH, nonyl-ubihydroquinone; Q_2_H_2_, ubiquinol-2; Q_10_H_2_, ubiquonol-10.

### Model code

The model was developed, parameterized, and simulated on a Dell Precision T5810 workstation (Round Rock, TX) with an Intel Xeon CPU E5-2640 v3 (Santa Clara, CA) at 2.6 GHz and 32 GB RAM using the software MATLAB (v. 2016a; The MathWorks, Natick, MA). The steady-state equation for the six-state model was solved analytically using MATLAB’s symbolic toolbox. A custom, parallelized simulated annealing algorithm was used to globally search the parameter space before identifying a local minimum with a gradient-based local optimizer. Model code is given in the Supporting Material.

## Results and Discussion

### Fitted model parameters

The model adjustable parameters are listed in Table 1. The model parameters were identified by simultaneously fitting all the kinetic and superoxide data with a single consistent set of parameters. The dissociation constants for the various Q analogs are highly correlated and not reliably identifiable using only the kinetic data. Therefore, we opted to constrain these parameters by assuming the following: 1) the dissociation constants at the Q_p_ site are similar to each other and within an order of magnitude (61, 62); 2) the dissociation constant for QH_2_ at the Q_n_ site is equal to or up to two orders-of-magnitude smaller than the Q_p_ site dissociation constants (63); and 3) the dissociation constant for Q at the Q_p_ site is equal to or up to two orders-of-magnitude higher than the dissociation constant for QH_2_ at the Q_n_ site (63, 64). We emphasize that these dissociation constants are apparent dissociation constants in that they are taken with respect to the aqueous phase. To identify the actual dissociation constants, we would need to know the partition coefficients for all the Q analogs for the various organic phases used in the experiments. In addition, the rate constants for the reactions at the Q_p_- and Q_n_ sites were constrained by adding a difference penalty to the cost function; this difference penalty keeps the Q_p_- and Q_n_-site rate constants similar to each other during parameter estimation. But even with this penalty applied, a few rate constants were required to be significantly different from the others. Specifically, the Q_p_-site QH_2_ oxidation rate constant for the four-electron reduced state was low compared to the other rate constants. Also, the Q_n_-site Q-reduction rate constants for the four- and five-electron reduced states were lower than the other reductase rates constants. These differences were necessary to fit the data. For the former parameter, a low rate constant was required to prevent an unrealistic amount of superoxide production in the antimycin A-inhibited state. For the latter parameter, a lower rate constant was necessary to improve fits to the kinetic data sets collected under energized conditions.

The fits to the kinetic data used to calibrate the model are shown in Figs. S5–S9. These results show that the model is capable of recapitulating the observed cytochrome *c* reduction kinetics under a wide variety of experimental conditions. The effect of product inhibition, pH, and energization state are captured well by the model simulations. Unfortunately, these data were collected using hydrophilic Q analogs, so the dissociation constants obtained from model fitting are not able to be used to simulate *bc*_1_ kinetics under conditions with the native substrate, Q_10_. However, the rate constants fit by the model can be used with approximate Q_10_ dissociation constants to simulate *bc*_1_ kinetics and superoxide production rates in its native state. See the Native Q10 Dissociation Constants and Physiological Q-Pool Operating Range and Cardiac bc1 Content subsections below for details.

Sensitivity analysis identifies the top-10 ranked parameters that are associated with the internal energy states of the enzyme, the rate-limiting step in the catalytic cycle, the antimycin A-inhibition factors, four quinone and cytochrome *c* binding constants, and parameters related to the experimental design for two data sets. We should note that the sensitivity analysis is local and thus only reflects how changes in model parameters affect model outputs in the neighborhood of the optimal point in parameter space. In addition, the values given in Table 1 are averages of the nonzero local sensitivity coefficients computed from model outputs (cytochrome *c* reduction and superoxide production rates) coinciding with the data using Eqs. S55 and S56 in the Supporting Material, so they do not give a global perspective on how these parameters change the model outputs under experimental conditions not used for parameter estimation. The parameter correlation matrix heat map (Fig. S1) shows that the majority of parameters are relatively uncorrelated, with pockets of correlation centered on the specific Q-analog binding constants. In addition, some of the experimental design parameters are correlated with their respective Q-analog binding constants. Therefore, these binding constants are not readily identifiable without additional experimental data. The high sensitivity for some of the dissociation constants given in Table 1 reflects the importance of that parameter to fit that particular dataset. The fixed model parameters were obtained from the literature and are listed in Table S1.

### Superoxide production

**Figure 2:**
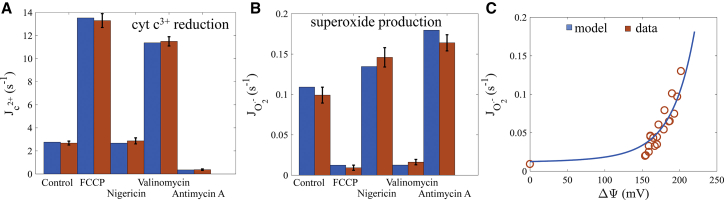
Cytochrome *c* turnover and superoxide production rate by the *bc*_1_ complex reconstituted in liposomes. (*A*) Cytochrome *c* reduction rates are given for a variety of pharmacological manipulations. In the control case, the membrane potential and (2.3 RT/F)ΔpH values were 200 and 15 mV, respectively. With FCCP, both the membrane potential and ΔpH were set to zero. In the nigericin case, the ΔpH was set to 0 mV and the membrane potential was set to 215 mV. When valinomycin was present, the membrane potential was set to 0 mV with the ΔpH unchanged from the control. And when antimycin was present, the Q_n_ sites were inhibited. (*B*) Superoxide formation rates for the same pharmacological manipulations for the cytochrome *c* reduction rate data are shown. (*C*) The model simulates an exponentially increasing rate of superoxide production as the membrane potential is increased. This is caused by electron retention at the Q_p_ site of the complex, which leads to higher semiquinone occupancy. Data are from Rottenberg et al. (28). To see this figure in color, go online.

The model simulations of the superoxide data from reconstituted *bc*_1_ complex (28) are shown in Fig. 2. It is important to note that these experiments were done under specific conditions that enabled robust superoxide production from the complex. The model reproduces the cytochrome *c* reduction (Fig. 2 *A*) and corresponding superoxide production rates (Fig. 2 *B*) for various pharmacological manipulations. Under the control conditions, the phospholipid vesicles reconstituted with *bc*_1_ complex exhibited moderate cytochrome *c* reduction rates with elevated superoxide production rates. This is due to the presence of a membrane potential and pH gradient across the membrane. With the addition of FCCP, both the membrane potential and pH gradient are abolished, and the rate of cytochrome *c* reduction significantly increases whereas the rate of superoxide production dramatically falls to the minimum level. When nigericin is present, the pH gradient is converted to a membrane potential that leads to more superoxide production with a negligible effect on cytochrome *c* reduction relative to the control. In the presence of valinomycin, the membrane potential is dissipated. This leads to a significant stimulation of cytochrome *c* reduction, and the superoxide production rate collapses to values similar to the FCCP condition. When antimycin A is added, superoxide production rates skyrocket to levels just above those seen when nigericin is present. The cytochrome *c* reduction rate falls to levels about twice that of the superoxide production. This is due to the bypass reaction whereby the first electron from QH_2_ reduces cytochrome *c*, the second electron reduces oxygen to produce superoxide (as shown in Eq. 2), and then the superoxide reduces cytochrome *c*. In addition, the model recapitulates the exponential dependency on membrane potential for superoxide production rather well (Fig. 2 *C*). This exponential relationship has been previously demonstrated by other groups (65, 66); however, the data set of Rottenberg et al. (28) is the only one to show superoxide production that is exclusively from the *bc*_1_ complex.

**Figure 3:**
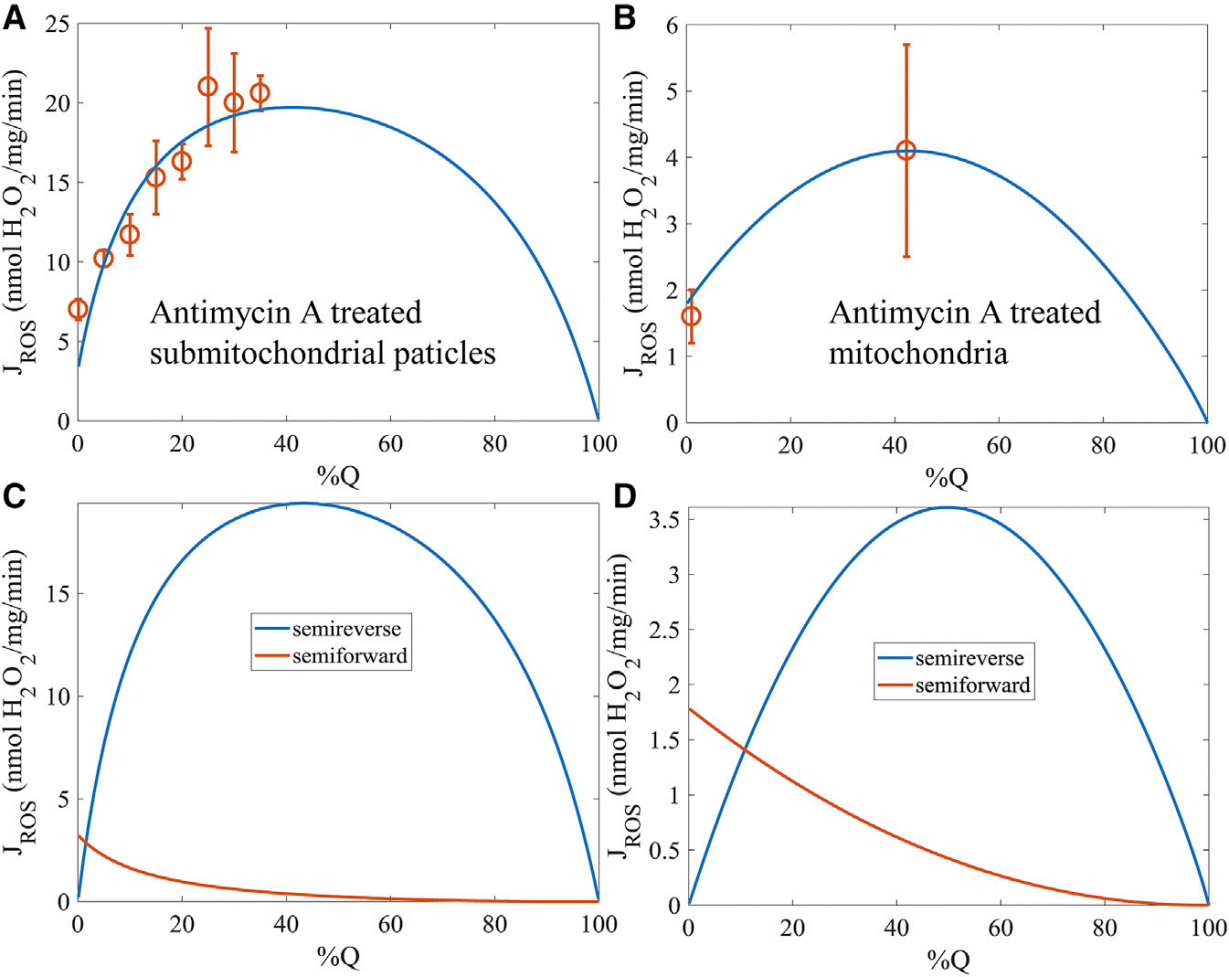
Free radical production rates by *bc*_1_ complex in submitochondrial particles and intact mitochondria. (*A*) Model simulations of superoxide production by antimycin A-treated submitochondrial particles as a function of the Q-pool redox state using DQ kinetic constants as given in Table 1. (*B*) Model simulations of superoxide production by antimycin A-treated mitochondria as a function of the redox state of the Q pool using Q_10_ kinetic constants as given in Table S1. (*C*) The semiforward and semireverse rates of superoxide production for the conditions given in (*A*). (*D*) The semiforward and semireverse rates of superoxide production for the conditions given in (*B*). To convert the superoxide production rate to H_2_O_2_ production, we assumed a stoichiometric relationship of two superoxide molecules per H_2_O_2_. Data in (*A*) are from Dröse and Brandt (57). Data in (*B*) are from Quinlan et al. (30). To see this figure in color, go online.

Model simulations of superoxide production data from antimycin A-inhibited submitochondrial particles (57) and intact skeletal muscle mitochondria inhibited by antimycin A (30) are shown in Fig. 3. The model simulates maximum levels of superoxide production when the Q pool is approximately half-oxidized (Fig. 3 *A*). These simulations were run with the decylubiquinone-related parameters (Table 1) and closely match data from a study by Dröse and Brandt (57). In this study, submitochondrial particles were fueled with a mixture of decylubiquinol/decylubiquinone, and the rate of free radical production was measured. They found the rate of superoxide production peaked when the Q pool was ∼30% oxidized, but the uncertainty in the data limits how precisely this value can be determined. The model simulates this phenomenon rather well, but it shows a peak closer to 40%. More data are necessary to accurately pinpoint the area where the peak of superoxide formation occurs. As shown in Fig. 3 *B*, the model simulates similar behavior when run with the native Q_10_ parameters (Table 1; Table S1). In this study, mitochondria were purified from skeletal muscle, and the *bc*_1_ complex was inhibited with antimycin A. The Q-pool redox state was modulated by the addition of succinate at various concentrations. The model predicts a peak free radical production of ∼4 nmol H_2_O_2_/mg/min (= 8 nmol superoxide/mg/min) when the Q pool is ∼45% oxidized (Fig. 3 *B*); when the Q pool is fully reduced, the rate is a little less than half of the maximum rate, near 1.5 nmol H_2_O_2_/mg/min. Unfortunately, in the study by Quinlan et al. (67), the Q-pool redox state is not known. This makes ascertaining the precise relationship between the Q-pool redox state and the superoxide production rate in this study impossible.

The superoxide production mechanism in the model includes both the semiforward and semireverse modes of superoxide production (26, 68, 69), as explained below. These results are given in Fig. 3, *C* and *D*. The dominant mechanism of superoxide production predicted by the model is the semireverse mode. This agrees well with the study by Sarewicz et al. (68). The semireverse mode of superoxide production occurs when the reduced heme *b*_L_ reduces Q at the Q_p_ site to form the unstable SQ that reacts with O_2_ (26). In the model, this mode of superoxide production occurs when superoxide is formed from states *E*_1_ through *E*_4_. This mode of superoxide production shows a bell-curve-like relationship with the Q-pool redox state (Fig. 3, *C* and *D*). When the Q pool is highly reduced, the availability of Q to react with a reduced heme *b*_L_ is limited. When the Q pool is highly oxidized, the number of electrons on the complex is low, and they predominantly reside on the *b*_H_ hemes. The semiforward mode of superoxide production occurs when QH_2_ is oxidized whereas heme *b*_L_ is reduced (26). This leaves an unstable SQ at the Q_p_ site that quickly reacts with oxygen to form superoxide. In the model, this mode of superoxide production occurs when superoxide is formed from state *E*_5_. In this state, all available redox centers are reduced, leaving no other option than the formation of an SQ after QH_2_ is oxidized at the Q_p_ site. This mode of superoxide production is highest when the Q pool is fully reduced. The rate monotonically decreases as the Q pool becomes more oxidized. This is due to a decrease in the state *E*_5_ as the fraction of QH_2_ declines.

To fit the superoxide production data for the antimycin A-inhibited complex, the QH_2_ oxidation rates were lowered when antimycin was bound to the Q_n_ site. This modification is supported by prior studies (30, 70, 71, 72, 73). (For model details, see Eqs. S57–S61 in the Supporting Material.) Without this modification, the maximum rate of superoxide production occurred when the Q pool was 80–90% oxidized (data not shown). The exact mechanism leading to the slowdown of QH_2_ oxidation at the Q_p_ site could be linked to the redox state of the *b* hemes (30, 70), mobility of the Riekse ISP (71, 72), and/or conformation changes in the protein structure (72, 73), but there is evidence disputing such long-range interactions (74). In the model, a minimum of two factors was required to explain the data (see Table 1). The first inhibition factor lowered the rate of QH_2_ oxidation for each state transition involved with the semireverse mode of superoxide production. A second inhibition factor was necessary for the semiforward mode of superoxide production.

Although this modification led to adequate fits to the available data, there are other possible mechanisms that can explain the data. Using the model, we can identify the critical processes that govern this phenomenon. The shape of the superoxide production rate profile as a function of the Q-pool redox state is determined by the net oxidation rates at the Q_p_ site. These net rates are determined by several conditions: 1) increased levels of reduced cytochrome *c*; 2) the Q and QH_2_ dissociation constants at the Q_p_ site; 3) superoxide scavenging by Q; and 4) antimycin A-dependent effects of the rate constants at the Q_p_ site, as discussed in the paragraph above. The model can be modified to include all these effects and still simulate the Q-stimulated superoxide production data from Dröse and Brandt (57). These factors are not mutually exclusive; however, we can rule out a few based on reasonable arguments and prior data. For condition 1, we found peak superoxide production rates near a 30% oxidized Q pool when cytochrome *c* was only 5% (57) or 20% (67) reduced. But it is unlikely that these levels of reduced cytochrome *c* are produced in either study. In the presence of antimycin A, cytochrome *c* oxidase keeps cytochrome *c* completely oxidized. Regarding condition 2, lowering the dissociation constant for Q by a factor of ∼10 at the Q_p_ site can also lead to good fits to the data; however, this is not supported by the available data (62, 75). For condition 3, superoxide scavenging by Q can explain the data (76). To include this mechanism, many additional parameters and assumptions are required to capture all the necessary details to properly model this phenomenon. Of all the potential mechanisms discussed, only condition 4 allowed for adequate fits to the data with the fewest additional parameters and changes to the model.

### Antimycin A stimulation of cytochrome *c* reduction

**Figure 4:**
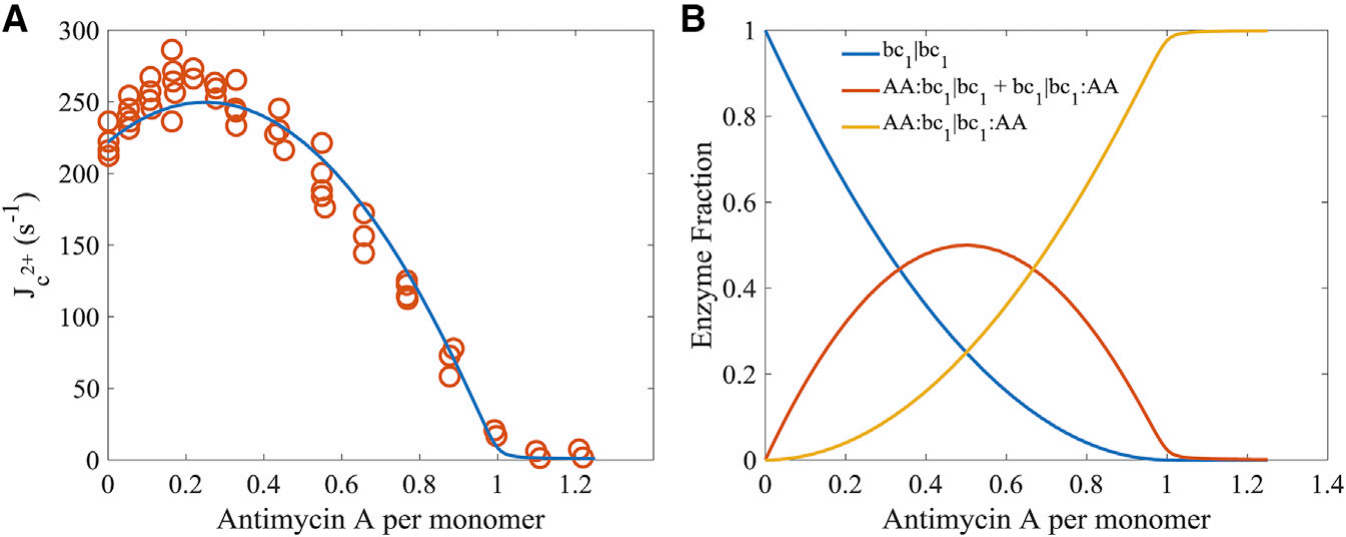
Antimycin-stimulated cytochrome *c* reduction. (*A*) At low antimycin A/monomer ratios (0–0.2), the rate of cytochrome *c* reduction is increased because both Q_p_ sites are active in the *bc*_1_ dimers bound with a single antimycin A molecule. (*B*) As antimycin A is titrated, the fraction of antimycin A bound per monomer increases and follows the binding curves shown. We assumed antimycin A binds independently to either monomer. Data are from Covian et al. (31). To see this figure in color, go online.

The ability of antimycin A to stimulate cytochrome *c* reduction by the *bc*_1_ complex is compelling evidence for cross-monomer electron equilibration (31). Mutagenic studies have led to this theory being nearly universally accepted (33, 38). In this specific case, antimycin A concentrations are extremely low, and a significant fraction of dimers are bound with only a single inhibitor molecule. At saturating concentrations, antimycin A binds tightly to all Q_n_ sites, and electron flux through the enzyme only occurs at the Q_p_ site, via bypass reactions forming superoxide (see Eq. 2) (77). However, when only a single antimycin A molecule is bound to the dimer, cytochrome *c* reduction is stimulated. Fig. 4 *A* shows the model simulations of this phenomenon. This is only possible if the enzyme operates as a functional dimer with electron equilibration between monomers. Covian et al. (31) suggest that their data reveal only one operational Q_p_ site per turnover when both Q_n_ sites of the dimer are either bound with antimycin A or not. But when a single antimycin A molecule is bound to the dimer at one of the Q_n_ sites, both Q_p_ sites become operational and the rate of cytochrome *c* reduction is increased by a factor of 2. Our model simulations corroborate this hypothesis; however, we find that this condition leads to a 1.5-fold increase of superoxide production by a single *bc*_1_ dimer. This leads to an overall increase in the maximal rate of cytochrome *c* reduction by a factor of 1.15 in the heterogeneously inhibited population of *bc*_1_ complexes. In their original analysis, Covian et al. (31) did not appear to assume the Q_n_ sites bind antimycin A independently as we have done here. Fig. 4 *B* shows the enzyme fraction without antimycin A bound (*blue*), with a single antimycin A molecule bound to the dimer (*orange*), and with two molecules of the inhibitor bound (*yellow*) as a function of the number of antimycin A molecules per monomer for the simulations shown in Fig. 4 *A*.

### Coulombic interactions, *bc*_1_ kinetics, and free radical generation

Coulombic interactions (electron repulsive forces) in the *bc*_1_ complex have been suggested to be important for controlling electron slippage (57), regulating enzyme turnover (54), and facilitating dimer operation (78). Of all the potential sites for Coulombic interactions in the *bc*_1_ complex, we found two major sites necessary to fit the data. These sites are located on the intramonomer low potential chain (*b*_H_ and *b*_L_) and the intermonomer dimeric interface (*b*_L_ and *b*_L_). Of these two sites, the intramonomer interaction was most important with a fitted interaction energy of 11.6 kJ/mol. This is equivalent to an electrostatic interaction of ∼−120 mV and well within the expected value assuming a protein dielectric constant in the range of 4–30 (21, 79) and an intramonomer distance of 20.7 Å between hemes *b*_L_ and *b*_H_ (80). For the intermonomer Coulombic interaction energy, a value of 5.3 kJ/mol was determined to best fit the data and corresponds to an electrostatic interaction of ∼−55 mV. Taking the intermonomer *b*_L_ distance as 20.9 Å (80) and same range of dielectric constants mentioned above, this value also falls into a theoretical span of possible interaction potentials. These Coulombic repulsive forces bias electron distribution to favor a reduced *b*_H_-SQ pair on one monomer relative to the condition in the absence of these repulsive forces. In this state, *bc*_1_ complex turnover is most efficient and not accompanied with significant superoxide production. In the absence of these repulsive forces, the probability of finding an SQ on the complex drops two and four orders of magnitude for states *E*_3_ and *E*_4_, respectively (data not shown). We found that limiting electron mobility on the complex using these Coulombic interactions was required to fit the data sets that included energized mitochondria (56), free radical production (28, 30, 57), and antimycin A (28, 30, 57) with the kinetic data (41, 43, 44, 55) using a single consistent parameter set.

### Native Q_10_ dissociation constants

**Figure 5:**
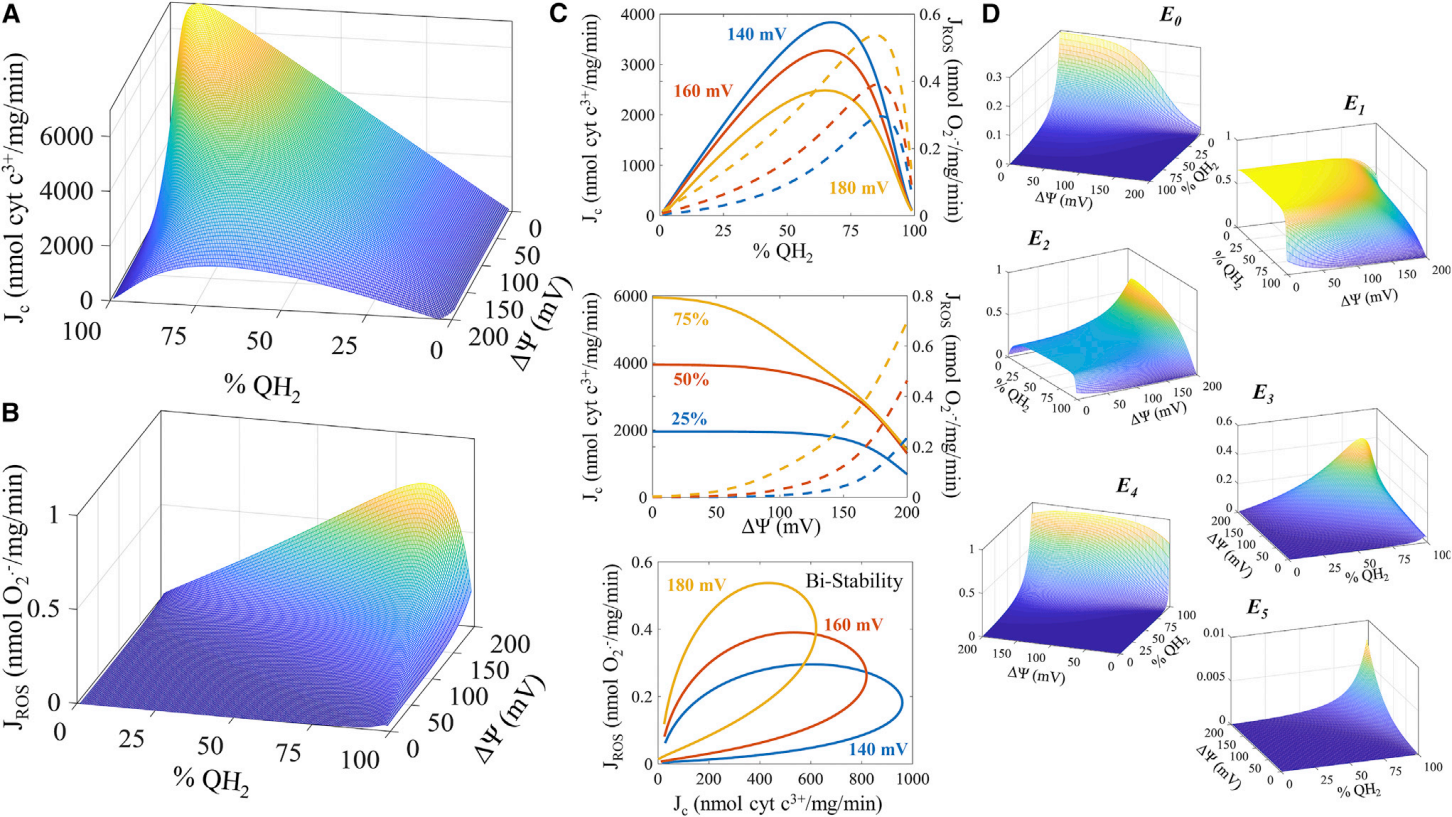
Physiological behavior of *bc*_1_ dimer. For all the simulations, the cytochrome *c* pool was 20% reduced, matrix and cytosolic pH was 7, superoxide concentration was 100 pM, and oxygen was 30 *μ*M. The Q pool was 20 mM and the reduced fraction and the membrane potential were variable as shown. (*A*) The cytochrome *c* reduction rates show a maximum rate when the Q pool is nearly fully reduced (∼90%) at 0 mV, which shifts to a maximum when the pool is ∼65% reduced at high membrane potentials. (*B*) The superoxide production simulations indicate a maximum when the Q pool is 75% reduced that shifts to ∼95% as the membrane potential approaches zero. (*C*) Slices through the surface of cytochrome *c* reduction rate (*solid lines*) and superoxide production rate (*dashed lines*) at various Q-pool redox states and membrane potentials are shown in the top two panels. In the bottom panel, the bistability stability phenomenon is shown. (*D*) Enzyme state occupancies for these conditions reveal that higher reduced states are achieved as both the Q pool becomes reduced and the membrane potential is high. To see this figure in color, go online.

Unfortunately, kinetics using the native Q_10_ binding constants for the *bc*_1_ complex are unavailable. So, identifying the Q_10_ binding parameters is more difficult than the Q-analog binding constants for several reasons. First, there is no kinetic data available for the native substrate; there is also no reliable method to quantify the redox state of the Q pool without destruction of the sample. This makes direct quantification impossible. Second, these estimates are based on thermodynamic and structural assumptions that may not hold for the native substrate during steady-state turnover of the enzyme (61, 62, 63). However, there are data we can use as a basis for developing likely in situ binding constants. For the Q_p_ site, Ding et al. (62) used kinetic and thermodynamic arguments to conclude dissociation constants for the Q_p_ site of QH_2_ and Q are near equal and in the low millimolar range. For the Q_n_ site, Wikström (63) derives values for the both QH_2_ and Q binding constants with QH_2_ binding ∼100-fold tighter than Q. Using these results, we can derive a set of values that leads to tenable cytochrome *c* reduction and superoxide production rates shown in Fig. 5. The bell-shaped relationship between the cytochrome *c* reduction rate and Q-pool redox state (Fig. 5 *C*, *upper plot*) and the exponential dependency of superoxide production rate on membrane potential (Fig. 5 *C*, *middle plot*) is similar to our prior simulations (53). In addition, the so-called bistability phenomenon (49, 53) is still present (Fig. 5 *C*, *lower plot*); however, the relationship is qualitatively different. This phenomenon is characterized by the possibility of two free radical production rates at a given rate of cytochrome *c* reduction. But these simulations reveal that bistability also occurs at a given rate of superoxide production. This latter phenomenon is due to a significant drop in superoxide production under extremely reduced Q-pool redox states that may help minimize free radical production under these conditions. Some other notable differences between our previous simulations and the current ones are the overall shapes of the cytochrome *c* reduction and superoxide production rates as a function of membrane potential and Q-pool redox state, maximum rates of superoxide production, and the enzyme distribution surfaces (Fig. 5 *D*). For cytochrome *c* reduction, the maximum rate occurs when the Q pool is 90% reduced and the membrane potential is low (Fig. 5 *A*). But as the membrane potential increases, the maximum rate of cytochrome *c* reduction occurs when the Q-pool redox state is closer to 65% reduced (Fig. 5, *A* and *C*, *upper plot*). The superoxide production rate is highest when the Q pool is mostly reduced (75–95%) for nearly all membrane potentials (Fig. 5, *B* and *C*, *upper plot*). The enzyme state distributions show varying levels of occupancy depending on the membrane potential and Q-pool redox state (Fig. 5 *D*). Under oxidized conditions when the membrane potential is low, the most probable enzyme states are the fully oxidized state and one-electron reduced state. The two- and three-electron reduced states are elevated at high membrane potentials and when the Q pool is reduced. The four-electron reduced state becomes significant when the Q pool is extremely reduced, regardless of membrane potential. The five-electron reduced state reaches its maximum level when both the membrane potential is high and the Q pool is nearly 100% reduced, but even here the maximum level is <1%. This state only reaches significant levels when antimycin A is bound to the complex (data not shown). During cytochrome *c* reduction, the one-, two-, and three-electron reduced states are the most significant states (see Figs. S2 and S3). Other states play only a minor role in the net turnover of the enzyme. Superoxide is primarily produced through the three- and four-electron reduced states (see Fig. S4). Superoxide production from the five-electron reduced state is only important in the antimycin A-inhibited state. Finally, it is worth noting that under normal forward electron transfer conditions, the four-electron reduced state (*E*_4_, see Fig. 1 *B*) can oxidize back to *E*_*0*_ by two successive oxidization reactions by passing the four electrons to two quinone molecules bound at the Q_n_ sites, yielding two fully reduced quinols (*E*_4_ → *E*_2_ → *E*_0_, *left side diagonal branches* in Fig. 1 *B*).

### Physiological Q-pool operating range and cardiac *bc*_1_ content

The redox state of the Q pool during rest, work, and pathophysiological conditions has been debated for decades. Some studies report the pool being mostly reduced (56, 67, 81, 82), others report half-reduced (83, 84), whereas a couple report mostly oxidized (85, 86, 87). The bell-shaped relationship between cytochrome *c* reduction and the Q-pool redox state makes either possibility feasible (see Fig. 5). In addition to this variability, estimates of *bc*_1_ content range from 80 to 500 pmol/mg mitochondrial protein (85, 88, 89, 90, 91). With these uncertainties, extrapolating to an in vivo model is problematic. Fortunately, with our model, we can test 1) where the physiological operating Q-pool redox state of the *bc*_1_ complex is, and 2) the influence of *bc*_1_ protein content on cardiac mitochondria. To do this, we need to use data sets that measure all the relevant bioenergetic and product variables for the *bc*_1_ reaction.

**Figure 6:**
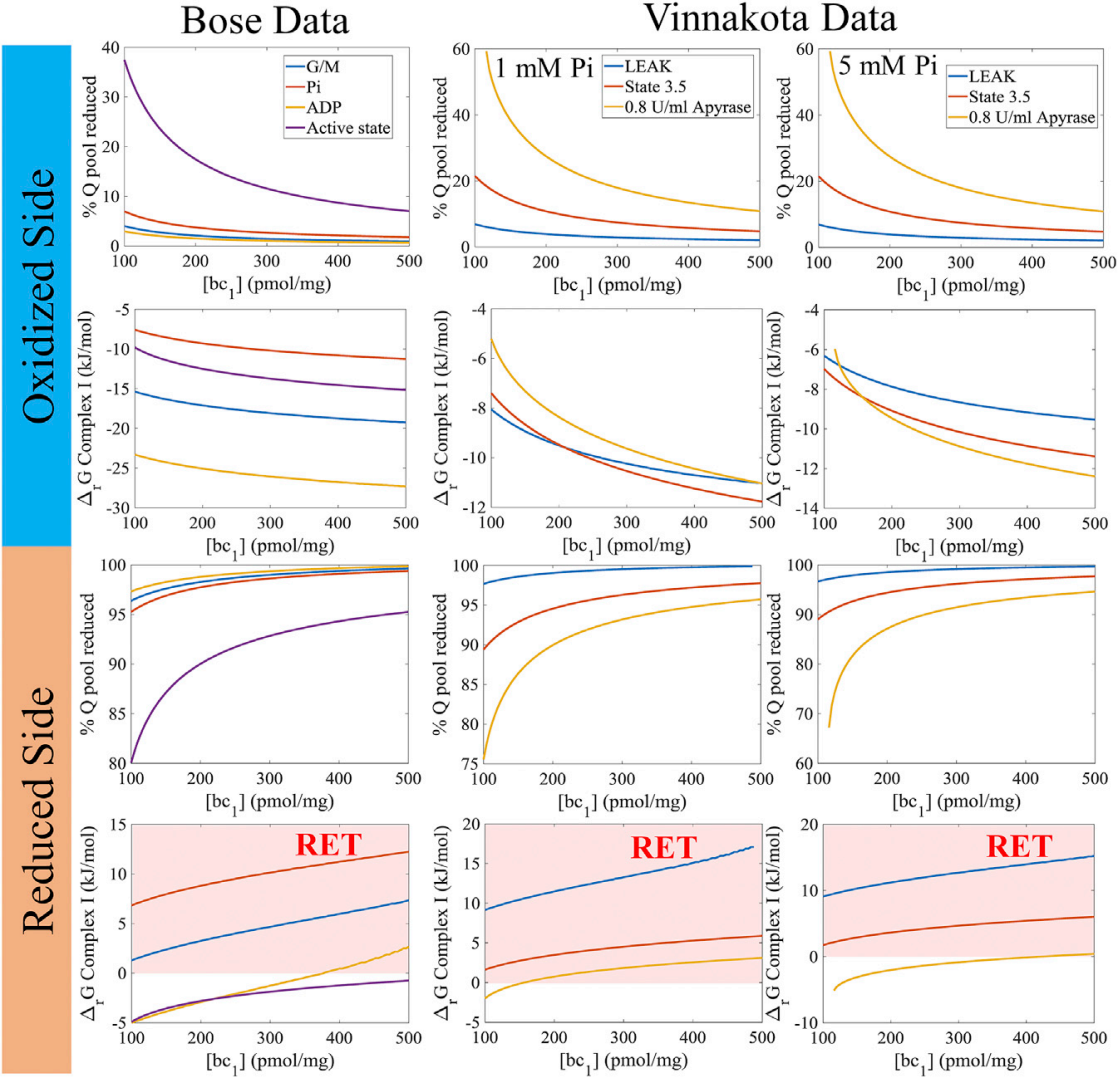
Model predictions of *bc*_1_ content and Q-pool redox state during various bioenergetics states. Data reported in either Böse et al. (92) or Vinnakota et al. (93) were used to simulate the model using native Q_10_ binding constants given in Table S1. These data include membrane potential, pH, cytochrome *c* redox state, and NADH redox state for a given oxygen consumption rate. The redox state of the Q pool (% Q reduced) was treated as a variable and solved for, to match the reported oxygen consumption rates for the given conditions. The search was started from either the oxidized side or the reduced side to generate the two possible solutions. With the calculated redox state of the Q pool, the Complex I free energy of reaction was then computed. Whenever this value is >0, the Complex I reaction reverses. The standard free energy of reaction for Complex I at pH 7 was defined as −74.3 kJ/mol (see Table S1). Incomplete line segments indicate that no solution was found for the simulated conditions. Areas shaded in red (*bottom row*) designate regions where reverse electron transport (*RET*) occurs. This is a pathological condition linked to extremely high rates of free radical production. For the Böse data set, the “G/M” label refers to a condition where mitochondria were energized in the presence of 5 mM glutamate and 5 mM malate in the absence of Pi. The “Pi” label refers to the G/M condition, but in the presence of 3 mM Pi. The “ADP” label refers to the G/M condition in the presence of 1.3 mM ADP. The “Active state” label refers to the G/M conditions in the presence of 3 mM Pi and 1.3 mM ADP. For the Vinnakota data set, the “LEAK” label refers to a condition where mitochondria were energized in the presence of 2.5 mM pyruvate, 0.5 mM malate, and 5 mM ATP in the presence of 19 U/mL pyruvate kinase and 2 mM phosphoenolpyruvate to maximize the ATP/ADP ratio. The “State 3.5” label refers to the LEAK condition without pyruvate kinase and phosphoenolpyruvate where mitochondria are respiring to meet ATP demand due to residual ATPase activity in the preparation. The “0.8 U/mL Apyrase” label refers to the State 3.5 condition in the presence of 0.8 U/mL apyrase to maximally stimulate oxidative phosphorylation. For more details concerning the relevant mitochondrial variables and experimental conditions, see referenced studies. To see this figure in color, go online.

Very few such data sets exist; in our search, we only found two, the Böse data set (92) and the Vinnakota data set (93). Each data set probes mitochondrial function while monitoring essential *bc*_1_ complex variables. They both report the rate of oxygen consumption (stoichiometrically linked to cytochrome *c* reduction), membrane potential, pH, and redox state of the cytochrome *c* pool. In addition, the NADH redox state is given for each condition. This variable is important to rule out nonphysiological redox states of the Q pool using a thermodynamic argument. Therefore, we can use these data sets to identify the Q-pool redox states capable of explaining the data for a wide range of *bc*_1_ content. Fig. 6 shows model simulations using these data sets as fixed inputs for the model (cytochrome *c* redox state, membrane potential, and pH) while solving for the Q-pool redox state as a variable for each value of the *bc*_1_ content to match the reported oxygen consumption rates (proportional to the turnover of the *bc*_1_ complex). Because the QH_2_-dependent kinetics are biphasic (parabolic, see Fig. 5 *A* and *upper panel* of Fig. 5 *C*), the model can explain the data for either a reduced or oxidized state of the Q pool. But when the Q pool is on the reduced side, Complex I will be operating in the so-called reverse electron transport mode (52, 94). This mode is characteristic of extreme levels of superoxide production and only relevant in the disease state (95). Thus, we can infer that the normal operating redox state for the Q pool is that of mostly being oxidized when reducing substrates like glutamate and malate, or pyruvate, are present in excess. Also, the model simulations reveal that the physiological fluxes through the *bc*_1_ complex in heart can be explained using *bc*_1_ content in the range of 115–500 pmol/mg mitochondria. Below a *bc*_1_ content of 115 pmol/mg, the model could not match the reported oxygen consumption rate for any Q-pool redox state using the inputs given by the data for the Vinnakota data set (see Fig. 5, *right column panels*). This range cannot be narrowed further due to the tight correlation between the *bc*_1_ content and Q-pool redox state. To precisely quantify the *bc*_1_ content in heart using these data, we would need either a direct measure of the *bc*_1_ content in these preparations or data on the Q_10_ redox state for the experimental conditions used in these studies. Because the Q_10_ dissociation constants are only estimates, the more informative measurement would be the Q_10_ redox state. Unfortunately, this variable cannot be precisely quantified using current methods. Once we are able to make this measurement, we will have a more complete picture of the bioenergetic behavior of how the mitochondrial respiratory chain operates in both health and disease.

In summary, a functional dimer model of the *bc*_l_ complex is presented. The model is capable of simulating the enzyme kinetics under a wide range of conditions and is calibrated with superoxide production data obtained using the purified complex and antimycin A-treated mitochondria. It was determined that Coulombic effects between intramonomer heme *b*_L_ and heme *b*_H_ and between intermonomer heme *b*_L_ and heme *b*_L_ were required to fit the data with a single, consistent set of parameters. In addition, model analysis supports the hypothesis that in normal steady-state conditions, only a single *Q*_p_ site in the dimer is operational per quinol oxidation. Model analysis demonstrates that the semireverse mode of superoxide production constitutes the major mechanism of free radical production by the *bc*_1_ complex. The model also reveals that under physiological conditions, the Q pool is primarily in the oxidized state. However, in the presence of succinate or under pathological conditions, it can reach a significantly reduced state. Furthermore, the model was developed for the purpose of simulating mitochondrial metabolism as part of large-scale models. As this model is better calibrated and more faithful to the biophysics of the reaction compared to our previous models, more accurate simulations of free radical generation by the respiratory system will be possible.

## Supporting Citations

References (96, 97, 98, 99, 100, 101, 102, 103, 104, 105, 106, 107) appear in the Supporting Material.

## Supporting Material

Document S1. Supporting Materials and Methods, Figs. S1–S9, and Tables S1 and S2

Data S1. Model Code

Document S2. Article plus Supporting Material
