# Supplementary material for: Analysis of a Functional Dimer Model of Ubiquinol Cytochrome c Oxidoreductase
Source: Biophys J. 2017 Oct 3;113(7):1599–612. doi: 10.1016/j.bpj.2017.08.018 (PMC5627346; doi:10.1016/j.bpj.2017.08.018)
Supplement: Document S1. Supporting Materials and Methods, Figs. S1–S9, and Tables S1 and S2 [file mmc1.pdf]

**Biophysical Journal, Volume *113***

## Supplemental Information

# Analysis of a Functional Dimer Model of Ubiquinol Cytochrome *c* Oxidoreductase

**Jason N. Bazil**

# Supporting Material

Analysis of a Functional Dimer Model of Ubiquinol Cytochrome c Oxidoreductase

Jason N. Bazil

Michigan State University, Department of Physiology, East Lansing, MI

Table S1 shows the fixed model parameters used in the simulations given in the main text and in this supplement.

Table S1. Fixed Parameters

| Parameter | Definition | Value[a] | Units | Reference |
|---|---|---|---|---|
| *Physical Constants* | | | | |
| $R$ | Ideal gas constant | 8.314 | J/mol/K | - |
| $F$ | Faraday's constant | 96.5 | J/mol/mV | - |
| *$K_A$ values* | | | | |
| $K_A^{ISPox}$ | Oxidized Rieske iron-sulfur cluster acidic group protonation constant | $10^{-6.6}$ | - | (1) |
| $K_B^{ISPox}$ | Oxidized Rieske iron-sulfur cluster basic group protonation constant | $10^{-9.2}$ | - | (1) |
| $K_A^{b_L ox}$ | Oxidized heme $b_L$ protonation constant | $10^{-5.9}$ | - | (2) |
| $K_A^{b_L red}$ | Reduced heme $b_L$ protonation constant | $10^{-7.9}$ | - | (2) |
| $K_A^{b_H ox}$ | Oxidized heme $b_H$ protonation constant | $10^{-5.7}$ | - | (2) |
| $K_A^{b_H red}$ | Reduced heme $b_H$ protonation constant | $10^{-7.7}$ | - | (2) |
| *Thermodynamic* | | | | |
| $E_m(c^{3+}/c^{2+})$ | Cytochrome c midpoint potential | 255 | mV | (3) |
| $E_m(c_1^{3+}/c_1^{2+})$ | Cytochrome $c_1$ midpoint potential | 270 | mV | (4) |
| $E_m^7(ISP_{ox}/ISP_{red})$ | Rieske ISP midpoint potential | 300 | mV | (4) |
| $E_m^0(Q/QH_2)$ | Ubiquinone midpoint potential | 65 (479) | mV | (5) |
| $E_m^0(b_L^{3+}/b_L^{2+})$ | Cytochrome $b_L$ midpoint potential | -30 (39) | mV | (2) |
| $E_m^0(b_H^{3+}/b_H^{2+})$ | Cytochrome $b_H$ midpoint potential | 90 (160) | mV | (2) |
| $E_m^0(Q/SQ)_n$ | Qn-site semiquinone midpoint potential | -2.3 | mV | (6) |
| $E_m^0(SQ/QH_2)_n$ | Qn-site semiquinone midpoint potential | 122 (951) | mV | (6) |
| $E_m^7(NAD^+/NADH)$ | NADH midpoint potential | -320 | mV | (7) |
| $\beta$ | Fraction of total charge translocation sensed between bL and bH | 0.5 | - | (2, 8-10) |
| *$Q_{10}$ Related Constants* | | | | |
| $K_{DQ_p}^{Q_{10}H_2}$ | $Q_{10}H_2$ binding constant at Qp-site | 1.6 | mM | (11) |
| $K_{DQ_p}^{Q_{10}}$ | $Q_{10}$ binding constant at Qp-site | 1.6 | mM | (11) |

| | | | | |
|---|---|---|---|---|
| $K_{DQ_n}^{Q_{10}}$ | $Q_{10}$ binding constant at Qn-site | 0.25 | mM | (12)[b] |
| $K_{DQ_n}^{Q_{10}H_2}$ | $Q_{10}H_2$ binding constant at Qn-site | 2.5 | µM | (12)[b] |
| $Q_{10,tot}$ | $Q_{10}$ pool size | 20 | mM | (13, 14)[c] |
| *Superoxide* | | | | |
| $E_m(O_2/O_2^{\bullet-})$ | Superoxide midpoint potential | -160 | mV | (15)[d] |
| $kf_{SO}$ | Superoxide production 2nd order rate constant | $10^{10}$ | $M^{-1}s^{-1}$ | (16) |

[a]Values are at pH 7 and at 25 °C. Values in parenthesis are at pH 0 and 25 °C. [b]Values chosen to match reported kinetic and thermodynamic behavior of enzyme. [c]Estimated by assuming a total mitochondrial Q pool of 5.8 nmol/mg and an inner membrane volume of 290 nl/mg. [d]The pK of superoxide is 4.7, so the midpoint potential is invariant in the biological pH range.

Table S2. Experimental Data Summary

| Species and Tissue Origin | Quinone Substrate | Kinetic Data | Buffer pH | Membrane Potential (mV) | Free Radical Data | Dimeric Function Data | Reference |
|---|---|---|---|---|---|---|---|
| Bovine heart | DQ | yes | 7.5 | 0 | no | no | (17) |
| Bovine heart | NBH | yes | 7.2 | 0 | no | no | (1) |
| Bovine heart | $Q_2$ | yes | 7.4 | 0 | no | no | (18) |
| Bovine heart | $Q_2$ | yes | 7.4 | 0 | no | no | (19) |
| Rat liver | $Q_2$ and $Q_{10}$ | yes | 7.0 | 100 - 180 | no | no | (20) |
| Yeast | DQ | no | 7.3 | 100 - 220 | yes | no | (21) |
| Rat skeletal muscle | $Q_{10}$ | no | 7.2 | 0 | yes | no | (22) |
| Yeast | DQ | no | 7.0 | 0 | no | yes | (23) |
| Bovine heart | DQ | no | 7.4 | 0 | yes | no | (24) |

$Q_2$, ubiquinone-2; NBH, nonylubihydroquinone; DQ, decylubiquinone; $Q_{10}$, ubiquonone-10

Table S2 lists the data used to parameterize the model. The list consists of a set of data that contain information on the cytochrome c reduction kinetics and superoxide production rates collected under a wide range of conditions. This is not an exhaustive list of all available data, but it does contain data that enables identification of essential features of bc1 kinetics and free radical generation. The majority of the data come from bc1 complex purified from bovine heart, but some of the superoxide production and dimeric function data were obtained using rat and yeast mitochondria. Ideally, these data would be from the same source as the kinetic data. But unfortunately, we could not find such data. The data was collected using bc1 complex resuspended in detergent, incorporated into lipid membranes, or in its original state in mitochondrial membranes. The kinetic data were collected with a variety of Q-analogues at different pH and membrane energization conditions. The superoxide production data was collected in a similar manner. By fitting all these data with the same set of rate constants and relevant kinetic parameters, the robustness of the model is demonstrated.

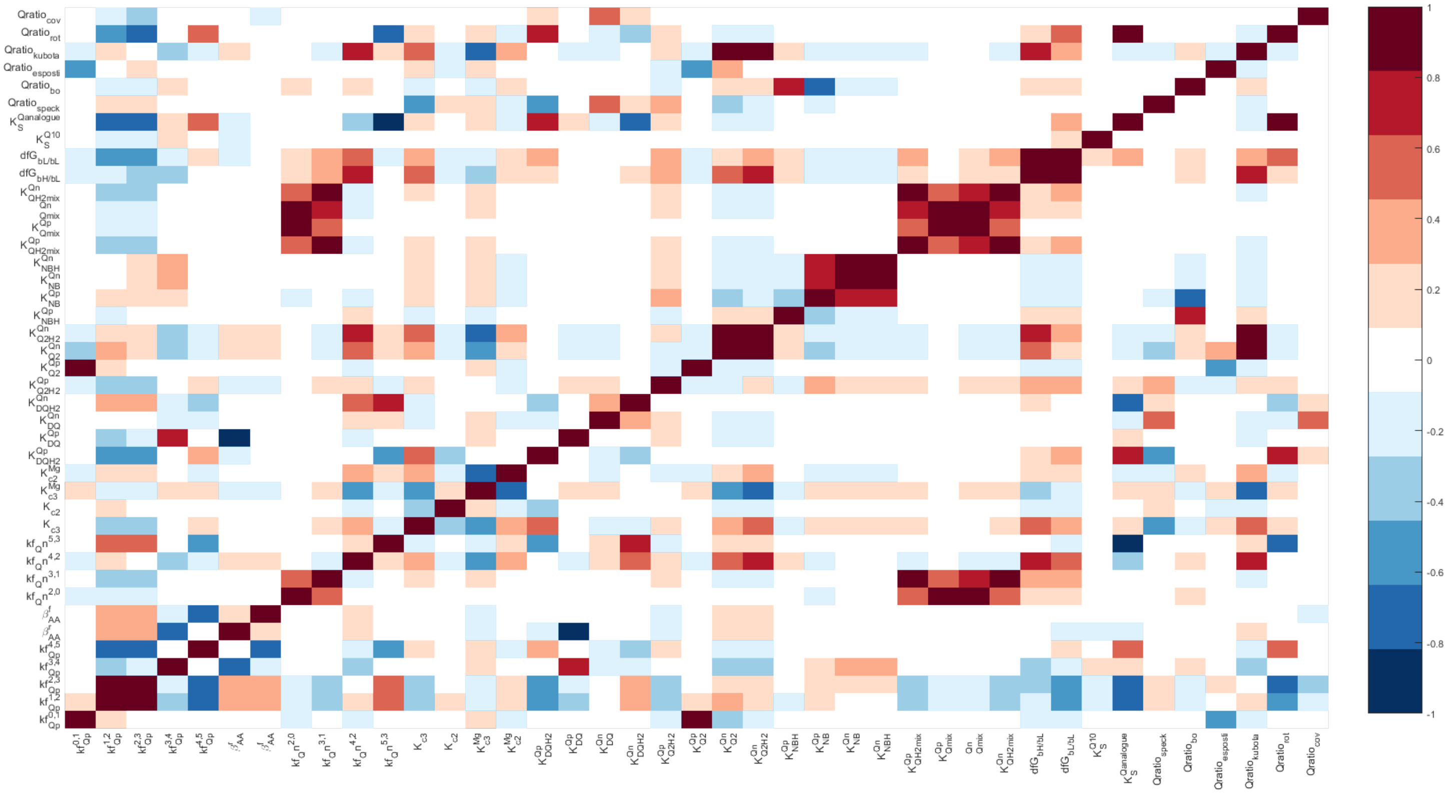


**Figure S1. Correlation heat map for adjustable parameters given in Table 1.** Parameter correlation coefficients are ordered in the following manner: state transition rate constants, cytochrome c binding constants, quinone analogue binding constants, quinone stability constants, and experimental design parameters. Parameter correlation coefficients were computed from the parameter sensitivity matrix (see Eq. S44). Parameters can be negatively correlated (red pixels, coefficient value of -1), uncorrelated (white pixels, coefficient value of 0), or positively correlated (blue pixels coefficient value of 1).

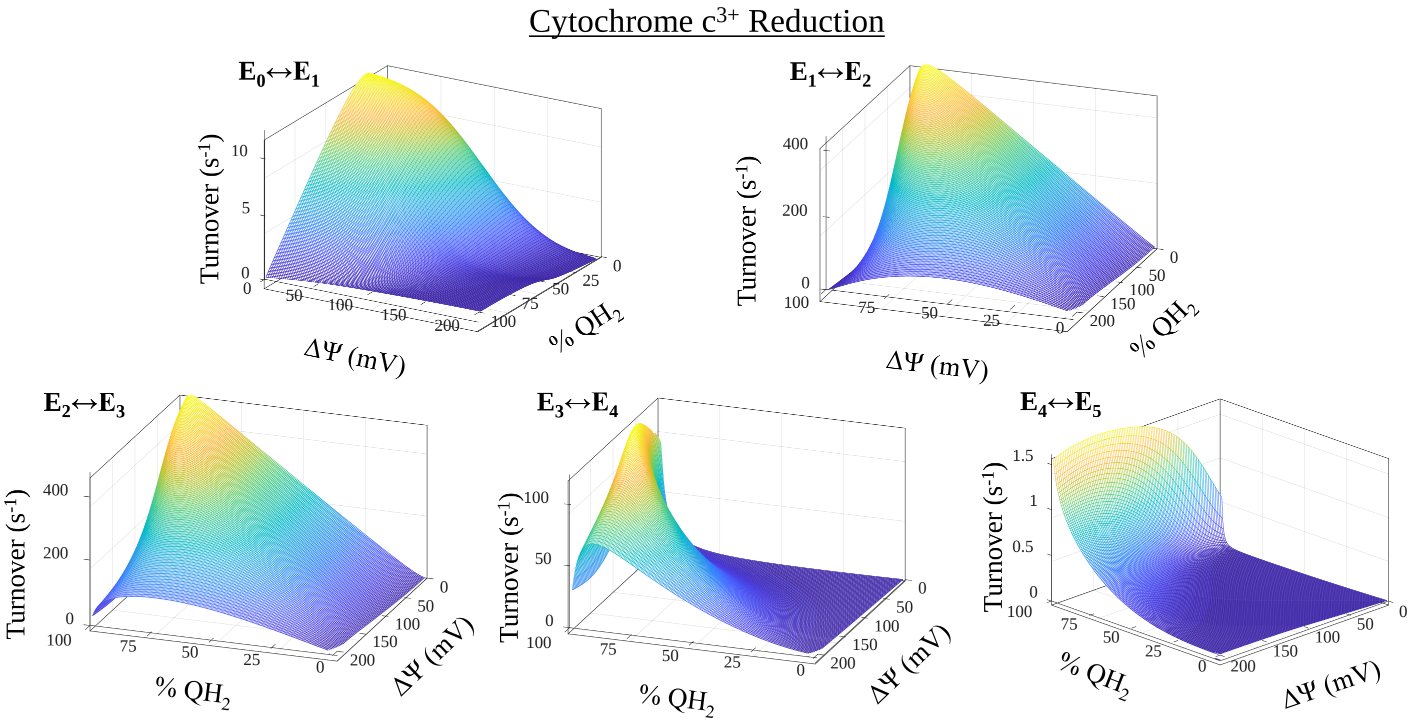


**Figure S2. Net cytochrome $c^{3+}$ reduction turnover rates at the Qp-site.** The partial reaction for each enzyme state transition is given as indicated. The conditions for these simulations are given in Figure 5.

# Quinone Reduction

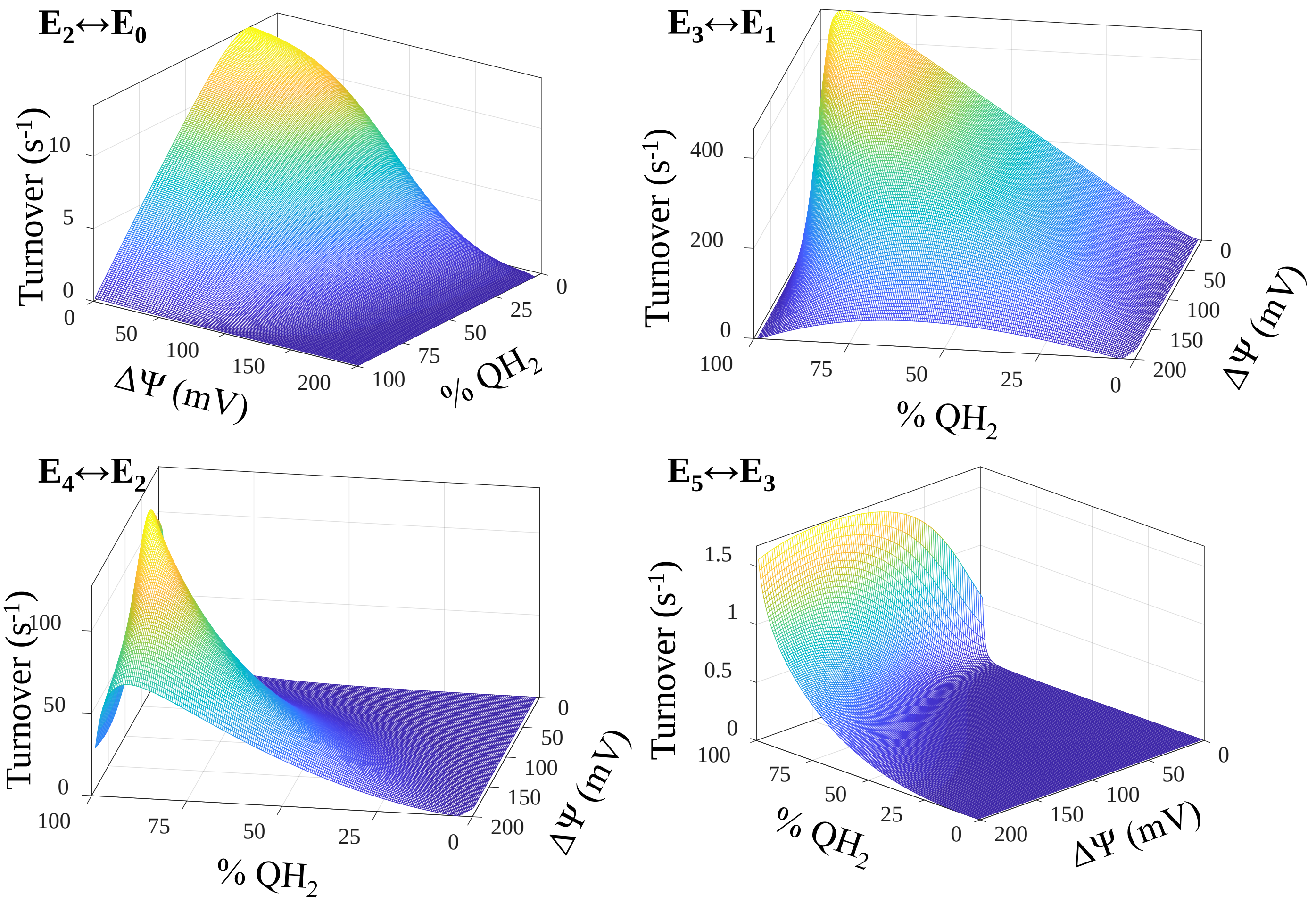


**Figure S3. Net quinone reduction turnover rates at the Qn-site.** The partial reaction for each each enzyme state transition is given as indicated. The conditions for these simulations are given in Figure 5.

Superoxide Production

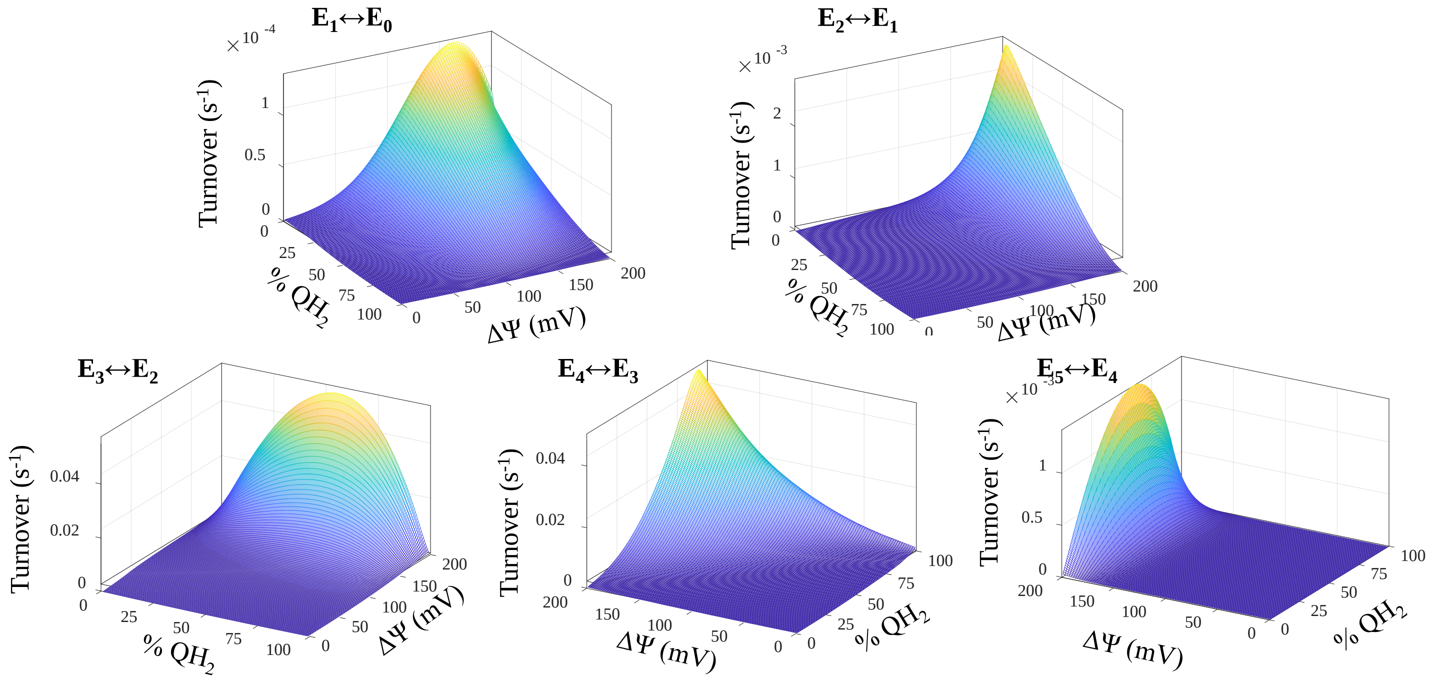


**Figure S4. Net superoxide production turnover rates at the Qp-site.** The partial reaction for each enzyme state transition is given as indicated. The conditions for these simulations are given in Figure 5.

*Midpoint potential to free energy conversions.* The midpoint potentials given in Table S1 were converted to free energies using the following equation.

$$\Delta G = -nF\Delta E \qquad \text{Eq. S1}$$

where *n* is the number of electrons transferred in the reaction.

*Free energy of electron transfer and pH effects.* The free energy of reaction for the mobile electron carriers (quinones and cytochrome c) are defined with respect to their respective pools. So the free energy of electron transfer (midpoint potentials when expressed in mV) for these electron carriers must be adjusted for the free energy of binding and unbinding to the bc1 complex. For the cytochrome $c^{3+}/c^{2+}$ reduction free energy, the free energy of electron transfer is calculated using Eq. S2.

$$\Delta_{et}G_{c^{3+}/c^{2+}} = \Delta G^{0}_{c^{3+}/c^{2+}} - RT\ln(KD_{c^{3+}}) + RT\ln(KD_{c^{2+}}) \qquad \text{Eq. S2}$$

For the quinone/semiquinone and the semiqinone/quinol reduction free energy, the free energy of electron transfer is calculated in a similar manner using Eqs. S3 and S4.

$$\Delta_{et}G_{Q/SQ} = \Delta G^{0}_{Q/SQ} - RT\ln(K_{Q}) \qquad \text{Eq. S3}$$

$$\Delta_{et}G_{SQ/QH_2} = \Delta G^{pH}_{SQ/QH_2} + RT\ln(K_{QH_2}) \qquad \text{Eq. S4}$$

The free energy for the semiquinone/quinol couple is pH dependent and calculated using Eq. S5.

$$\Delta G^{pH}_{SQ/QH_2} = \Delta G^{0}_{SQ/QH_2} - 2RT\ln([H^+]) \quad \text{Eq. S5}$$

For the semiquinone/quinol couple at the Qp-site, the proton concentration is taken with respect to the positive side of the membrane ($[H^+]_p$). For the Qn-site, the proton concentration is taken with respect to the negative side of the membrane ($[H^+]_n$).

In a similar manner, the quinone/quinol couple free energy is pH dependent and calculated using Eq. S6.

$$\Delta G^{pH}_{Q/QH_2} = \Delta G^{0}_{Q/QH_2} - RT\ln([H^+]) \quad \text{Eq. S6}$$

The free energy of electron transfer for the b hemes are also pH-dependent but possess pKs close to neutral pH. These free energies are computed using Eqs. S7 and S8.

$$\Delta G^{pH}_{b_L^{ox}/b_L^{red}} = \Delta G^{0}_{b_L^{ox}/b_L^{red}} + RT\ln\left(([H^+]+10^{-pK_{bLox}})/([H^+]+10^{-pK_{bLred}})\right) \quad \text{Eq. S7}$$

$$\Delta G^{pH}_{b_H^{ox}/b_H^{red}} = \Delta G^{0}_{b_H^{ox}/b_H^{red}} + RT\ln\left(([H^+]+10^{-pK_{bHox}})/([H^+]+10^{-pK_{bHred}})\right) \quad \text{Eq. S8}$$

For the heme $b_L$, the proton concentration is taken with respect to the positive side of the membrane. And for heme $b_H$, the proton concentration is taken with respect to the negative side of the membrane.

*Semiquinone stability constants and free energies.* To calculate the semiquinone free energies from the semiquinone stability constant and ubiquinone/ubiquinol couple free energy, the following relations were used:

$$\Delta G^{pH}_{Q/QH_2} = \Delta G_{Q/SQ} + \Delta G^{pH}_{SQ/QH_2} \quad \text{Eq. S9}$$

$$\Delta G_{Q/SQ} - \Delta G^{pH}_{SQ/QH_2} = -RT\ln(K_S) \quad \text{Eq. S10}$$

*Equilibrium constants.* The equilibrium constants for electron transfer for quinol oxidation at the Qp-site, quinone reduction at the Qn-site, and superoxide production at the Qp-site are given below.

$$K^{eq}_{Qp} = e^{-(\Delta_{et}G_{c^{3+}/c^{2+}} - \Delta_{et}G^{Qp}_{SQ/QH_2})/RT} \quad \text{Eq. S11}$$

$$K^{eq}_{Qn} = e^{-(\Delta_{et}G^{Qp}_{SQ/QH_2} - \Delta G^{pH}_{b_H^{ox}/b_H^{red}})/RT} \quad \text{Eq. S12}$$

$$K^{eq}_{SO} = e^{-(\Delta G^{0}_{O_2/O_2^{\cdot-}} - \Delta_{et}G^{Qp}_{SQ/QH_2})/RT} \quad \text{Eq. S13}$$

*Substate distribution.* For each state (oxidized to five-electron reduced), the complex exists in a combination of redox states that depends on the total number of reachable redox centers and the number of electrons on the complex. To account for Coulombic repulsion forces, we include neighbor interactions for intra-monomer $b_L/b_H$ and inter-monomer $b_L/b_L$ pairs. For details, see below.

monomer a

$$\Delta G^{a}_{Q/SQn} = \Delta_{et} G_{Q/SQn} - RT \ln\left([Q]/K^{Qn}_{Q}/P_{Qn}\right) \qquad \text{Eq. S14}$$

If an antimycin A molecule is bound to the Qn-site of monomer a, Eq. S14 becomes

$$\Delta G^{a}_{Q/SQn} = \infty \qquad \text{Eq. S15}$$

$$\Delta G^{a}_{b_H^{ox}/b_H^{red}} = \Delta G^{pH}_{b_H^{ox}/b_H^{red}} + 2F\beta\Delta\Psi \qquad \text{Eq. S16}$$

$$\Delta G^{a}_{b_L^{ox}/b_L^{red}} = \Delta G^{pH}_{b_L^{ox}/b_L^{red}} \qquad \text{Eq. S17}$$

$$\Delta G^{a}_{Q/SQp} = \Delta_{et} G_{Q/SQp} - RT \ln\left([Q]/K^{Qp}_{Q}/P_{Qp}\right) \qquad \text{Eq. S18}$$

monomer b

$$\Delta G^{b}_{Q/SQn} = \Delta_{et} G_{Q/SQn} - RT \ln\left([Q]/K^{Qn}_{Q}/P_{Qn}\right) \qquad \text{Eq. S19}$$

If an antimycin A molecule is bound to the Qn-site of monomer b, Eq. S19 becomes

$$\Delta G^{b}_{Q/SQn} = \infty \qquad \text{Eq. S20}$$

$$\Delta G^{b}_{b_H^{ox}/b_H^{red}} = \Delta G^{pH}_{b_H^{ox}/b_H^{red}} + 2F\beta\Delta\Psi \qquad \text{Eq. S21}$$

$$\Delta G^{b}_{b_L^{ox}/b_L^{red}} = \Delta G^{pH}_{b_L^{ox}/b_L^{red}} \qquad \text{Eq. S22}$$

$$\Delta G^{b}_{Q/SQp} = \infty \qquad \text{Eq. S23}$$

If a single antimycin A molecule is bound to the dimer, then Eq. S23 becomes

$$\Delta G^{b}_{Q/SQp} = \Delta_{et} G_{Q/SQp} - RT \ln\left([Q]/K^{Qp}_{Q}/P_{Qp}\right) \qquad \text{Eq. S24}$$

For the Coulombic electrostatic interactions, energy penalties were added to the redox state combinations containing intra-monomer $b_L/b_H$ and inter-monomer $b_L/b_L$ pairs. These energies are given in Table 1. However, in order to satisfy detailed

balance, this penalty was not applied to redox centers including intra-monomer $b_L$-$b_H$-SQn combinations for three-electron reduced states and higher.

To calculate the substate distribution for each enzyme state, we need all the possible combinations of redox centers. To do this, we use combinatorics. For example, the two-electron reduced dimer has a total of 8!/2!/(8-2)! = 28 substate combinations, where 8 is the total number of possible redox centers and 2 is the number of electrons on the dimer. To calculate the standard free energy change for each redox center combination, we assume linear combinations of the individual standard free energy change for each redox center described by Eqs. S14-S24. We then use the Boltzmann distribution to calculate the reduced fraction of each redox center combination on the dimer with a given number of electrons.

*State occupancy solution.* For the state occupancies, Eq. S25 was solved. For brevity, only the matrix equation is shown. The analytical solution consists of several thousand terms. Electrons are added or removed from the complex either one at a time at the Qp-site or two at a time at the Qn-site. The last row in Eq. S25 is a conservation equation to fix the sum of the state occupancies to equal one.

$$\begin{bmatrix} -(k_{01}+k_{02}) & k_{10} & k_{20} & 0 & 0 & 0 \\ k_{01} & -(k_{10}+k_{12}+k_{13}) & k_{21} & k_{31} & 0 & 0 \\ k_{02} & k_{12} & -(k_{20}+k_{21}+k_{23}) & k_{32} & k_{42} & 0 \\ 0 & k_{13} & k_{23} & -(k_{31}+k_{32}+k_{34}) & k_{43} & k_{53} \\ 0 & 0 & k_{24} & k_{34} & -(k_{42}+k_{43}+k_{45}) & k_{54} \\ 0 & 0 & 0 & k_{35} & k_{45} & -(k_{53}+k_{54}) \\ 1 & 1 & 1 & 1 & 1 & 1 \end{bmatrix} \begin{bmatrix} E_0 \\ E_1 \\ E_2 \\ E_3 \\ E_4 \\ E_5 \end{bmatrix} = \begin{bmatrix} 0 \\ 0 \\ 0 \\ 0 \\ 0 \\ 1 \end{bmatrix} \qquad \text{Eq. S25}$$

*State transition rate constants.* The state transition rate constants, $k_{i,j}$, are the summation of the partial one-step and two-step oxidation/reduction reactions. They include both the forward and reverse reactions that facilitate transitions from state *i* to state *j*. For completeness, they are given below in Eqs. S26-S43. These equations are for the antimycin A free turnover conditions. Therefore, only one Qp-site is active. When antimycin A is bound to a single monomer, both are active (23). Both Qn-sites are available for turnover except when antimycin A is bound. The superscript *a* and *b* designates which monomer in the dimer is involved in the partial reaction. The equations for the binding terms $B_f$, $B_r$, and $B_Q$ are given in Eqns. S48-S54.

$$k_{01} = kf_{Q_p}^{0,1} B_f + B_Q [O_2^{\cdot-}] kr_{SO} \qquad \text{Eq. S26}$$

$$k_{10} = \left( kr_{Q_p}^{0,1} B_r + [O_2] kf_{SO} \right) s^1_{r \in \{SQ_p^a\}} \qquad \text{Eq. S27}$$

$$k_{12} = \left( kf_{Q_p}^{1,2} B_f + B_Q [O_2^{\cdot-}] kr_{SO} \right) \sum_{\forall r \notin \{SQ_p^a\}} s_r^1 \qquad \text{Eq. S28}$$

$$k_{21} = \left( kr_{Q_p}^{1,2} B_r + [O_2] kf_{SO} \right) \sum_{\forall r \in \{SQ_p^a\}} s_r^2 \qquad \text{Eq. S29}$$

$$k_{20} = e^{-2\beta\Delta\Psi/2RT} kf_{Q_n}^{2,0} \left( s^2_{r \in \left\{ b_L^{red,a} \cdot SQ_n^a \right\}} + s^2_{r \in \left\{ b_L^{red,b} \cdot SQ_n^b \right\}} \right)$$ Eq. S30

$$k_{02} = 2e^{2\beta\Delta\Psi/2RT} kr_{Q_n}^{2,0}$$ Eq. S31

$$k_{23} = \left( kf_{Q_p}^{2,3} B_f + B_Q [O_2^-] kr_{SO} \right) \sum_{\forall r \notin \left\{ SQ_p^a \right\}} s_r^2$$ Eq. S32

$$k_{32} = \left( kr_{Q_p}^{2,3} B_r + [O_2] kf_{SO} \right) \sum_{\forall r \in \left\{ SQ_p^a \right\}} s_r^3$$ Eq. S33

$$k_{31} = e^{-2\beta\Delta\Psi/2RT} kf_{Q_n}^{3,1} \left( s^3_{r \in \left\{ b_L^{red,a} \cdot SQ_n^a \right\}} + s^3_{r \in \left\{ b_L^{red,b} \cdot SQ_n^b \right\}} \right)$$ Eq. S34

$$k_{13} = e^{2\beta\Delta\Psi/2RT} kr_{Q_n}^{3,1} \left( \sum_{r \notin \left\{ b_L^{red,a}, SQ_n^a \right\}} s_r^1 + \sum_{r \notin \left\{ b_L^{red,b}, SQ_n^b \right\}} s_r^1 \right)$$ Eq. S35

$$k_{34} = \left( kf_{Q_p}^{3,4} B_f + B_Q [O_2^-] kr_{SO} \right) \sum_{\forall r \notin \left\{ SQ_p^a \right\}} s_r^3$$ Eq. S36

$$k_{43} = \left( kr_{Q_p}^{3,4} B_r + [O_2] kf_{SO} \right) \sum_{\forall r \in \left\{ SQ_p^a \right\}} s_r^4$$ Eq. S37

$$k_{42} = e^{-2\beta\Delta\Psi/2RT} kf_{Q_n}^{4,2} \left( s^4_{r \in \left\{ b_L^{red,a} \cdot SQ_n^a \right\}} + s^4_{r \in \left\{ b_L^{red,b} \cdot SQ_n^b \right\}} \right)$$ Eq. S38

$$k_{24} = e^{2\beta\Delta\Psi/2RT} kr_{Q_n}^{4,2} \left( \sum_{r \notin \left\{ b_L^{red,a} \cdot SQ_n^a \right\}} s_r^2 + \sum_{r \notin \left\{ b_L^{red,b} \cdot SQ_n^b \right\}} s_r^2 \right)$$ Eq. S39

$$k_{45} = \left( kf_{Q_p}^{4,5} B_f + B_Q [O_2^-] kr_{SO} \right) \sum_{\forall r \notin \left\{ SQ_p^a \right\}} s_r^4$$ Eq. S40

$$k_{54} = \left( kr_{Q_p}^{4,5} B_r + [O_2] kf_{SO} \right) \sum_{\forall r \in \{SQ_p^a\}} s_r^5 \quad \text{Eq. S41}$$

$$k_{53} = e^{-2\beta\Delta\Psi/2RT} kf_{Q_n}^{5,3} \left( s^5_{r \in \{b_L^{red,a} \cdot SQ_n^a\}} + s^5_{r \in \{b_L^{red,b} \cdot SQ_n^b\}} \right) \quad \text{Eq. S42}$$

$$k_{35} = e^{2\beta\Delta\Psi/2RT} kr_{Q_n}^{3,5} \left( \sum_{r \notin \{b_L^{red,a} \cdot SQ_n^a\}} s_r^3 + \sum_{r \notin \{b_L^{red,b} \cdot SQ_n^b\}} s_r^3 \right) \quad \text{Eq. S43}$$

*Steady State Equations.* State transitions occur from the appropriate substate fraction with rate constants determined by model fitting. Net turnover at each site is computed using the following equations.

$$J_c = B_f \sum_i k_{i,i+1} E_i \sum_{\forall r \notin \{SQ_p\}} s_r^i - B_r \sum_i k_{i+1,i} E_{i+1} \sum_{\forall r \in \{SQ_p\}} s_r^{i+1}, \quad \text{Eq. S44}$$

$$J_{SO} = [O_2] kf_{SO} \sum_i E_i \sum_{\forall r \in \{SQ_p\}} s_r^i - B_Q [O_2^{\cdot -}] kr_{SO} \sum_i E_{i+1} \sum_{\forall r \notin \{SQ_p\}} s_r^{i+1}, \quad \text{Eq. S45}$$

$$J_{Qp} = J_c, \quad \text{Eq. S46}$$

and

$$J_{Qn} = \left( J_c - J_{SO} \right) / 2 \quad \text{Eq. S47}$$

where $i$ is for the $i$th electronic state given by solving Eq. S25. State transitions simulated by the model are represented in Fig. 1B. The net cytochrome c reduction ($J_c$) and superoxide production ($J_{SO}$) rates are given by Eqns. S44 and S45, respectively. Quinol oxidation at the Qp-site ($J_{Qp}$) and quinone reduction at the Qn-site ($J_{Qn}$) are stoichiometrically linked to the cytochrome c reduction and superoxide production rates as shown in Eqs. S46 and S47, respectively. Only one Qp-site is active on the dimer unless a single antimycin A molecule is bound to the dimer (23). Substrate, product, and proton bound complexes are computed using $B_f$, $B_r$, and $B_Q$ as shown in Eqs. S48-S54. Proton concentration for the ISP protonation state given in $B_f$ is taken with respect to the positive side of the membrane. For these equations, we assume substrate, product, and proton bound and unbound states are in rapid equilibrium. In addition, we assume the $c^{2+}$ and $c^{3+}$ compete with each other for the $c_1$-site, and $QH_2$ and Q compete with each other for the Qp-site and Qn-site, respectively. And for state transitions to occur, the enzyme needs to be in the proper enzyme-substrate complex (e.g., for Qp-site catalysis, the enzyme needs to be bound with $QH_2$ and $c^{3+}$; recall that we lump $QH_2$ oxidation and $c^{3+}$ reduction into a single step for simplicity).

$$B_f = [QH_2] / K_{QH2}^{Qp} / P_{Qp} \cdot [c^{3+}] / K_{c3+} / P_c \cdot [H^+] / K_B^{ISPox} / P_{ISP} \quad \text{Eq. S48}$$

$$B_r = [c^{2+}]/K_{c2+}/P_c \cdot [H^+]/K_B^{ISPox}/P_{ISP} \qquad \text{Eq. S49}$$

$$B_Q = [Q]/K_Q^{Qp}/P_{Qp} \qquad \text{Eq. S50}$$

$$P_{ISP} = \left(1+[H^+]/K_A^{ISPox}\right)\left(1+[H^+]/K_B^{ISPox}\right) \qquad \text{Eq. S51}$$

$$P_{Qp} = 1+[QH_2]/K_{QH2}^{Qp}+[Q]/K_Q^{Qp} \qquad \text{Eq. S52}$$

$$P_{Qn} = 1+[QH_2]/K_{QH2}^{Qn}+[Q]/K_Q^{Qn} \qquad \text{Eq. S53}$$

$$P_c = 1+[c^{3+}]/K_{c3+}+[c^{2+}]/K_{c2+} \qquad \text{Eq. S54}$$

*Parameter sensitivity matrix and correlation coefficients*. The normalized parameter sensitivity matrix is computed using Eq. S55. Each model output, $f_i$, is congruent with the experimental data. The parameter sensitivities were computed using a complex variable approach (25).

$$S_{i,j} = \frac{\partial f_i}{\partial p_j}\frac{p_j}{f_i} \qquad \text{Eq. S55}$$

The sensitivity coefficients presented in Table 1 were computed by averaging all the non-zero sensitivity coefficient for a given parameter. This was done by using Eq. S56.

$$\overline{s_j} = \frac{1}{N_j}\sum_{\forall i:S_{i,j}\neq 0} S_{i,j} \qquad \text{Eq. S56}$$

where $N_j$ is the number of non-zero elements in $j$th column of $S$. The parameter correlation coefficients are pairwise linear correlation coefficients computed for each pair of columns in the normalized parameter sensitivity matrix.

*Antimycin inhibited state transition rate constants.* When antimycin A is bound to a given monomer, we assume the rate of $QH_2$ oxidation at the Qp-site is slowed. This is discussed in more detail in the main text. The inhibition factors, $\beta_{AA}^r$ and $\beta_{AA}^f$, are given in Table 1. The antimycin A binding constant, $K_{AA}$, is 30 pM (26).

$$k_{fQp}^{0,1} = k_{fQp}^{0,1}\left(\frac{1+[AA]/K_{AA}}{1+\beta_{AA}^{r}[AA]/K_{AA}}\right) \qquad \text{Eq. S57}$$

$$k_{fQp}^{1,2} = k_{fQp}^{1,2}\left(\frac{1+[AA]/K_{AA}}{1+\beta_{AA}^{r}[AA]/K_{AA}}\right) \qquad \text{Eq. S58}$$

$$k_{fQp}^{2,3} = k_{fQp}^{2,3}\left(\frac{1+[AA]/K_{AA}}{1+\beta_{AA}^{r}[AA]/K_{AA}}\right) \qquad \text{Eq. S59}$$

$$k_{fQp}^{3,4} = k_{fQp}^{3,4}\left(\frac{1+[AA]/K_{AA}}{1+\beta_{AA}^{r}[AA]/K_{AA}}\right) \qquad \text{Eq. S60}$$

$$k_{fQp}^{4,5} = k_{fQp}^{4,5}\left(\frac{1+[AA]/K_{AA}}{1+\beta_{AA}^{f}[AA]/K_{AA}}\right) \qquad \text{Eq. S61}$$

*Kinetic fits.* Model simulations of the kinetic data is shown below. When no uncertainty was given, we assumed an error model of 20% the maximum for a given data set.

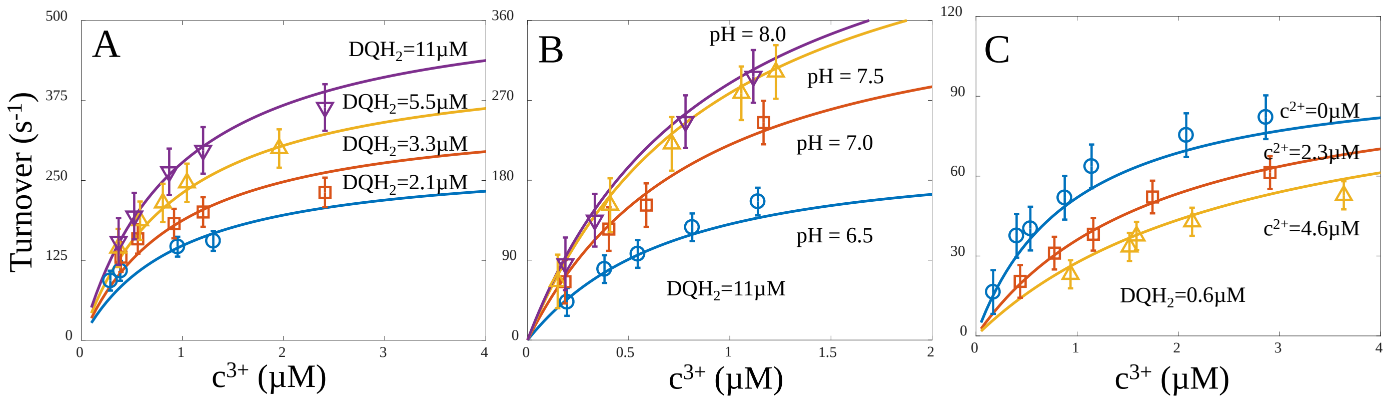


**Figure S5. The turnover rate of purified bc$_1$ complex oxidizing the Q-analogue decylubiquinol (DQH$_2$).** (A) The simulated rate of cyt c reduction versus c$^{3+}$ concentration at various fixed DQH$_2$ concentrations. (B) The simulated rate of cyt c reduction versus c$^{3+}$ concentration at various fixed pH at a fixed DQH2 concentration. (C) The simulated rate of cyt c reduction versus c$^{3+}$ concentration at various fixed c$^{2+}$ concentrations at a fixed DQH$_2$ concentration. The symbols represent the experimental data and lines represent model simulations. Data from (17).

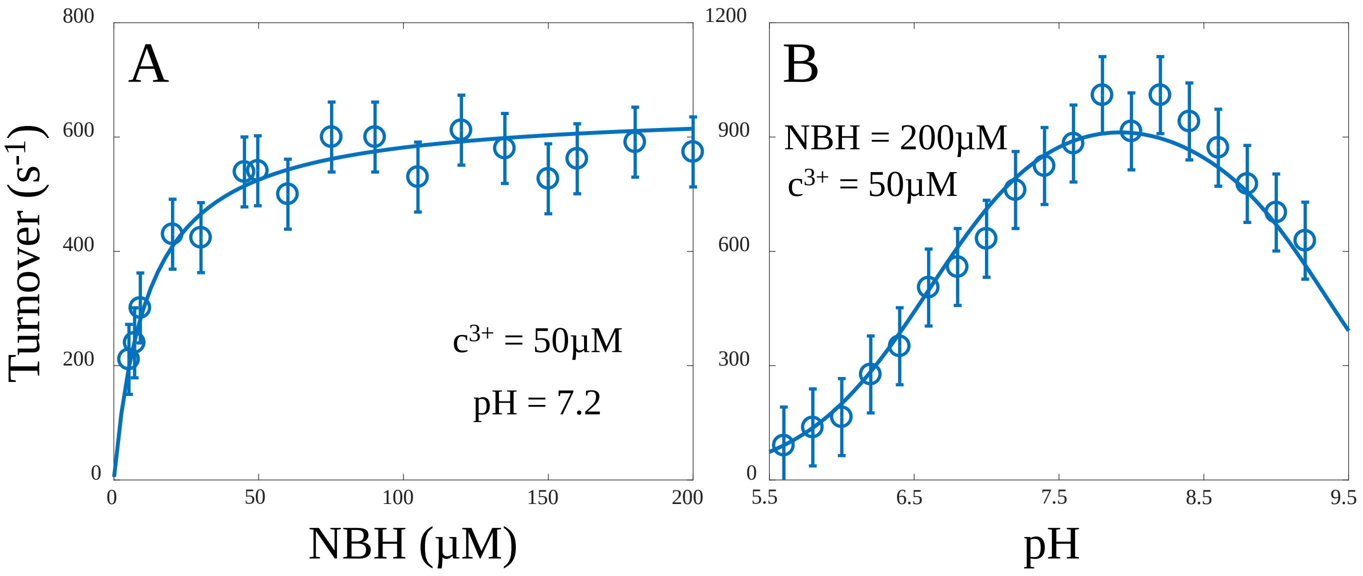


**Figure S6. The turnover rate of reconstituted bc$_1$ complex in proteoliposomes oxidizing the Q-analogue nonylubiquinol (NBH).** (A) The simulated turnover rate versus NBH concentration at fixed pH and cytochrome c$^{3+}$ concentration. (B) The simulated turnover rate versus pH at fixed cytochrome c$^{3+}$ and NBH concentrations. Data from (1).

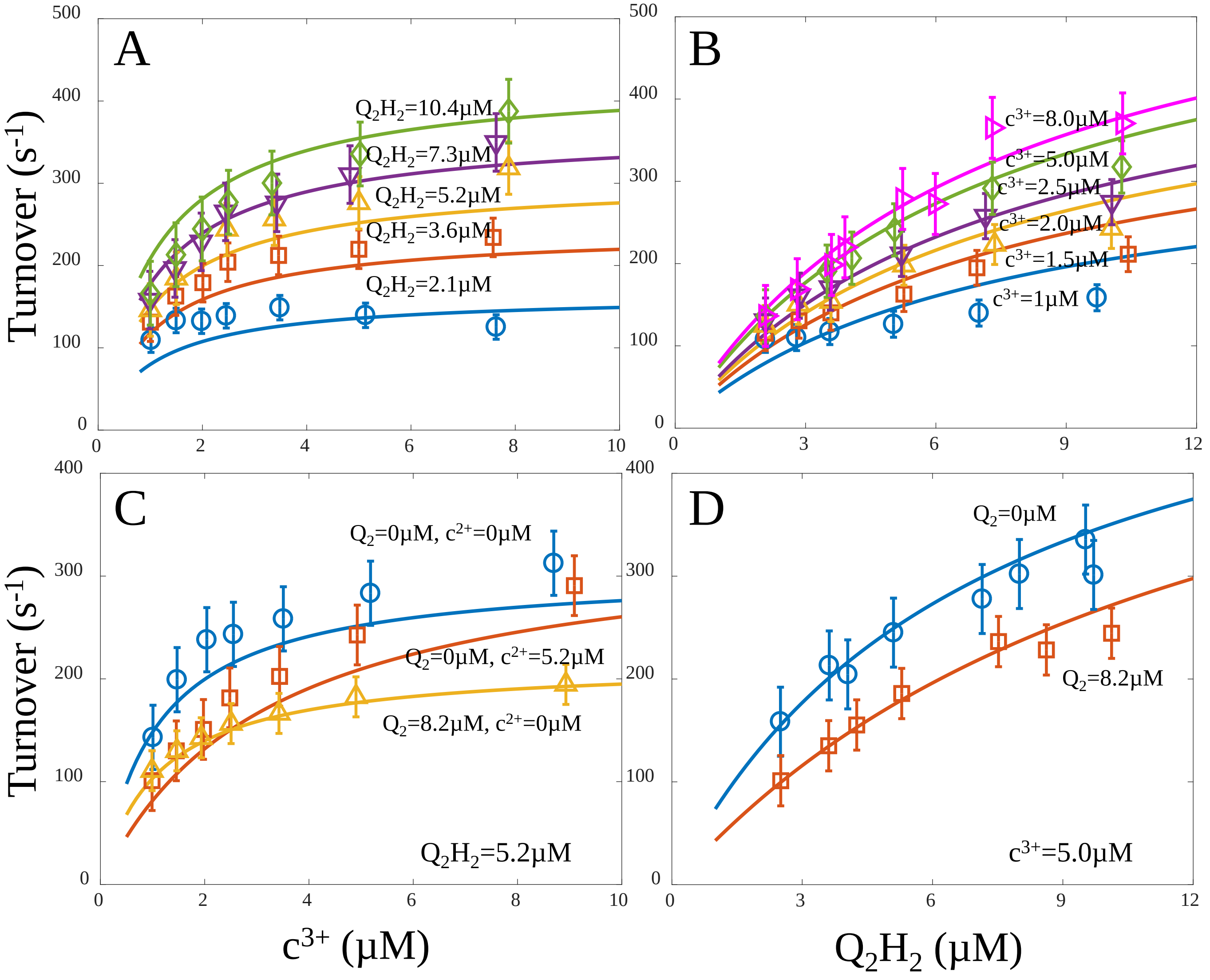


**Figure S7. The turnover rate of purified $bc_1$ complex oxidizing the Q-analogue ubiquinol-2 ($Q_2H_2$).** (A) The simulated turnover rate versus cytochrome $c^{3+}$ concentration at varying $Q_2H_2$ concentrations. (B) The simulated turnover rate versus $Q_2H_2$ concentration at varying cytochrome $c^{3+}$ concentrations. (C) The simulated turnover rate versus cytochrome $c^{3+}$ concentration at varying $Q_2$ and cytochrome $c^{2+}$ concentrations at a fixed $Q_2H_2$ concentration. (D) The simulated turnover rate versus $Q_2H_2$ concentration at varying $Q_2$ concentrations at a fixed cytochrome $c^{3+}$ concentration. Fixed concentrations for each simulation are indicated on each panel. The symbols represent the experimental data and lines represent model simulations. Data from (18).

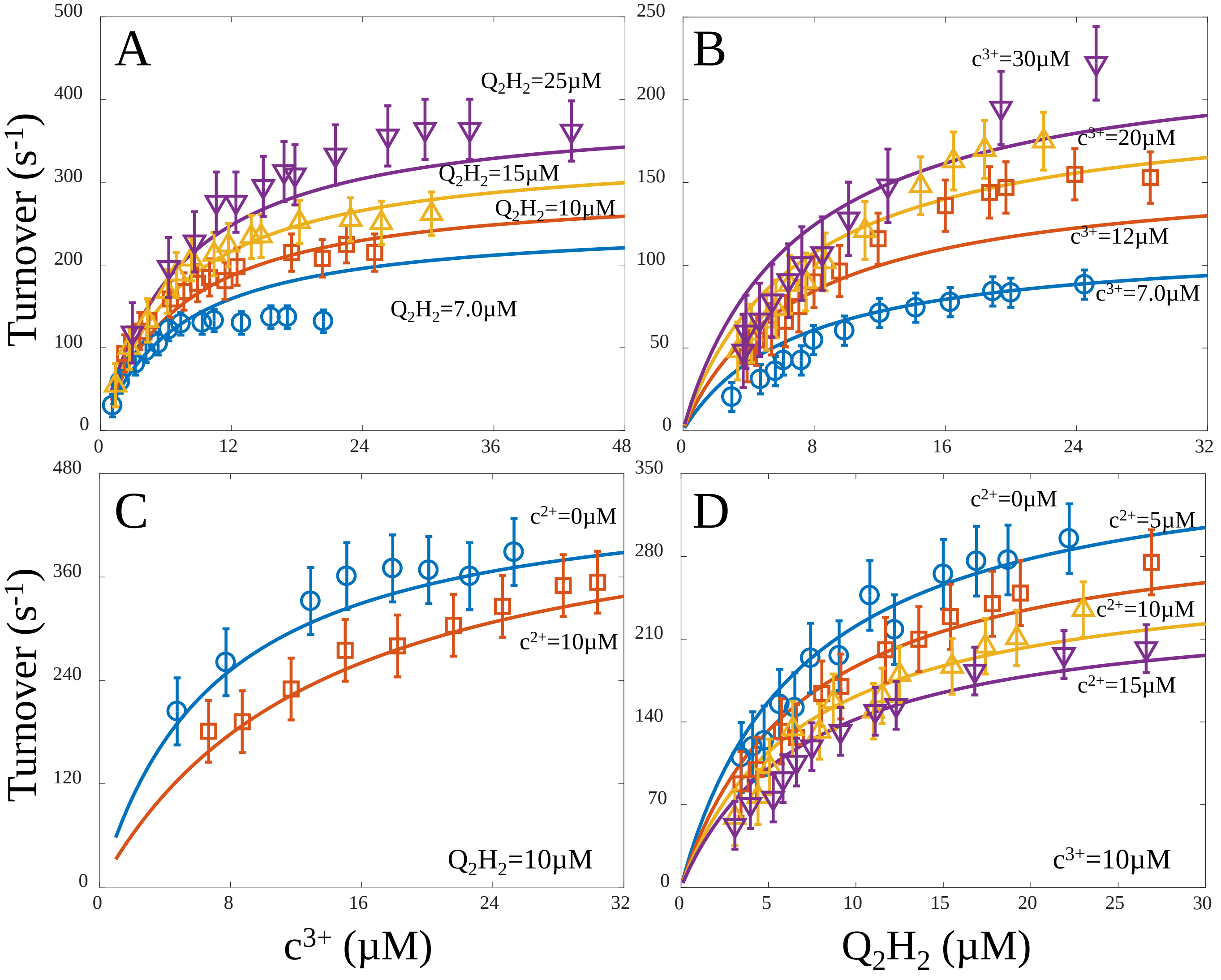


**Figure S8. The turnover rate of purified $bc_1$ complex oxidizing the Q-analogue ubiquinol-2 ($Q_2H_2$).** (A) The simulated turnover rate versus cytochrome $c^{3+}$ concentration at various fixed $Q_2H_2$ concentrations. (B) The simulated turnover rate versus $Q_2H_2$ concentration at various fixed cytochrome $c^{3+}$ concentrations. (C) The simulated turnover rate versus cytochrome $c^{3+}$ concentration at various fixed cytochrome $c^{2+}$ concentrations at a fixed $Q_2H_2$ concentration. (D) The simulated turnover rate versus $Q_2H_2$ concentration at various fixed cytochrome $c^{2+}$ concentrations at a fixed cytochrome $c^{3+}$ concentration. The symbols represent the experimental data and lines represent model simulations. Data from (19).

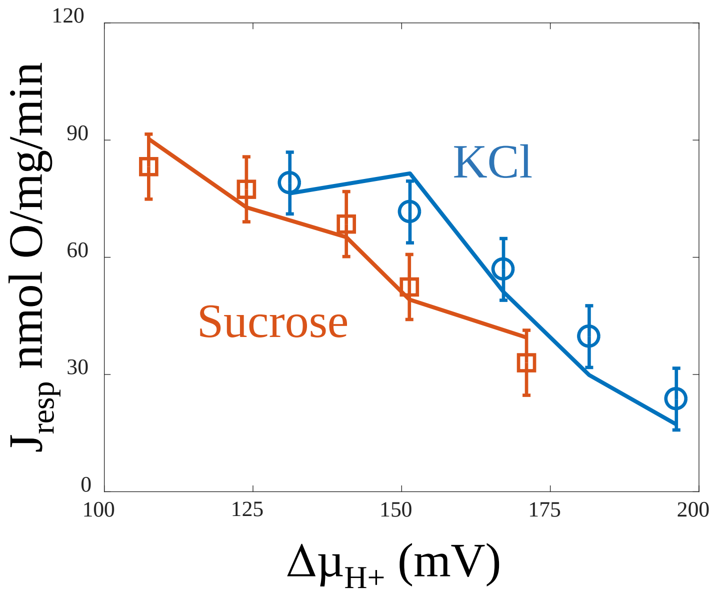


**Figure S9. The effect of a proton-motive force on turnover.** (A) The steady-state rate of oxygen consumption by isolated rat liver mitochondria in the presence of rotenone and oligomycin versus the proton-motive force is shown. The oxygen consumption rate is equivalent to two times the turnover rate of the $bc_1$ complex. The blue line and circles reflect the simulated turnover rate and data, respectively, when the mitochondria are suspended in a KCl-based respiration buffer. The orange line and circles show the simulated turnover rate and data, respectively, when the respiration buffer is switched to sucrose-based buffer. Exogenous $Q_2H_2$ and cytochrome c were supplied to the mitochondrial suspension. The proton-motive force is defined as: $\Delta\mu_{H+} = \Delta\Psi + 59\Delta pH$. Data from (20).

## Model Code Description

During the editorial process, each file in the zip was appended with "_V1." In order to run the code without issue, first remove "_V1" from each file.

bc1_code.zip – zip file containing the following files:

bc1_data.mat – mat file containing data structure

bc1_params.mat – mat file containing model parameters

bc1_dimer.m – function containing dimer model code

Generate_Figures.m – function containing code to simulate the model and plot the figures

# Supporting References


1. Brandt, U., and J. G. Okun. 1997. Role of deprotonation events in ubihydroquinone:cytochrome c oxidoreductase from bovine heart and yeast mitochondria. Biochemistry 36:11234-11240.
2. Rich, P. R., A. E. Jeal, S. A. Madgwick, and A. J. Moody. 1990. Inhibitor effects on redox-linked protonations of the b haems of the mitochondrial bc1 complex. Biochimica et biophysica acta 1018:29-40.
3. Eddowes, M. J., and H. A. O. Hill. 1979. Electrochemistry of Horse Heart Cytochrome-C. J Am Chem Soc 101:4461-4464.
4. Crofts, A. R., V. P. Shinkarev, D. R. Kolling, and S. Hong. 2003. The modified Q-cycle explains the apparent mismatch between the kinetics of reduction of cytochromes c1 and bH in the bc1 complex. J Biol Chem 278:36191-36201.
5. Urban, P. F., and M. Klingenberg. 1969. On the redox potentials of ubiquinone and cytochrome b in the respiratory chain. Eur J Biochem 9:519-525.
6. Ohnishi, T., and B. L. Trumpower. 1980. Differential effects of antimycin on ubisemiquinone bound in different environments in isolated succinate . cytochrome c reductase complex. J Biol Chem 255:3278-3284.
7. Brandt, U. 2006. Energy converting NADH:quinone oxidoreductase (complex I). Annu Rev Biochem 75:69-92.
8. Glaser, E. G., S. W. Meinhardt, and A. R. Crofts. 1984. Reduction of cytochrome b-561 through the antimycin-sensitive site of the ubiquinol-cytochrome c2 oxidoreductase complex of Rhodopseudomonas sphaeroides. FEBS letters 178:336-342.
9. Glaser, E. G., and A. R. Crofts. 1984. A new electrogenic step in the ubiquinol:cytochrome c2 oxidoreductase complex of Rhodopseudomonas sphaeroides. Biochimica et biophysica acta 766:322-333.
10. Robertson, D. E., and P. L. Dutton. 1988. The nature and magnitude of the charge-separation reactions of ubiquinol cytochrome c2 oxidoreductase. Biochimica et biophysica acta 935:273-291.
11. Ding, H., C. C. Moser, D. E. Robertson, M. K. Tokito, F. Daldal, and P. L. Dutton. 1995. Ubiquinone pair in the Qo site central to the primary energy conversion reactions of cytochrome bc1 complex. Biochemistry 34:15979-15996.
12. Wikström, M., and Royal Society of Chemistry (Great Britain). 2005. Biophysical and structural aspects of bioenergetics. Royal Society of Chemistry, Cambridge, UK.
13. Lass, A., S. Agarwal, and R. S. Sohal. 1997. Mitochondrial ubiquinone homologues, superoxide radical generation, and longevity in different mammalian species. J Biol Chem 272:19199-19204.
14. Schwerzmann, K., L. M. Cruz-Orive, R. Eggman, A. Sanger, and E. R. Weibel. 1986. Molecular architecture of the inner membrane of mitochondria from rat liver: a combined biochemical and stereological study. J Cell Biol 102:97-103.
15. Murphy, M. P. 2009. How mitochondria produce reactive oxygen species. The Biochemical journal 417:1-13.
16. Kuo-chen, C., and J. Shou-ping. 1974. Studies on the rate of diffusion-controlled reactions of enzymes. Spatial factor and force field factor. Sci Sin 27:664-680.
17. Speck, S. H., and E. Margoliash. 1984. Characterization of the interaction of cytochrome c and mitochondrial ubiquinol-cytochrome c reductase. J Biol Chem 259:1064-1072.
18. Esposti, M. D., and G. Lenaz. 1991. The kinetic mechanism of ubiquinol: cytochrome c reductase at steady state. Archives of biochemistry and biophysics 289:303-312.
19. Kubota, T., S. Yoshikawa, and H. Matsubara. 1992. Kinetic mechanism of beef heart ubiquinol:cytochrome c oxidoreductase. Journal of biochemistry 111:91-98.
20. Brown, G. C., and M. D. Brand. 1985. Thermodynamic control of electron flux through mitochondrial cytochrome bc1 complex. The Biochemical journal 225:399-405.
21. Rottenberg, H., R. Covian, and B. L. Trumpower. 2009. Membrane potential greatly enhances superoxide generation by the cytochrome bc1 complex reconstituted into phospholipid vesicles. J Biol Chem 284:19203-19210.
22. Quinlan, C. L., A. A. Gerencser, J. R. Treberg, and M. D. Brand. 2011. The mechanism of superoxide production by the antimycin-inhibited mitochondrial Q-cycle. J Biol Chem 286:31361-31372.
23. Covian, R., E. B. Gutierrez-Cirlos, and B. L. Trumpower. 2004. Anti-cooperative oxidation of ubiquinol by the yeast cytochrome bc1 complex. J Biol Chem 279:15040-15049.

24. Drose, S., and U. Brandt. 2008. The mechanism of mitochondrial superoxide production by the cytochrome bc1 complex. J Biol Chem 283:21649-21654.
25. Squire, W., and G. Trapp. 1998. Using complex variables to estimate derivatives of real functions. Siam Rev 40:110-112.
26. Castellani, M., R. Covian, T. Kleinschroth, O. Anderka, B. Ludwig, and B. L. Trumpower. 2010. Direct demonstration of half-of-the-sites reactivity in the dimeric cytochrome bc1 complex: enzyme with one inactive monomer is fully active but unable to activate the second ubiquinol oxidation site in response to ligand binding at the ubiquinone reduction site. J Biol Chem 285:502-510.
