# Supplementary material for: Analysis of a Functional Dimer Model of Ubiquinol Cytochrome c Oxidoreductase
Source: Biophys J. 2017 Oct 3;113(7):1599–612. doi: 10.1016/j.bpj.2017.08.018 (PMC5627346; doi:10.1016/j.bpj.2017.08.018)
Supplement: Document S2. Article plus Supporting Material [file mmc3.pdf]

Biophysical Journal
Article

Biophysical Society

Analysis of a Functional Dimer Model of Ubiquinol Cytochrome *c* Oxidoreductase

Jason N. Bazil[1,*]
[1]Department of Physiology, Michigan State University, East Lansing, Michigan

ABSTRACT Ubiquinol cytochrome *c* oxidoreductase (bc_1 complex) serves as an important electron junction in many respiratory systems. It funnels electrons coming from NADH and ubiquinol to cytochrome *c*, but it is also capable of producing significant amounts of the free radical superoxide. In situ and in other experimental systems, the enzyme exists as a dimer. But until recently, it was believed to operate as a functional monomer. Here we show that a functional dimer model is capable of explaining both kinetic and superoxide production rate data. The model consists of six electronic states characterized by the number of electrons deposited on the complex. It is fully reversible and strictly adheres to the thermodynamics governing the reactions. A total of nine independent data sets were used to parameterize the model. To explain the data with a consistent set of parameters, it was necessary to incorporate intramonomer Coulombic effects between hemes b_L and b_H and intermonomer Coulombic effects between b_L hemes. The fitted repulsion energies fall within the theoretical range of electrostatic calculations. In addition, model analysis demonstrates that the *Q* pool is mostly oxidized under normal physiological operation but can switch to a more reduced state when reverse electron transport conditions are in place.

INTRODUCTION

Ubiquinol cytochrome *c* oxidoreductase (bc_1 complex) is an essential enzyme for all mammalian cells. This enzyme family catalyzes a proton-coupled redox reaction that accounts for a significant fraction of energy required to maintain the proton motive force across biological membranes (1). The proton motive force is converted to a chemical potential energy in the form of the ATP hydrolysis potential (2), which is used to drive nearly all cellular processes. The enzyme serves as a hub for biological electron flow where many upstream catabolic pathways converge on the ubiquinone or quinone (Q) pool (3,4). Quinones are lipophilic, mobile electron carrier analogs to the hydrophilic NADH molecule and cytochrome *c* hemeprotein. The complex catalyzes the exergonic two-electron oxidation of quinol to the one-electron reduction of cytochrome *c* while transferring two charge-equivalents across biological membranes. The net biochemical reaction is the oxidation of a quinol molecule, reduction of two cytochrome *c* hemeproteins, release of four chemical protons on the opposite side of charge transfer, and consumption of two matrix protons. The net biochemical reaction is depicted as

$$QH_2 + 2c^{3+} + 2H_n^+ \rightleftharpoons Q + 2c^{2+} + 4H_p^+. \quad (1)$$

The enzyme operates using a Q-cycle mechanism whereby electron flow is bifurcated. Quinol is oxidized at the Q_p site, located on positive (or outer/intermembrane space) side of the membrane. The first electron is passed down the high potential chain to reduce cytochrome *c* at this outer surface. The other is sent down the low potential chain consisting of *b*-type hemes to reduce a quinone to form a stable semiquinone at the Q_n site, located on the negative (or inner/matrix) side of the membrane. Another turnover (i.e., a second quinol oxidation at the Q_p site) generates a second reduced cytochrome *c* molecule and a fully reduced quinol at the Q_n site (matrix side). In total, two quinol molecules are oxidized to form two reduced cytochrome *c* molecules and regenerate a quinol molecule at the Q_n site.

In mitochondria, the bc_1 complex plays an important role in regulating energy transduction and free radical generation, but the precise mechanisms are still relatively obscure. Crystal structure data of the mammalian complex has helped to resolved many questions centered on the reaction mechanism. The first structures published showed the enzyme crystalized as a dimer and localized key catalytic components on the complex (5–7). Additional structures demonstrated that mobility of the iron sulfur protein (ISP) head domain was essential for catalytic function (8) and revealed

Submitted February 21, 2017, and accepted for publication August 10, 2017.

*Correspondence: jnbazil@msu.edu

Editor: Wendy Shaw.
http://dx.doi.org/10.1016/j.bpj.2017.08.018

© 2017 Biophysical Society.
This is an open access article under the CC BY license (http://creativecommons.org/licenses/by/4.0/).
CrossMark

some of the Q_n-site details (9,10). Structures from other organisms also helped shape the catalytic landscape (11–13). More details covering structural analysis of the bc_1 complex and the Q-cycle mechanism are covered elsewhere (14,15).

Although the Q-cycle mechanism is generally well accepted (16), the details of all of the biochemical reactions occurring on the bc_1 complex are not. For example, the existence of a semiquinone during quinol oxidation at the Q_p site has been disputed for many years. Some groups postulated a mechanism involving a concerted two-electron oxidation (17–19). Other groups have provided evidence for the existence of a fleeting semiquinone serving as a reaction intermediary during the catalytic reactions of quinol between the Rieske ISP and heme b_L (20,21). In favor of this hypothesis, Cape et al. (22) and Vennam et al. (23) have shown that this semiquinone does indeed exist, albeit only in conditions that maximize the likelihood of finding it. Other studies have also reported finding a semiquinone intermediary at the Q_p site (24,25), but as pointed out by Pietras et al. (26), this matter is far from being resolved. In addition, the reaction at the Q_n-site has also been the subject of some controversy. Mulkidjanian (27) argues in favor of an activated Q-cycle mechanism whereby quinol oxidation at the Q_n site primes the enzyme for catalysis at the Q_p site. The mode of free radical production is also not settled. Some experiments clearly show an increase in the rate of free radical production with membrane potential (28,29), whereas others show a decrease in the presence of a membrane potential (30). Furthermore, the ability of the bc_1 complex to operate as a functional dimer has been a point of contention. Three groups have produced compelling evidence to suggest intermonomer electron transfer occurs on a sufficiently rapid timescale to support the dimer mechanism (18,31–35), whereas another calls into question the interpretation of those experiments (36,37). However, there is now convincing evidence of dimeric function in vivo (38), although the effect of dimeric function on enzyme kinetics and free radical generation has not been fully explored.

Although other mathematical models of the bc_1 complex exist (30,39–51), they are too complex to integrate into larger-scale metabolic models (47–49), do not include free radical production (39–46,48), are not thermodynamically constrained (30,42–46,50,51), or simulate a limited range of conditions (30,41–46,48). A steady-state model capable of simulating both catalytic activity and important side reactions is an ideal choice for integration into next-generation mitochondrial bioenergetics models. Here we present a steady-state model of the kinetics of the bc_1 complex capable of simultaneously simulating the rate of free radical production over a range of conditions. In addition, the method employed is generalized from our prior models (52,53) and easily adaptable to other enzyme-mediated processes.

The model is used to test whether or not the Q cycle is sufficient to explain the available data, the mechanism of free radical production, and the feasibility of dimer operation. The model assumes that electron input at the Q_p and Q_n sites is independent, and that electrons are free to distribute themselves across either monomer to settle in the lowest energy state. We previously showed that a simple monomer model was sufficient to explain a majority of the kinetic data (53). However, simulating free radical production in addition to the available kinetic data requires a dimer model.

METHODS

Generalized constitutive model equations

The model presented herein is more biophysically consistent with the known mechanism and crystal structure than our previous model of the bc_1 complex (53), and the modeling approach is more similar to our Complex I model (52). An overall model scheme is presented in Fig. 1. The model is constructed using mass action kinetics and assumes rapid binding and unbinding of substrates and products. The model equations strictly adhere to the thermodynamics governing the reaction at each elementary step in the catalytic cycle. The oxidation and reduction reactions occur when the enzyme is in the appropriate enzyme-substrate complex. The enzyme-substrate complexes are computed using binding polynomials. Briefly, electrons are added to the complex via ubiquinol (QH_2) oxidation in a two-electron step (32) at the Q_p site and removed via Q reduction at the Q_n site and cytochrome c reduction at the cytochrome c_1 site. For simplicity, we lump QH_2 oxidation at the Q_p site with cytochrome c reduction at the c_1 site, so electrons enter the bc_1 complex one at a time at the Q_p site. Electrons can also be removed via a secondary mechanism by molecular oxygen at the Q_p site to form superoxide:

$$QH_2 + c^{3+} + O_2 \underset{}{\overset{\text{at } Q_p \text{ site}}{\rightleftharpoons}} Q + c^{2+} + O_2^{-} + 2H_P^{+}. \quad (2)$$

In addition, the mechanism is fully reversible. So, for example, in reverse electron transport, electrons can be added via QH_2 oxidation at the Q_n site and removed by Q reduction at the Q_p site. The model is based on a functional dimer mechanism (31) with each monomer containing four redox centers. We assume the Rieske ISP and cytochrome c_1 redox kinetics are not rate limiting under normal turnover conditions. This assumption is supported by experimental evidence showing QH_2 oxidation at the Q_p site is the rate-limiting step (21). We also include the effect of pH on electron transport down the high potential chain. Accordingly, the four redox centers per monomer that influence the overall reaction rate are a semiquinone at the Q_p site, a heme b_L, a heme b_H, and a semiquinone at the Q_n site. In the dimer, the electrons equilibrate on the complex through the heme b_L-b_L electronic bus bar (i.e., electron bridge) (35). In addition, electron mobility is restricted by Coulombic repulsion interactions (54). With eight redox centers in the dimer model, the theoretical maximum number of electrons on the complex is eight. However, model simulations reveal an extremely low occupancy of states past five electrons. In addition, the minimum number of states necessary to simulate the antimycin A-inhibited complex is six. Therefore, only six electronic states are considered (oxidized plus up to the five-electron state).

The fractional substates of these redox centers for each electronic state are computed using the Boltzmann distribution:

$$s_r^k = \frac{e^{-\Delta G_r^k / RT}}{\sum_r e^{-\Delta G_r^k / RT}}, \quad (3)$$

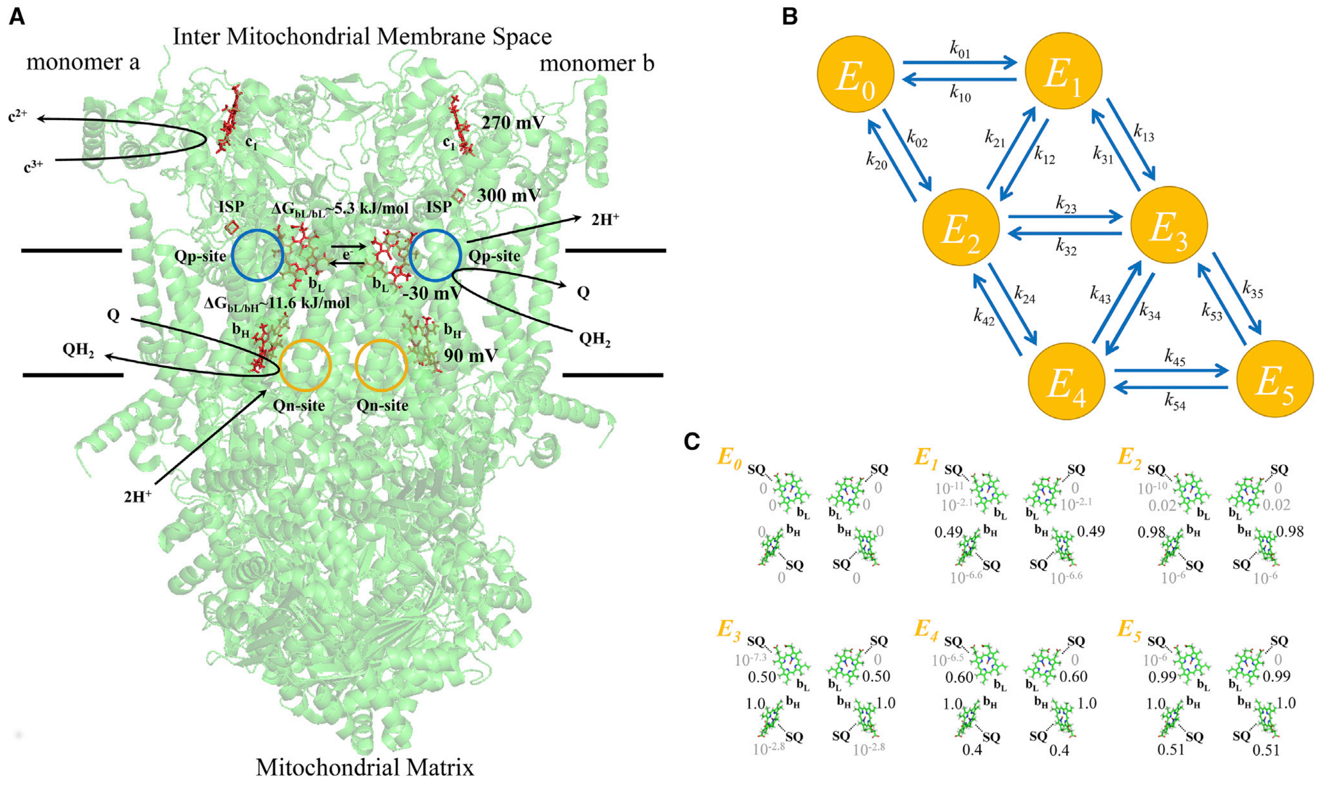

FIGURE 1 Model diagram of the bc_1 dimer. (*A*) Dimeric model of the bc_1 complex is shown with major redox centers and partial reactions. Midpoint potentials for the redox centers on the complex are from Table S1 and represented at pH 7. The model assumes only one Q_p site is active per turnover (31). Two turnovers at the Q_p site are required per turnover at the Q_n site. The model lumps oxidation of QH_2 at the Q_p site into a two-electron step. The first electron is used to reduce cytochrome *c*, and the second electron is deposited on the bc_1 complex. The monomer order during QH_2 oxidation at the Q_p site is random. Quinone reduction at the Q_n site is also random. Electrons on the complex distribute themselves among the redox centers according to the Boltzmann distribution. Electron transport between *b* hemes and the Q_n site is electrogenic. Coulombic interaction energies between intramonomer b_L and b_H hemes and intermonomer b_L and b_L hemes are included. Blue and yellow circles are approximate locations of Q_p- and Q_n-site binding pockets, respectively. The dimer cartoon was generated from the crystal structure by Esser et al. (13) (PBD: 5KLV). The depicted proton uptake and release pathways are only for visual purposes. (*B*) State representation of the model is shown where E_i is the *i*th electronic state corresponding to the number of electrons residing on the complex. The state-transition rate constants, k_{ij}, are given in the Supporting Material. Probabilities <0.4 are shown in gray. (*C*) For each enzyme state, E_i, the probability of finding an electron on each redox center in the model is shown for the following conditions: Q pool 10% reduced, membrane potential of 0 mV, pH 7 on both sides of the membrane, no cytochrome *c* present, and under anoxia. Cartoon rendering of the bc_1 complex was done using the software PyMOL (108). To see this figure in color, go online.

where s^k_r is the fraction of that redox center(s) *r* existing in electronic state *k* that is reduced, and ΔG_r^k is the standard free energy change for center(s) *r*. An example of the substate fractions given by the model under specific conditions is given in Fig. 1 *C*. For details of the conditions, see the figure legend. In Eq. 3, the redox center(s) *r* can consist of a single redox center or any combination of centers on the dimer. To compute the standard free energy change for combinations of redox centers, we assume additivity of the individual redox center standard free energies. The number of combinations of reduced redox centers for each state is given by the binomial coefficient where *n* is the number of redox centers and *k* is the number of electrons on the complex. The free energy change calculations for each redox center used in the model are given in Eqs. S14–S24 in the Supporting Material. Coulombic electrostatic energies are included in the dimer for the b_H/b_L monomeric and b_L/b_L dimeric interactions.

The remaining constitutive model equations are given in the Supporting Material, but a brief description of the model follows. To compute the steady-state turnover of the bc_1 complex for a given set of conditions (substrate and product concentrations, pH, membrane potential, etc.), we first need to solve the linear system of equations describing the steady-state relationship between the electronic states (E_i) considered in the model (see Eq. S25 in the Supporting Material). These equations are graphically depicted in Fig. 1 *B*. The edges connecting the electronic states represent partial reactions that govern how state *i* is connected to state *j*. These partial reactions encompass molecular processes such as QH_2 oxidation at the Q_p site, Q reduction at the Q_n site, and superoxide formation at the Q_p site. The equations for these partial reactions are given in Eqs. S26–S43 in the Supporting Material. When state transitions occur (e.g., $E_i \rightarrow E_j$), the enzyme needs to be in the appropriate enzyme-substrate complex and substate form. For example, before Q_p-site catalysis can occur, the enzyme needs to be bound with QH_2 and ferricytochrome *c* (c^{3+}). For QH_2 to bind to the complex, the Q_p site needs to be unoccupied. Likewise, the c_1 site needs to be unoccupied before c^{3+} can bind. (Recall that we lump QH_2 oxidation and c^{3+} reduction into a single step for simplicity.) As another example, before QH_2 can form and dissociate from the complex at the Q_n site, the enzyme needs to be in the appropriate substate form. This requires a semiquinone (SQ) bound at the Q_n site with a reduced b_H heme on the same monomer. The fraction during which a given electronic state exists for this substate is computed using Eq. 3. With a steady-state solution for the electronic states known, the steady-state turnover rates for cytochrome *c* reduction, superoxide production, QH_2 oxidation, and Q reduction can be computed using Eqs. S44–S54 in the Supporting Material. For more details, we refer the reader to the Supporting Material.

Experimental data

To calibrate the model, we used a wide variety of data from the literature. These data consist of kinetic data (41,43,44,55,56) in addition to data on superoxide production rates (28,57) and dimeric function (31). The superoxide data consist of rich data sets that report the rate of superoxide production as a function of membrane potential and the effect of antimycin A on both cytochrome *c* reduction and superoxide production. We also included additional data to constrain the maximum rate of antimycin A-stimulated free radical production (58) in mammalian mitochondria. The monomer model cannot simultaneously fit these data with a single parameter set; thus, a new dimer model is necessary to explain the superoxide production and antimycin A-stimulation data. For more details about the data sets used for parameter estimation, see Table S2.

In many experiments used to parameterize the model, the Q analogs exhibited a nonenzymatic reaction with cytochrome *c* that is strongly pH dependent (59,60). In most articles, the authors indicated that this oxidation rate was subtracted from the measured rate of cytochrome *c* reduction. Unfortunately, the rates were not reported in many of the studies, nor did the experimental methods give enough details to allow for this phenomenon to be accurately modeled. Therefore, we had to estimate the extent of oxidation for each data set with an adjustable parameter (see Table 1). This parameter is a measure of how much of the reduced Q analog was oxidized before the rate of the enzymatic reaction was recorded.

Model code

The model was developed, parameterized, and simulated on a Dell Precision T5810 workstation (Round Rock, TX) with an Intel Xeon CPU E5-2640 v3 (Santa Clara, CA) at 2.6 GHz and 32 GB RAM using the software MATLAB (v. 2016a; The MathWorks, Natick, MA). The steady-state equation for the six-state model was solved analytically using MATLAB's symbolic toolbox. A custom, parallelized simulated annealing algorithm was used to globally search the parameter space before identifying a local minimum with a gradient-based local optimizer. Model code is given in the Supporting Material.

RESULTS AND DISCUSSION

Fitted model parameters

The model adjustable parameters are listed in Table 1. The model parameters were identified by simultaneously fitting all the kinetic and superoxide data with a single consistent set of parameters. The dissociation constants for the various Q analogs are highly correlated and not reliably identifiable using only the kinetic data. Therefore, we opted to constrain these parameters by assuming the following: 1) the dissociation constants at the Q_p site are similar to each other and within an order of magnitude (61,62); 2) the dissociation constant for QH_2 at the Q_n site is equal to or up to two orders-of-magnitude smaller than the Q_p site dissociation constants (63); and 3) the dissociation constant for Q at the Q_p site is equal to or up to two orders-of-magnitude higher than the dissociation constant for QH_2 at the Q_n site (63,64). We emphasize that these dissociation constants are apparent dissociation constants in that they are taken with respect to the aqueous phase. To identify the actual dissociation constants, we would need to know the partition coefficients for all the Q analogs for the various organic phases used in the experiments. In addition, the rate constants for the reactions at the Q_p- and Q_n sites were constrained by adding a difference penalty to the cost function; this difference penalty keeps the Q_p- and Q_n-site rate constants similar to each other during parameter estimation. But even with this penalty applied, a few rate constants were required to be significantly different from the others. Specifically, the Q_p-site QH_2 oxidation rate constant for the four-electron reduced state was low compared to the other rate constants. Also, the Q_n-site Q-reduction rate constants for the four- and five-electron reduced states were lower than the other reductase rates constants. These differences were necessary to fit the data. For the former parameter, a low rate constant was required to prevent an unrealistic amount of superoxide production in the antimycin A-inhibited state. For the latter parameter, a lower rate constant was necessary to improve fits to the kinetic data sets collected under energized conditions.

The fits to the kinetic data used to calibrate the model are shown in Figs. S5–S9. These results show that the model is capable of recapitulating the observed cytochrome *c* reduction kinetics under a wide variety of experimental conditions. The effect of product inhibition, pH, and energization state are captured well by the model simulations. Unfortunately, these data were collected using hydrophilic Q analogs, so the dissociation constants obtained from model fitting are not able to be used to simulate bc_1 kinetics under conditions with the native substrate, Q_{10}. However, the rate constants fit by the model can be used with approximate Q_{10} dissociation constants to simulate bc_1 kinetics and superoxide production rates in its native state. See the Native Q_{10} Dissociation Constants and Physiological Q-Pool Operating Range and Cardiac bc_1 Content subsections below for details.

Sensitivity analysis identifies the top-10 ranked parameters that are associated with the internal energy states of the enzyme, the rate-limiting step in the catalytic cycle, the antimycin A-inhibition factors, four quinone and cytochrome *c* binding constants, and parameters related to the experimental design for two data sets. We should note that the sensitivity analysis is local and thus only reflects how changes in model parameters affect model outputs in the neighborhood of the optimal point in parameter space. In addition, the values given in Table 1 are averages of the nonzero local sensitivity coefficients computed from model outputs (cytochrome *c* reduction and superoxide production rates) coinciding with the data using Eqs. S55 and S56 in the Supporting Material, so they do not give a global perspective on how these parameters change the model outputs under experimental conditions not used for parameter estimation. The parameter correlation matrix heat map (Fig. S1) shows that the majority of parameters are relatively uncorrelated, with pockets of correlation centered on the specific Q-analog binding constants. In addition, some of the experimental design parameters are correlated with their respective Q-analog binding constants. Therefore,

TABLE 1 Model Adjustable Parameters

Parameter	Definition	Value	Units	Sensitivity	Rank
Rate Constants					
$kf_{Q_p}^{0,1}$	quinol oxidation rate for E_0	2.38E+03	s^{-1}	1.57E−03	37
$kf_{Q_p}^{1,2}$	quinol oxidation rate for E_1	1.26E+03	s^{-1}	5.52E−01	4
$kf_{Q_p}^{2,3}$	quinol oxidation rate for E_2	3.15E+03	s^{-1}	2.33E−01	16
$kf_{Q_p}^{3,4}$	quinol oxidation rate for E_3	2.27E+03	s^{-1}	4.31E−02	26
$kf_{Q_p}^{4,5}$	quinol oxidation rate for E_4	4.14E+00	s^{-1}	3.50E−02	28
β_{AA}^{r}	antimycin A inhibition factor for semireverse mode of superoxide production	3.44E+02	—	3.68E−01	9
β_{AA}^{f}	antimycin A inhibition factor for semiforward mode of superoxide production	5.00E+00	—	3.82E−01	8
$kf_{Q_n}^{2,0}$	quinone reduction rate for E_2	9.19E+09	s^{-1}	4.43E−05	39
$kf_{Q_n}^{3,1}$	quinone reduction rate for E_3	9.21E+09	s^{-1}	2.23E−02	32
$kf_{Q_n}^{4,2}$	quinone reduction rate for E_4	6.98E+03	s^{-1}	1.05E−01	22
$kf_{Q_n}^{5,3}$	quinone reduction rate for E_5	1.13E+04	s^{-1}	1.92E−02	33
Binding Constants					
$K_{c^{3+}}$	c^{3+} binding constant	1.11E−06	M	2.61E−01	14
$K_{c^{2+}}$	c^{2+} binding constant	2.49E−06	M	9.17E−02	23
$K_{c^{3+}}^{Mg^{2+}}$	c^{3+} binding constant in presence of excess Mg^{2+}	1.14E−05	M	4.74E−01	6
$K_{c^{2+}}^{Mg^{2+}}$	c^{2+} binding constant in presence of excess Mg^{2+}	1.15E−05	M	2.38E−01	15
$K_{DQH_2}^{Q_p}$	DQH_2 binding constant at Q_p site	2.76E−06	M	4.26E−01	7
$K_{DQ}^{Q_p}$	DQ binding constant at Q_p site	3.77E+00	M	5.95E−03	36
$K_{DQ}^{Q_n}$	DQ binding constant at Q_n site	1.00E+02	M	6.71E−06	41
$K_{DQH_2}^{Q_n}$	DQH_2 binding constant at Q_n site	1.00E+00	M	1.50E−01	18
$K_{Q_2H_2}^{Q_p}$	Q_2H_2 binding constant at Q_p site	7.29E−06	M	3.49E−01	11
$K_{Q_2}^{Q_p}$	Q_2 binding constant at Q_p site	1.57E+00	M	1.75E−02	34
$K_{Q_2}^{Q_n}$	Q_2 binding constant at Q_n site	2.24E+01	M	1.38E−05	40
$K_{Q_2H_2}^{Q_n}$	Q_2H_2 binding constant at Q_n site	1.00E+00	M	7.17E−02	24
$K_{NBH}^{Q_p}$	NBH binding constant at Q_p site	1.23E−05	M	1.17E−01	20
$K_{NB}^{Q_p}$	NB binding constant at Q_p site	1.00E+00	M	4.16E−02	27
$K_{NB}^{Q_n}$	NB binding constant at Q_n site	3.41E+00	M	3.95E−04	38
$K_{NBH}^{Q_n}$	NBH binding constant at Q_n site	2.42E−01	M	2.59E−02	30
$K_{QH_2 mix}^{Q_p}$	mixed $Q_2H_2/Q_{10}H_2$ binding constant at Q_p site	2.59E−08	M	8.63E−01	2
$K_{Q mix}^{Q_p}$	mixed Q_2/Q_{10} binding constant at Q_p site	1.00E+00	M	4.88E−02	25
$K_{Q\ mix}^{Q_n}$	mixed Q_2/Q_{10} binding constant at Q_n site	1.00E+00	M	2.13E−01	17
$K_{QH_2\ mix}^{Q_n}$	mixed $Q_2H_2/Q_{10}H_2$ binding constant at Q_n site	1.00E-02	M	8.62E−01	3
Thermodynamic					
$\Delta G_{b_H/b_L}^{Coulomb}$	b_H/b_L monomeric Coulombic interaction energy	11.6	kJ/mol	1.36E+00	1
$\Delta G_{b_L/b_L}^{Coulomb}$	b_L/b_L dimeric Coulombic interaction energy	5.3	kJ/mol	2.76E−01	13
K_S^{Q10}	Q_p site stability constant for Q_{10}	2.28E−15	—	1.17E−01	19
$K_S^{Q\ analog}$	Q_p site stability constant for Q analogs	9.34E−09	—	3.18E−02	29
Initial Q-Pool Redox State (% Oxidized) for Data Sets					
Speck and Margoliash (44)	percent Q pool is oxidized	1.53	%	1.16E−01	21
Brandt and Okun (55)	percent Q pool is oxidized	5.01	%	2.37E−02	31
Esposti and Lenaz (41)	percent Q pool is oxidized	2.30	%	1.20E−02	35
Kubota et al. (43)	percent Q pool is oxidized	0.32	%	2.79E−01	12
Rottenberg et al. (28)	percent Q pool is oxidized	0.06	%	4.79E−01	5
Covian and Trumpower (78)	percent Q pool is oxidized	2.59	%	3.56E−01	10

Local sensitivity coefficients are normalized and averaged using Eqs. S55 and S56 in the Supporting Material. c^{2+}, ferrocytochrome *c*; c^{3+}, ferricytochrome c; DQH_2, decylubiquinol; NBH, nonyl-ubihydroquinone; Q_2H_2, ubiquinol-2; $Q_{10}H_2$, ubiquonol-10.

these binding constants are not readily identifiable without additional experimental data. The high sensitivity for some of the dissociation constants given in Table 1 reflects the importance of that parameter to fit that particular dataset. The fixed model parameters were obtained from the literature and are listed in Table S1.

Superoxide production

The model simulations of the superoxide data from reconstituted bc_1 complex (28) are shown in Fig. 2. It is important to note that these experiments were done under specific conditions that enabled robust superoxide production from the complex. The model reproduces the cytochrome *c* reduction (Fig. 2 *A*) and corresponding superoxide production rates (Fig. 2 *B*) for various pharmacological manipulations. Under the control conditions, the phospholipid vesicles reconstituted with bc_1 complex exhibited moderate cytochrome *c* reduction rates with elevated superoxide production rates. This is due to the presence of a membrane potential and pH gradient across the membrane. With the addition of FCCP, both the membrane potential and pH gradient are abolished, and the rate of cytochrome *c* reduction significantly increases whereas the rate of superoxide production dramatically falls to the minimum level. When nigericin is present, the pH gradient is converted to a membrane potential that leads to more superoxide production with a negligible effect on cytochrome *c* reduction relative to the control. In the presence of valinomycin, the membrane potential is dissipated. This leads to a significant stimulation of cytochrome *c* reduction, and the superoxide production rate collapses to values similar to the FCCP condition. When antimycin A is added, superoxide production rates skyrocket to levels just above those seen when nigericin is present. The cytochrome *c* reduction rate falls to levels about twice that of the superoxide production. This is due to the bypass reaction whereby the first electron from QH_2 reduces cytochrome *c*, the second electron reduces oxygen to produce superoxide (as shown in Eq. 2), and then the superoxide reduces cytochrome *c*. In addition, the model recapitulates the exponential dependency on membrane potential for superoxide production rather well (Fig. 2 *C*). This exponential relationship has been previously demonstrated by other groups (65,66); however, the data set of Rottenberg et al. (28) is the only one to show superoxide production that is exclusively from the bc_1 complex.

Model simulations of superoxide production data from antimycin A-inhibited submitochondrial particles (57) and intact skeletal muscle mitochondria inhibited by antimycin A (30) are shown in Fig. 3. The model simulates maximum levels of superoxide production when the Q pool is approximately half-oxidized (Fig. 3 *A*). These simulations were run with the decylubiquinone-related parameters (Table 1) and closely match data from a study by Dröse and Brandt (57). In this study, submitochondrial particles were fueled with a mixture of decylubiquinol/decylubiquinone, and the rate of free radical production was measured. They found the rate of superoxide production peaked when the Q pool was ~30% oxidized, but the uncertainty in the data limits how precisely this value can be determined. The model simulates this phenomenon rather well, but it shows a peak closer to 40%. More data are necessary to accurately pinpoint the area where the peak of superoxide formation occurs. As shown in Fig. 3 *B*, the model simulates similar behavior when run with the native Q_{10} parameters (Table 1; Table S1). In this study, mitochondria were purified from skeletal muscle, and the bc_1 complex was inhibited with antimycin A. The Q-pool redox state was modulated by the addition of succinate at various concentrations. The model predicts a peak free radical production of ~4 nmol H_2O_2/mg/min (= 8 nmol superoxide/mg/min) when the Q pool is ~45% oxidized

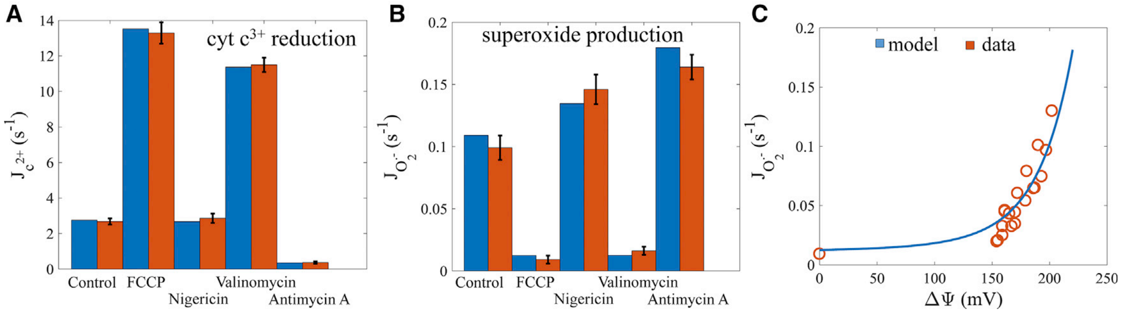

FIGURE 2 Cytochrome *c* turnover and superoxide production rate by the bc_1 complex reconstituted in liposomes. (*A*) Cytochrome *c* reduction rates are given for a variety of pharmacological manipulations. In the control case, the membrane potential and (2.3 RT/F)ΔpH values were 200 and 15 mV, respectively. With FCCP, both the membrane potential and ΔpH were set to zero. In the nigericin case, the ΔpH was set to 0 mV and the membrane potential was set to 215 mV. When valinomycin was present, the membrane potential was set to 0 mV with the ΔpH unchanged from the control. And when antimycin was present, the Q_n sites were inhibited. (*B*) Superoxide formation rates for the same pharmacological manipulations for the cytochrome *c* reduction rate data are shown. (*C*) The model simulates an exponentially increasing rate of superoxide production as the membrane potential is increased. This is caused by electron retention at the Q_p site of the complex, which leads to higher semiquinone occupancy. Data are from Rottenberg et al. (28). To see this figure in color, go online.

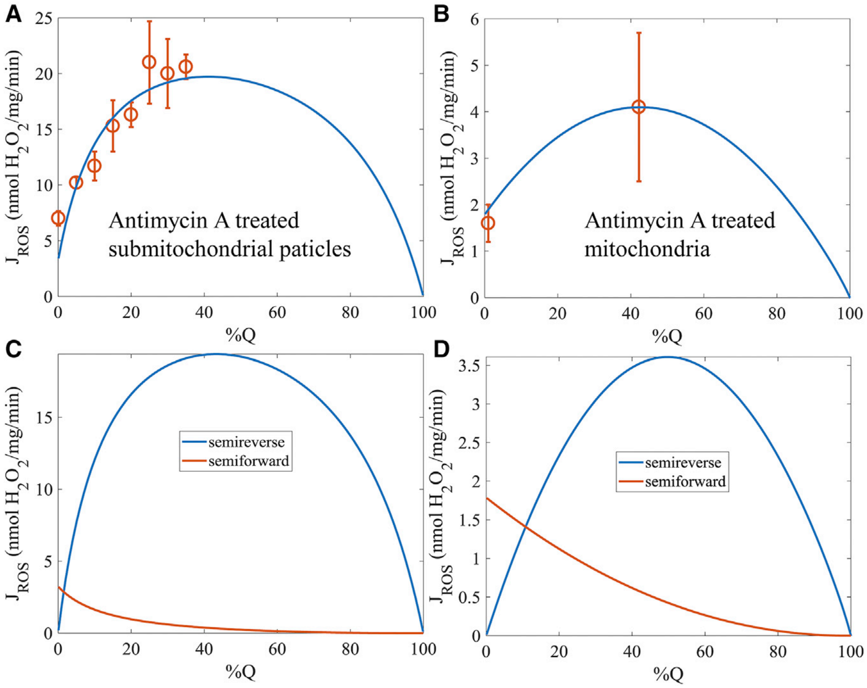

FIGURE 3 Free radical production rates by bc_1 complex in submitochondrial particles and intact mitochondria. (*A*) Model simulations of superoxide production by antimycin A-treated submitochondrial particles as a function of the Q-pool redox state using DQ kinetic constants as given in Table 1. (*B*) Model simulations of superoxide production by antimycin A-treated mitochondria as a function of the redox state of the Q pool using Q_{10} kinetic constants as given in Table S1. (*C*) The semiforward and semireverse rates of superoxide production for the conditions given in (*A*). (*D*) The semiforward and semireverse rates of superoxide production for the conditions given in (*B*). To convert the superoxide production rate to H_2O_2 production, we assumed a stoichiometric relationship of two superoxide molecules per H_2O_2. Data in (*A*) are from Dröse and Brandt (57). Data in (*B*) are from Quinlan et al. (30). To see this figure in color, go online.

(Fig. 3 *B*); when the Q pool is fully reduced, the rate is a little less than half of the maximum rate, near 1.5 nmol H_2O_2/mg/min. Unfortunately, in the study by Quinlan et al. (67), the Q-pool redox state is not known. This makes ascertaining the precise relationship between the Q-pool redox state and the superoxide production rate in this study impossible.

The superoxide production mechanism in the model includes both the semiforward and semireverse modes of superoxide production (26,68,69), as explained below. These results are given in Fig. 3, *C* and *D*. The dominant mechanism of superoxide production predicted by the model is the semireverse mode. This agrees well with the study by Sarewicz et al. (68). The semireverse mode of superoxide production occurs when the reduced heme b_L reduces Q at the Q_p site to form the unstable SQ that reacts with O_2 (26). In the model, this mode of superoxide production occurs when superoxide is formed from states E_1 through E_4. This mode of superoxide production shows a bell-curve-like relationship with the Q-pool redox state (Fig. 3, *C* and *D*). When the Q pool is highly reduced, the availability of Q to react with a reduced heme b_L is limited. When the Q pool is highly oxidized, the number of electrons on the complex is low, and they predominantly reside on the b_H hemes. The semiforward mode of superoxide production occurs when QH_2 is oxidized whereas heme b_L is reduced (26). This leaves an unstable SQ at the Q_p site that quickly reacts with oxygen to form superoxide. In the model, this mode of superoxide production occurs when superoxide is formed from state E_5. In this state, all available redox centers are reduced, leaving no other option than the formation of an SQ after QH_2 is oxidized at the Q_p site. This mode of superoxide production is highest when the Q pool is fully reduced. The rate monotonically decreases as the Q pool becomes more oxidized. This is due to a decrease in the state E_5 as the fraction of QH_2 declines.

To fit the superoxide production data for the antimycin A-inhibited complex, the QH_2 oxidation rates were lowered when antimycin was bound to the Q_n site. This modification is supported by prior studies (30,70–73). (For model details, see Eqs. S57–S61 in the Supporting Material.) Without this modification, the maximum rate of superoxide production occurred when the Q pool was 80–90% oxidized (data not shown). The exact mechanism leading to the slowdown of QH_2 oxidation at the Q_p site could be linked to the redox state of the *b* hemes (30,70), mobility of the Riekse ISP (71,72), and/or conformation changes in the protein structure (72,73), but there is evidence disputing such long-range interactions (74). In the model, a minimum of two factors was required to explain the data (see Table 1). The first inhibition factor lowered the rate of QH_2 oxidation for each state transition involved with the semireverse mode of superoxide production. A second inhibition factor was necessary for the semiforward mode of superoxide production.

Although this modification led to adequate fits to the available data, there are other possible mechanisms that can explain the data. Using the model, we can identify the critical processes that govern this phenomenon. The shape of the superoxide production rate profile as a function of the Q-pool redox state is determined by the net oxidation rates at the Q_p site. These net rates are determined by several conditions: 1) increased levels of reduced cytochrome *c*; 2) the Q and QH_2 dissociation constants at the Q_p site; 3) superoxide scavenging by Q; and 4) antimycin A-dependent

effects of the rate constants at the Q_p site, as discussed in the paragraph above. The model can be modified to include all these effects and still simulate the Q-stimulated superoxide production data from Dröse and Brandt (57). These factors are not mutually exclusive; however, we can rule out a few based on reasonable arguments and prior data. For condition 1, we found peak superoxide production rates near a 30% oxidized Q pool when cytochrome *c* was only 5% (57) or 20% (67) reduced. But it is unlikely that these levels of reduced cytochrome *c* are produced in either study. In the presence of antimycin A, cytochrome *c* oxidase keeps cytochrome *c* completely oxidized. Regarding condition 2, lowering the dissociation constant for Q by a factor of ~10 at the Q_p site can also lead to good fits to the data; however, this is not supported by the available data (62,75). For condition 3, superoxide scavenging by Q can explain the data (76). To include this mechanism, many additional parameters and assumptions are required to capture all the necessary details to properly model this phenomenon. Of all the potential mechanisms discussed, only condition 4 allowed for adequate fits to the data with the fewest additional parameters and changes to the model.

Antimycin A stimulation of cytochrome *c* reduction

The ability of antimycin A to stimulate cytochrome *c* reduction by the bc_1 complex is compelling evidence for cross-monomer electron equilibration (31). Mutagenic studies have led to this theory being nearly universally accepted (33,38). In this specific case, antimycin A concentrations are extremely low, and a significant fraction of dimers are bound with only a single inhibitor molecule. At saturating concentrations, antimycin A binds tightly to all Q_n sites, and electron flux through the enzyme only occurs at the Q_p site, via bypass reactions forming superoxide (see Eq. 2) (77). However, when only a single antimycin A molecule is bound to the dimer, cytochrome *c* reduction is stimulated. Fig. 4 *A* shows the model simulations of this phenomenon. This is only possible if the enzyme operates as a functional dimer with electron equilibration between monomers. Covian et al. (31) suggest that their data reveal only one operational Q_p site per turnover when both Q_n sites of the dimer are either bound with antimycin A or not. But when a single antimycin A molecule is bound to the dimer at one of the Q_n sites, both Q_p sites become operational and the rate of cytochrome *c* reduction is increased by a factor of 2. Our model simulations corroborate this hypothesis; however, we find that this condition leads to a 1.5-fold increase of superoxide production by a single bc_1 dimer. This leads to an overall increase in the maximal rate of cytochrome *c* reduction by a factor of 1.15 in the heterogeneously inhibited population of bc_1 complexes. In their original analysis, Covian et al. (31) did not appear to assume the Q_n sites bind antimycin A independently as we have done here. Fig. 4 *B* shows the enzyme fraction without antimycin A bound (*blue*), with a single antimycin A molecule bound to the dimer (*orange*), and with two molecules of the inhibitor bound (*yellow*) as a function of the number of antimycin A molecules per monomer for the simulations shown in Fig. 4 *A*.

Coulombic interactions, bc_1 kinetics, and free radical generation

Coulombic interactions (electron repulsive forces) in the bc_1 complex have been suggested to be important for controlling electron slippage (57), regulating enzyme turnover (54), and facilitating dimer operation (78). Of all the potential sites for Coulombic interactions in the bc_1 complex, we found two major sites necessary to fit the data. These sites are located on the intramonomer low potential chain (b_H and b_L) and the intermonomer dimeric interface (b_L and b_L). Of these two sites, the intramonomer interaction was most important with a fitted interaction energy of 11.6 kJ/mol. This is equivalent to an electrostatic interaction of ~−120 mV and well within the expected value assuming a protein dielectric constant in the range of 4–30 (21,79) and an intramonomer distance of 20.7 Å between hemes b_L and b_H (80). For the intermonomer Coulombic interaction energy, a value of 5.3 kJ/mol was determined to best fit the data and corresponds to an electrostatic interaction of ~−55 mV. Taking the intermonomer b_L distance as 20.9 Å (80) and same range of dielectric constants

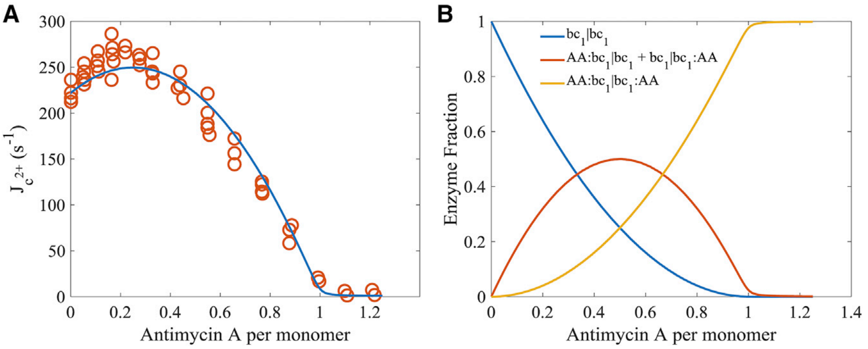

FIGURE 4 Antimycin-stimulated cytochrome *c* reduction. (*A*) At low antimycin A/monomer ratios (0–0.2), the rate of cytochrome *c* reduction is increased because both Q_p sites are active in the bc_1 dimers bound with a single antimycin A molecule. (*B*) As antimycin A is titrated, the fraction of antimycin A bound per monomer increases and follows the binding curves shown. We assumed antimycin A binds independently to either monomer. Data are from Covian et al. (31). To see this figure in color, go online.

mentioned above, this value also falls into a theoretical span of possible interaction potentials. These Coulombic repulsive forces bias electron distribution to favor a reduced b_H-SQ pair on one monomer relative to the condition in the absence of these repulsive forces. In this state, bc_1 complex turnover is most efficient and not accompanied with significant superoxide production. In the absence of these repulsive forces, the probability of finding an SQ on the complex drops two and four orders of magnitude for states E_3 and E_4, respectively (data not shown). We found that limiting electron mobility on the complex using these Coulombic interactions was required to fit the data sets that included energized mitochondria (56), free radical production (28,30,57), and antimycin A (28,30,57) with the kinetic data (41,43,44,55) using a single consistent parameter set.

Native Q_{10} dissociation constants

Unfortunately, kinetics using the native Q_{10} binding constants for the bc_1 complex are unavailable. So, identifying the Q_{10} binding parameters is more difficult than the Q-analog binding constants for several reasons. First, there is no kinetic data available for the native substrate; there is also no reliable method to quantify the redox state of the Q pool without destruction of the sample. This makes direct quantification impossible. Second, these estimates are based on thermodynamic and structural assumptions that may not hold for the native substrate during steady-state turnover of the enzyme (61–63). However, there are data we can use as a basis for developing likely in situ binding constants. For the Q_p site, Ding et al. (62) used kinetic and thermodynamic arguments to conclude dissociation constants for the Q_p site of QH_2 and Q are near equal and in the low millimolar range. For the Q_n site, Wikström (63) derives values for the both QH_2 and Q binding constants with QH_2 binding ~100-fold tighter than Q. Using these results, we can derive a set of values that leads to tenable cytochrome *c* reduction and superoxide production rates shown in Fig. 5. The bell-shaped relationship between the cytochrome *c* reduction rate and Q-pool redox state (Fig. 5 *C*, *upper plot*) and the exponential dependency of superoxide production rate on membrane potential (Fig. 5 *C*, *middle plot*) is similar to our prior simulations (53). In addition, the so-called bistability phenomenon (49,53) is still present (Fig. 5 *C*, *lower plot*); however, the relationship is qualitatively different. This phenomenon is characterized by the possibility of

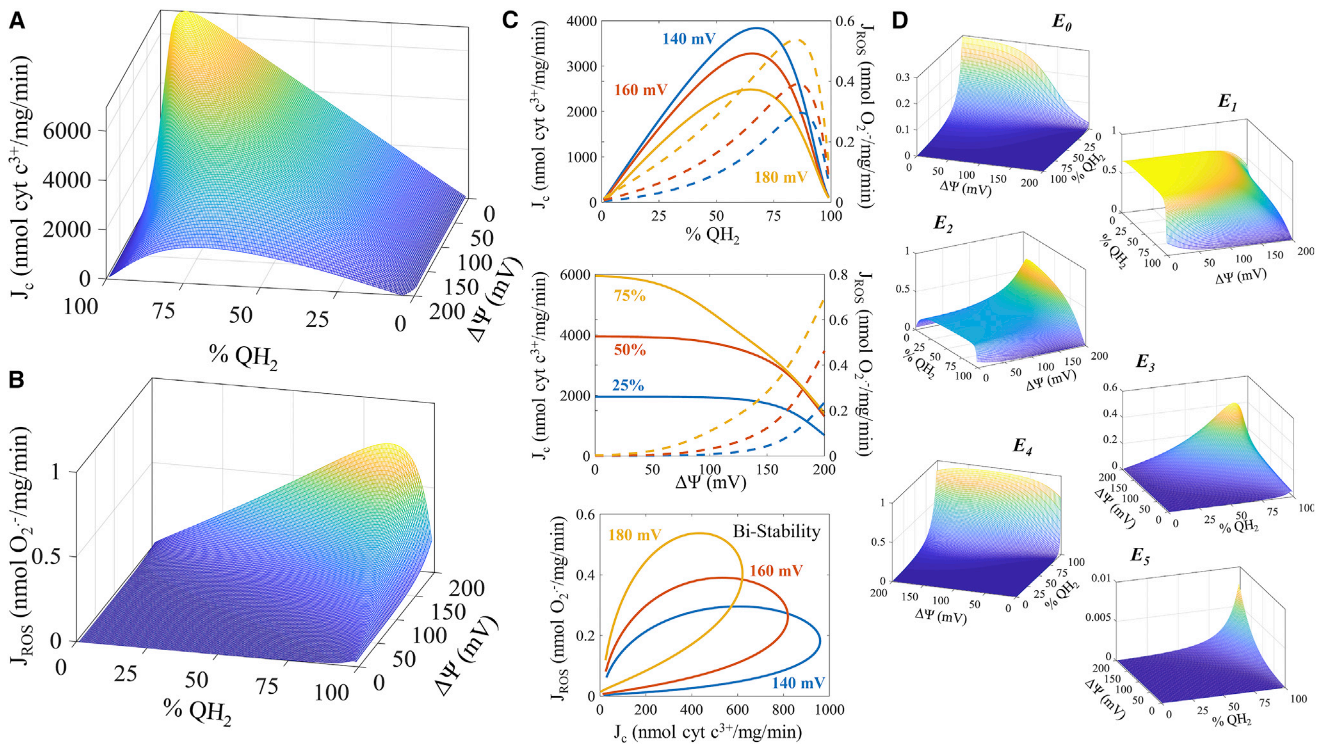

FIGURE 5 Physiological behavior of bc_1 dimer. For all the simulations, the cytochrome *c* pool was 20% reduced, matrix and cytosolic pH was 7, superoxide concentration was 100 pM, and oxygen was 30 μM. The Q pool was 20 mM and the reduced fraction and the membrane potential were variable as shown. (*A*) The cytochrome *c* reduction rates show a maximum rate when the Q pool is nearly fully reduced (~90%) at 0 mV, which shifts to a maximum when the pool is ~65% reduced at high membrane potentials. (*B*) The superoxide production simulations indicate a maximum when the Q pool is 75% reduced that shifts to ~95% as the membrane potential approaches zero. (*C*) Slices through the surface of cytochrome *c* reduction rate (*solid lines*) and superoxide production rate (*dashed lines*) at various Q-pool redox states and membrane potentials are shown in the top two panels. In the bottom panel, the bistability stability phenomenon is shown. (*D*) Enzyme state occupancies for these conditions reveal that higher reduced states are achieved as both the Q pool becomes reduced and the membrane potential is high. To see this figure in color, go online.

two free radical production rates at a given rate of cytochrome c reduction. But these simulations reveal that bistability also occurs at a given rate of superoxide production. This latter phenomenon is due to a significant drop in superoxide production under extremely reduced Q-pool redox states that may help minimize free radical production under these conditions. Some other notable differences between our previous simulations and the current ones are the overall shapes of the cytochrome c reduction and superoxide production rates as a function of membrane potential and Q-pool redox state, maximum rates of superoxide production, and the enzyme distribution surfaces (Fig. 5 *D*). For cytochrome c reduction, the maximum rate occurs when the Q pool is 90% reduced and the membrane potential is low (Fig. 5 *A*). But as the membrane potential increases, the maximum rate of cytochrome c reduction occurs when the Q-pool redox state is closer to 65% reduced (Fig. 5, *A* and *C*, *upper plot*). The superoxide production rate is highest when the Q pool is mostly reduced (75–95%) for nearly all membrane potentials (Fig. 5, *B* and *C*, *upper plot*). The enzyme state distributions show varying levels of occupancy depending on the membrane potential and Q-pool redox state (Fig. 5 *D*). Under oxidized conditions when the membrane potential is low, the most probable enzyme states are the fully oxidized state and one-electron reduced state. The two- and three-electron reduced states are elevated at high membrane potentials and when the Q pool is reduced. The four-electron reduced state becomes significant when the Q pool is extremely reduced, regardless of membrane potential. The five-electron reduced state reaches its maximum level when both the membrane potential is high and the Q pool is nearly 100% reduced, but even here the maximum level is <1%. This state only reaches significant levels when antimycin A is bound to the complex (data not shown). During cytochrome c reduction, the one-, two-, and three-electron reduced states are the most significant states (see Figs. S2 and S3). Other states play only a minor role in the net turnover of the enzyme. Superoxide is primarily produced through the three- and four-electron reduced states (see Fig. S4). Superoxide production from the five-electron reduced state is only important in the antimycin A-inhibited state. Finally, it is worth noting that under normal forward electron transfer conditions, the four-electron reduced state (E_4, see Fig. 1 *B*) can oxidize back to E_0 by two successive oxidization reactions by passing the four electrons to two quinone molecules bound at the Q_n sites, yielding two fully reduced quinols ($E_4 \rightarrow E_2 \rightarrow E_0$, *left side diagonal branches* in Fig. 1 *B*).

Physiological Q-pool operating range and cardiac *bc*$_1$ content

The redox state of the Q pool during rest, work, and pathophysiological conditions has been debated for decades. Some studies report the pool being mostly reduced (56,67,81,82), others report half-reduced (83,84), whereas a couple report mostly oxidized (85–87). The bell-shaped relationship between cytochrome c reduction and the Q-pool redox state makes either possibility feasible (see Fig. 5). In addition to this variability, estimates of bc_1 content range from 80 to 500 pmol/mg mitochondrial protein (85,88–91). With these uncertainties, extrapolating to an in vivo model is problematic. Fortunately, with our model, we can test 1) where the physiological operating Q-pool redox state of the bc_1 complex is, and 2) the influence of bc_1 protein content on cardiac mitochondria. To do this, we need to use data sets that measure all the relevant bioenergetic and product variables for the bc_1 reaction.

Very few such data sets exist; in our search, we only found two, the Böse data set (92) and the Vinnakota data set (93). Each data set probes mitochondrial function while monitoring essential bc_1 complex variables. They both report the rate of oxygen consumption (stoichiometrically linked to cytochrome c reduction), membrane potential, pH, and redox state of the cytochrome c pool. In addition, the NADH redox state is given for each condition. This variable is important to rule out nonphysiological redox states of the Q pool using a thermodynamic argument. Therefore, we can use these data sets to identify the Q-pool redox states capable of explaining the data for a wide range of bc_1 content. Fig. 6 shows model simulations using these data sets as fixed inputs for the model (cytochrome c redox state, membrane potential, and pH) while solving for the Q-pool redox state as a variable for each value of the bc_1 content to match the reported oxygen consumption rates (proportional to the turnover of the bc_1 complex). Because the QH_2-dependent kinetics are biphasic (parabolic, see Fig. 5 *A* and *upper panel* of Fig. 5 *C*), the model can explain the data for either a reduced or oxidized state of the Q pool. But when the Q pool is on the reduced side, Complex I will be operating in the so-called reverse electron transport mode (52,94). This mode is characteristic of extreme levels of superoxide production and only relevant in the disease state (95). Thus, we can infer that the normal operating redox state for the Q pool is that of mostly being oxidized when reducing substrates like glutamate and malate, or pyruvate, are present in excess. Also, the model simulations reveal that the physiological fluxes through the bc_1 complex in heart can be explained using bc_1 content in the range of 115–500 pmol/mg mitochondria. Below a bc_1 content of 115 pmol/mg, the model could not match the reported oxygen consumption rate for any Q-pool redox state using the inputs given by the data for the Vinnakota data set (see Fig. 5, *right column panels*). This range cannot be narrowed further due to the tight correlation between the bc_1 content and Q-pool redox state. To precisely quantify the bc_1 content in heart using these data, we would need either a direct measure of the bc_1 content in these preparations or data on the Q_{10} redox state for the experimental conditions used in these studies. Because the Q_{10} dissociation constants are only estimates,

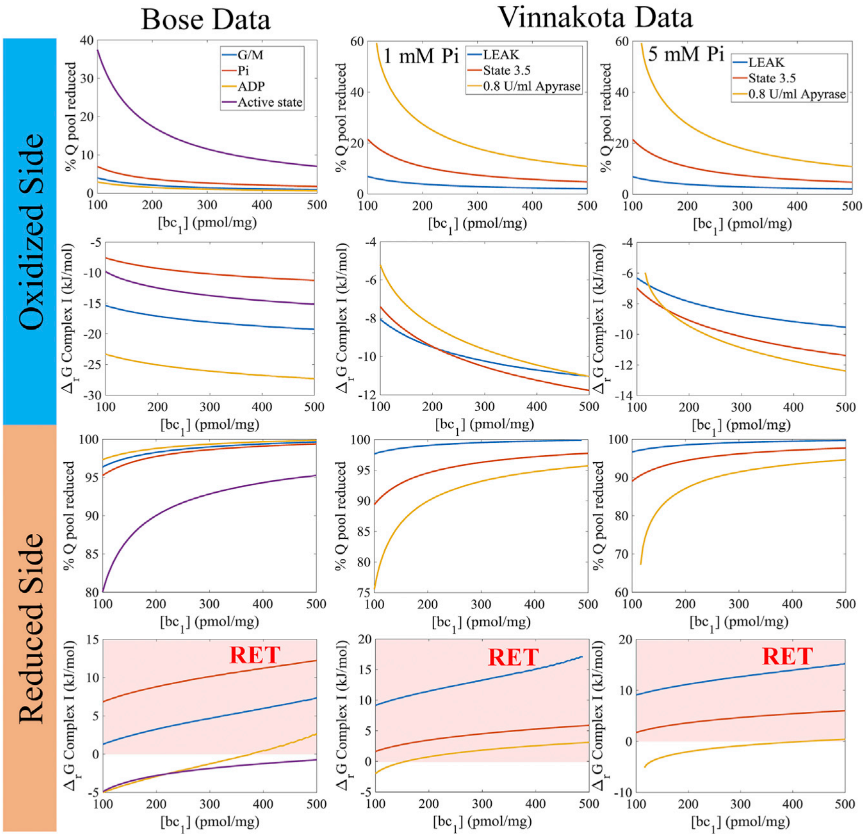

FIGURE 6 Model predictions of bc_1 content and Q-pool redox state during various bioenergetics states. Data reported in either Böse et al. (92) or Vinnakota et al. (93) were used to simulate the model using native Q_{10} binding constants given in Table S1. These data include membrane potential, pH, cytochrome *c* redox state, and NADH redox state for a given oxygen consumption rate. The redox state of the Q pool (% Q reduced) was treated as a variable and solved for, to match the reported oxygen consumption rates for the given conditions. The search was started from either the oxidized side or the reduced side to generate the two possible solutions. With the calculated redox state of the Q pool, the Complex I free energy of reaction was then computed. Whenever this value is >0, the Complex I reaction reverses. The standard free energy of reaction for Complex I at pH 7 was defined as −74.3 kJ/mol (see Table S1). Incomplete line segments indicate that no solution was found for the simulated conditions. Areas shaded in red (*bottom row*) designate regions where reverse electron transport (*RET*) occurs. This is a pathological condition linked to extremely high rates of free radical production. For the Böse data set, the "G/M" label refers to a condition where mitochondria were energized in the presence of 5 mM glutamate and 5 mM malate in the absence of Pi. The "Pi" label refers to the G/M condition, but in the presence of 3 mM Pi. The "ADP" label refers to the G/M condition in the presence of 1.3 mM ADP. The "Active state" label refers to the G/M conditions in the presence of 3 mM Pi and 1.3 mM ADP. For the Vinnakota data set, the "LEAK" label refers to a condition where mitochondria were energized in the presence of 2.5 mM pyruvate, 0.5 mM malate, and 5 mM ATP in the presence of 19 U/mL pyruvate kinase and 2 mM phosphoenolpyruvate to maximize the ATP/ADP ratio. The "State 3.5" label refers to the LEAK condition without pyruvate kinase and phosphoenolpyruvate where mitochondria are respiring to meet ATP demand due to residual ATPase activity in the preparation. The "0.8 U/mL Apyrase" label refers to the State 3.5 condition in the presence of 0.8 U/mL apyrase to maximally stimulate oxidative phosphorylation. For more details concerning the relevant mitochondrial variables and experimental conditions, see referenced studies. To see this figure in color, go online.

the more informative measurement would be the Q_{10} redox state. Unfortunately, this variable cannot be precisely quantified using current methods. Once we are able to make this measurement, we will have a more complete picture of the bioenergetic behavior of how the mitochondrial respiratory chain operates in both health and disease.

In summary, a functional dimer model of the bc_1 complex is presented. The model is capable of simulating the enzyme kinetics under a wide range of conditions and is calibrated with superoxide production data obtained using the purified complex and antimycin A-treated mitochondria. It was determined that Coulombic effects between intramonomer heme b_L and heme b_H and between intermonomer heme b_L and heme b_L were required to fit the data with a single, consistent set of parameters. In addition, model analysis supports the hypothesis that in normal steady-state conditions, only a single Q_p site in the dimer is operational per quinol oxidation. Model analysis demonstrates that the semireverse mode of superoxide production constitutes the major mechanism of free radical production by the bc_1 complex. The model also reveals that under physiological conditions, the Q pool is primarily in the oxidized state. However, in the presence of succinate or under pathological conditions, it can reach a significantly reduced state. Furthermore, the model was developed for the purpose of simulating mitochondrial metabolism as part of large-scale models. As this model is better calibrated and more faithful to the biophysics of the reaction compared to our previous models, more accurate simulations of free radical generation by the respiratory system will be possible.

SUPPORTING MATERIAL

Supporting Materials and Methods, nine figures, two tables, and one data file are available at http://www.biophysj.org/biophysj/supplemental/S0006-3495(17)30880-9.

ACKNOWLEDGMENTS

We are extremely grateful for the reviewers' comments and suggestions.

This work was supported by National Institutes of Health (NIH) grant R00-HL121160.

SUPPORTING CITATIONS

References (96–107) appear in the Supporting Material.

REFERENCES

1. Crofts, A. R., and E. A. Berry. 1998. Structure and function of the cytochrome bc_1 complex of mitochondria and photosynthetic bacteria. *Curr. Opin. Struct. Biol.* 8:501–509.
2. Nicholls, D. G., and S. J. Ferguson. 2013. Bioenergetics 4. *Bioenergetics.* 4:1–419.
3. Hatefi, Y. 1985. The mitochondrial electron transport and oxidative phosphorylation system. *Annu. Rev. Biochem.* 54:1015–1069.
4. Gnaiger, E. 2009. Capacity of oxidative phosphorylation in human skeletal muscle: new perspectives of mitochondrial physiology. *Int. J. Biochem. Cell Biol.* 41:1837–1845.
5. Xia, D., C. A. Yu, …, J. Deisenhofer. 1997. Crystal structure of the cytochrome bc_1 complex from bovine heart mitochondria. *Science.* 277:60–66.
6. Xia, D., H. Kim, …, J. Deisenhofer. 1998. A novel electron transfer mechanism suggested by crystallographic studies of mitochondrial cytochrome bc_1 complex. *Biochem. Cell Biol.* 76:673–679.
7. Yu, C. A., J. Z. Xia, …, J. Deisenhofer. 1996. Crystallization and preliminary structure of beef heart mitochondrial cytochrome-bc_1 complex. *Biochim. Biophys. Acta.* 1275:47–53.
8. Yu, C. A., L. Zhang, …, L. Yu. 1999. Structure and reaction mechanisms of multifunctional mitochondrial cytochrome bc_1 complex. *Biofactors.* 9:103–109.
9. Gao, X., X. Wen, …, D. Xia. 2003. Structural basis for the quinone reduction in the bc_1 complex: a comparative analysis of crystal structures of mitochondrial cytochrome bc_1 with bound substrate and inhibitors at the Q_i site. *Biochemistry.* 42:9067–9080.
10. Huang, L. S., D. Cobessi, …, E. A. Berry. 2005. Binding of the respiratory chain inhibitor antimycin to the mitochondrial bc_1 complex: a new crystal structure reveals an altered intramolecular hydrogen-bonding pattern. *J. Mol. Biol.* 351:573–597.
11. Lange, C., and C. Hunte. 2002. Crystal structure of the yeast cytochrome bc_1 complex with its bound substrate cytochrome *c*. *Proc. Natl. Acad. Sci. USA.* 99:2800–2805.
12. Elberry, M., K. Xiao, …, C. A. Yu. 2006. Generation, characterization and crystallization of a highly active and stable cytochrome bc_1 complex mutant from *Rhodobacter sphaeroides*. *Biochim. Biophys. Acta.* 1757:835–840.
13. Esser, L., F. Zhou, …, D. Xia. 2016. Hydrogen bonding to the substrate is not required for Rieske iron-sulfur protein docking to the quinol oxidation site of complex III. *J. Biol. Chem.* 291:25019–25031.
14. Crofts, A. R. 2004. The cytochrome bc_1 complex: function in the context of structure. *Annu. Rev. Physiol.* 66:689–733.
15. Xia, D., L. Esser, …, C. A. Yu. 2013. Structural analysis of cytochrome bc_1 complexes: implications to the mechanism of function. *Biochim. Biophys. Acta.* 1827:1278–1294.
16. Esser, L., C. A. Yu, and D. Xia. 2014. Structural basis of resistance to anti-cytochrome bc_1 complex inhibitors: implication for drug improvement. *Curr. Pharm. Des.* 20:704–724.
17. Zhu, J., T. Egawa, …, C. A. Yu. 2007. Simultaneous reduction of iron-sulfur protein and cytochrome b_L during ubiquinol oxidation in cytochrome bc_1 complex. *Proc. Natl. Acad. Sci. USA.* 104:4864–4869.
18. Trumpower, B. L. 2002. A concerted, alternating sites mechanism of ubiquinol oxidation by the dimeric cytochrome bc_1 complex. *Biochim. Biophys. Acta.* 1555:166–173.
19. Osyczka, A., C. C. Moser, and P. L. Dutton. 2005. Fixing the Q cycle. *Trends Biochem. Sci.* 30:176–182.
20. Crofts, A. R., S. W. Rose, …, S. A. Dikanov. 2017. The Q-cycle mechanism of the bc_1 complex: a biologist's perspective on atomistic studies. *J. Phys. Chem. B.* 121:3701–3717.
21. Crofts, A. R., S. Hong, …, K. Schulten. 2013. The mechanism of ubihydroquinone oxidation at the Q_o-site of the cytochrome bc_1 complex. *Biochim. Biophys. Acta.* 1827:1362–1377.
22. Cape, J. L., M. K. Bowman, and D. M. Kramer. 2007. A semiquinone intermediate generated at the Q_o site of the cytochrome bc_1 complex: importance for the Q-cycle and superoxide production. *Proc. Natl. Acad. Sci. USA.* 104:7887–7892.
23. Vennam, P. R., N. Fisher, …, M. K. Bowman. 2013. A caged, destabilized, free radical intermediate in the Q-cycle. *ChemBioChem.* 14:1745–1753.
24. Zhang, H., A. Osyczka, …, C. C. Moser. 2007. Exposing the complex III Q_o semiquinone radical. *Biochim. Biophys. Acta.* 1767:883–887.
25. Sarewicz, M., M. Dutka, …, A. Osyczka. 2013. Triplet state of the semiquinone-Rieske cluster as an intermediate of electronic bifurcation catalyzed by cytochrome bc_1. *Biochemistry.* 52:6388–6395.
26. Pietras, R., M. Sarewicz, and A. Osyczka. 2016. Distinct properties of semiquinone species detected at the ubiquinol oxidation Q_o site of cytochrome bc_1 and their mechanistic implications. *J. R. Soc. Interface.* 13:20160133.
27. Mulkidjanian, A. Y. 2010. Activated Q-cycle as a common mechanism for cytochrome bc_1 and cytochrome $b6_f$ complexes. *Biochim. Biophys. Acta.* 1797:1858–1868.
28. Rottenberg, H., R. Covian, and B. L. Trumpower. 2009. Membrane potential greatly enhances superoxide generation by the cytochrome bc_1 complex reconstituted into phospholipid vesicles. *J. Biol. Chem.* 284:19203–19210.
29. Liu, S. S. 2010. Mitochondrial Q cycle-derived superoxide and chemiosmotic bioenergetics. *Ann. N. Y. Acad. Sci.* 1201:84–95.
30. Quinlan, C. L., A. A. Gerencser, …, M. D. Brand. 2011. The mechanism of superoxide production by the antimycin-inhibited mitochondrial Q-cycle. *J. Biol. Chem.* 286:31361–31372.
31. Covian, R., E. B. Gutierrez-Cirlos, and B. L. Trumpower. 2004. Anti-cooperative oxidation of ubiquinol by the yeast cytochrome bc_1 complex. *J. Biol. Chem.* 279:15040–15049.
32. Osyczka, A., C. C. Moser, …, P. L. Dutton. 2004. Reversible redox energy coupling in electron transfer chains. *Nature.* 427:607–612.
33. Castellani, M., R. Covian, …, B. L. Trumpower. 2010. Direct demonstration of half-of-the-sites reactivity in the dimeric cytochrome bc_1 complex: enzyme with one inactive monomer is fully active but unable to activate the second ubiquinol oxidation site in response to ligand binding at the ubiquinone reduction site. *J. Biol. Chem.* 285:502–510.
34. Lanciano, P., D. W. Lee, …, F. Daldal. 2011. Intermonomer electron transfer between the low-potential *b* hemes of cytochrome bc_1. *Biochemistry.* 50:1651–1663.
35. Swierczek, M., E. Cieluch, …, A. Osyczka. 2010. An electronic bus bar lies in the core of cytochrome bc_1. *Science.* 329:451–454.
36. Hong, S., D. Victoria, and A. R. Crofts. 2012. Inter-monomer electron transfer is too slow to compete with monomeric turnover in bc_1 complex. *Biochim. Biophys. Acta.* 1817:1053–1062.
37. Crofts, A. R., J. T. Holland, …, M. G. Kuras. 2008. The Q-cycle reviewed: how well does a monomeric mechanism of the bc_1 complex account for the function of a dimeric complex? *Biochim. Biophys. Acta.* 1777:1001–1019.
38. Ekiert, R., M. Czapla, …, A. Osyczka. 2014. Hybrid fusions show that inter-monomer electron transfer robustly supports cytochrome bc_1 function in vivo. *Biochem. Biophys. Res. Commun.* 451:270–275.
39. Beard, D. A. 2005. A biophysical model of the mitochondrial respiratory system and oxidative phosphorylation. *PLOS Comput. Biol.* 1:e36.
40. Wu, F., F. Yang, …, D. A. Beard. 2007. Computer modeling of mitochondrial tricarboxylic acid cycle, oxidative phosphorylation, metabolite transport, and electrophysiology. *J. Biol. Chem.* 282:24525–24537.

41. Esposti, M. D., and G. Lenaz. 1991. The kinetic mechanism of ubiquinol: cytochrome *c* reductase at steady state. *Arch. Biochem. Biophys.* 289:303–312.

42. Fato, R., M. Cavazzoni, …, G. Lenaz. 1993. Steady-state kinetics of ubiquinol-cytochrome *c* reductase in bovine heart submitochondrial particles: diffusional effects. *Biochem. J.* 290:225–236.

43. Kubota, T., S. Yoshikawa, and H. Matsubara. 1992. Kinetic mechanism of beef heart ubiquinol:cytochrome *c* oxidoreductase. *J. Biochem.* 111:91–98.

44. Speck, S. H., and E. Margoliash. 1984. Characterization of the interaction of cytochrome *c* and mitochondrial ubiquinol-cytochrome *c* reductase. *J. Biol. Chem.* 259:1064–1072.

45. Tan, A. K., R. R. Ramsay, …, H. Miyoshi. 1993. Comparison of the structures of the quinone-binding sites in beef heart mitochondria. *J. Biol. Chem.* 268:19328–19333.

46. Reed, J. S., and C. I. Ragan. 1987. The effect of rate limitation by cytochrome *c* on the redox state of the ubiquinone pool in reconstituted NADH: cytochrome *c* reductase. *Biochem. J.* 247:657–662.

47. Demin, O. V., B. N. Kholodenko, and V. P. Skulachev. 1998. A model of O_{2-} generation in the complex III of the electron transport chain. *Mol. Cell. Biochem.* 184:21–33.

48. Orii, Y., and T. Miki. 1997. Oxidation process of bovine heart ubiquinol-cytochrome *c* reductase as studied by stopped-flow rapid-scan spectrophotometry and simulations based on the mechanistic Q cycle model. *J. Biol. Chem.* 272:17594–17604.

49. Selivanov, V. A., T. V. Votyakova, …, M. Cascante. 2009. Bistability of mitochondrial respiration underlies paradoxical reactive oxygen species generation induced by anoxia. *PLOS Comput. Biol.* 5:e1000619.

50. Guillaud, F., S. Dröse, …, E. Klipp. 2014. Superoxide production by cytochrome bc_1 complex: a mathematical model. *Biochim. Biophys. Acta.* 1837:1643–1652.

51. Gauthier, L. D., J. L. Greenstein, …, R. L. Winslow. 2013. A computational model of reactive oxygen species and redox balance in cardiac mitochondria. *Biophys. J.* 105:1045–1056.

52. Bazil, J. N., V. R. Pannala, …, D. A. Beard. 2014. Determining the origins of superoxide and hydrogen peroxide in the mammalian NADH:ubiquinone oxidoreductase. *Free Radic. Biol. Med.* 77:121–129.

53. Bazil, J. N., K. C. Vinnakota, …, D. A. Beard. 2013. Analysis of the kinetics and bistability of ubiquinol:cytochrome *c* oxidoreductase. *Biophys. J.* 105:343–355.

54. Shinkarev, V. P., A. R. Crofts, and C. A. Wraight. 2001. The electric field generated by photosynthetic reaction center induces rapid reversed electron transfer in the bc_1 complex. *Biochemistry.* 40:12584–12590.

55. Brandt, U., and J. G. Okun. 1997. Role of deprotonation events in ubihydroquinone:cytochrome *c* oxidoreductase from bovine heart and yeast mitochondria. *Biochemistry.* 36:11234–11240.

56. Brown, G. C., and M. D. Brand. 1985. Thermodynamic control of electron flux through mitochondrial cytochrome bc_1 complex. *Biochem. J.* 225:399–405.

57. Dröse, S., and U. Brandt. 2008. The mechanism of mitochondrial superoxide production by the cytochrome bc_1 complex. *J. Biol. Chem.* 283:21649–21654.

58. Goncalves, R. L., C. L. Quinlan, …, M. D. Brand. 2015. Sites of superoxide and hydrogen peroxide production by muscle mitochondria assessed ex vivo under conditions mimicking rest and exercise. *J. Biol. Chem.* 290:209–227.

59. Leguijt, T., P. W. Engels, …, K. J. Hellingwerf. 1993. Abundance, subunit composition, redox properties, and catalytic activity of the cytochrome bc_1 complex from alkaliphilic and halophilic, photosynthetic members of the family *Ectothiorhodospiraceae*. *J. Bacteriol.* 175:1629–1636.

60. Rich, P. R., and D. S. Bendall. 1980. The kinetics and thermodynamics of the reduction of cytochrome *c* by substituted *p*-benzoquinols in solution. *Biochim. Biophys. Acta.* 592:506–518.

61. Crofts, A. R. 2004. Proton-coupled electron transfer at the Q_o site of the bc_1 complex controls the rate of ubihydroquinone oxidation. *Biochim. Biophys. Acta.* 1655:77–92.

62. Ding, H., C. C. Moser, …, P. L. Dutton. 1995. Ubiquinone pair in the Q_o site central to the primary energy conversion reactions of cytochrome bc_1 complex. *Biochemistry.* 34:15979–15996.

63. Wikström, M. 2005. Biophysical and Structural Aspects of Bioenergetics. Royal Society of Chemistry, Cambridge, UK.

64. Rich, P. R. 1984. Electron and proton transfers through quinones and cytochrome *bc* complexes. *Biochim. Biophys. Acta.* 768:53–79.

65. Starkov, A. A., and G. Fiskum. 2003. Regulation of brain mitochondrial H_2O_2 production by membrane potential and NAD(P)H redox state. *J. Neurochem.* 86:1101–1107.

66. Liu, Y., G. Fiskum, and D. Schubert. 2002. Generation of reactive oxygen species by the mitochondrial electron transport chain. *J. Neurochem.* 80:780–787.

67. Quinlan, C. L., I. V. Perevoshchikova, …, M. D. Brand. 2013. Sites of reactive oxygen species generation by mitochondria oxidizing different substrates. *Redox Biol.* 1:304–312.

68. Sarewicz, M., A. Borek, …, A. Osyczka. 2010. Discrimination between two possible reaction sequences that create potential risk of generation of deleterious radicals by cytochrome bc_1. Implications for the mechanism of superoxide production. *Biochim. Biophys. Acta.* 1797:1820–1827.

69. Borek, A., M. Sarewicz, and A. Osyczka. 2008. Movement of the iron-sulfur head domain of cytochrome bc_1 transiently opens the catalytic Q_o site for reaction with oxygen. *Biochemistry.* 47:12365–12370.

70. Snyder, C. H., E. B. Gutierrez-Cirlos, and B. L. Trumpower. 2000. Evidence for a concerted mechanism of ubiquinol oxidation by the cytochrome *bc*1 complex. *J. Biol. Chem.* 275:13535–13541.

71. Cooley, J. W., D. W. Lee, and F. Daldal. 2009. Across membrane communication between the Q_o and Q_i active sites of cytochrome bc_1. *Biochemistry.* 48:1888–1899.

72. Cooley, J. W., T. Ohnishi, and F. Daldal. 2005. Binding dynamics at the quinone reduction Q_i site influence the equilibrium interactions of the iron sulfur protein and hydroquinone oxidation Q_o site of the cytochrome bc_1 complex. *Biochemistry.* 44:10520–10532.

73. Howell, N., and D. E. Robertson. 1993. Electrochemical and spectral analysis of the long-range interactions between the Q_o and Q_i sites and the heme prosthetic groups in ubiquinol-cytochrome *c* oxidoreductase. *Biochemistry.* 32:11162–11172.

74. Crofts, A. R., V. P. Shinkarev, …, S. Hong. 2003. The modified Q cycle explains the apparent mismatch between the kinetics of reduction of cytochromes *c*1 and b_H in the *bc*1 complex. *J. Biol. Chem.* 278:36191–36201.

75. Hong, S., W. B. de Almeida, …, A. R. Crofts. 2014. The semiquinone at the Q_i site of the bc_1 complex explored using HYSCORE spectroscopy and specific isotopic labeling of ubiquinone in *Rhodobacter sphaeroides* via ^{13}C methionine and construction of a methionine auxotroph. *Biochemistry.* 53:6022–6031.

76. Samoilova, R. I., A. R. Crofts, and S. A. Dikanov. 2011. Reaction of superoxide radical with quinone molecules. *J. Phys. Chem. A.* 115:11589–11593.

77. Sun, J., and B. L. Trumpower. 2003. Superoxide anion generation by the cytochrome bc_1 complex. *Arch. Biochem. Biophys.* 419:198–206.

78. Covian, R., and B. L. Trumpower. 2008. Regulatory interactions in the dimeric cytochrome bc_1 complex: the advantages of being a twin. *Biochim. Biophys. Acta.* 1777:1079–1091.

79. Li, L., C. Li, …, E. Alexov. 2013. On the dielectric "constant" of proteins: smooth dielectric function for macromolecular modeling and its implementation in delPhi. *J. Chem. Theory Comput.* 9:2126–2136.

80. Esser, L., X. Gong, …, D. Xia. 2006. Surface-modulated motion switch: capture and release of iron-sulfur protein in the cytochrome bc_1 complex. *Proc. Natl. Acad. Sci. USA.* 103:13045–13050.

81. Matsura, T., K. Yamada, and T. Kawasaki. 1992. Antioxidant role of cellular reduced coenzyme *Q* homologs and α-tocopherol in free radical-induced injury of hepatocytes isolated from rats fed diets with different vitamin E contents. *Biochim. Biophys. Acta.* 1127:277–283.

82. Takada, M., S. Ikenoya, …, K. Katayama. 1984. Simultaneous determination of reduced and oxidized ubiquinones. *Methods Enzymol.* 105:147–155.

83. Aberg, F., E. L. Appelkvist, …, L. Ernster. 1992. Distribution and redox state of ubiquinones in rat and human tissues. *Arch. Biochem. Biophys.* 295:230–234.

84. Kim, N., M. O. Ripple, and R. Springett. 2012. Measurement of the mitochondrial membrane potential and pH gradient from the redox poise of the hemes of the bc_1 complex. *Biophys. J.* 102:1194–1203.

85. Benard, G., B. Faustin, …, R. Rossignol. 2006. Physiological diversity of mitochondrial oxidative phosphorylation. *Am. J. Physiol. Cell Physiol.* 291:C1172–C1182.

86. Galinier, A., A. Carrière, …, L. Casteilla. 2004. Biological validation of coenzyme Q redox state by HPLC-EC measurement: relationship between coenzyme Q redox state and coenzyme Q content in rat tissues. *FEBS Lett.* 578:53–57.

87. Bazil, J. N., D. A. Beard, and K. C. Vinnakota. 2016. Catalytic coupling of oxidative phosphorylation, ATP demand, and reactive oxygen species generation. *Biophys. J.* 110:962–971.

88. Schwerzmann, K., L. M. Cruz-Orive, …, E. R. Weibel. 1986. Molecular architecture of the inner membrane of mitochondria from rat liver: a combined biochemical and stereological study. *J. Cell Biol.* 102:97–103.

89. Gellerfors, P., T. Johansson, and B. D. Nelson. 1981. Isolation of the cytochrome-bc_1 complex from rat-liver mitochondria. *Eur. J. Biochem.* 115:275–278.

90. Iwata, S., S. Seki, and T. Oda. 1967. Flavin and cytochrome contents in the mitochondria of the heart and liver. *Acta Med. Okayama.* 21:191–203.

91. Schägger, H., and K. Pfeiffer. 2001. The ratio of oxidative phosphorylation complexes I-V in bovine heart mitochondria and the composition of respiratory chain supercomplexes. *J. Biol. Chem.* 276:37861–37867.

92. Böse, S., S. French, …, R. S. Balaban. 2003. Metabolic network control of oxidative phosphorylation: multiple roles of inorganic phosphate. *J. Biol. Chem.* 278:39155–39165.

93. Vinnakota, K. C., J. N. Bazil, …, D. A. Beard. 2016. Feedback regulation and time hierarchy of oxidative phosphorylation in cardiac mitochondria. *Biophys. J.* 110:972–980.

94. Hirst, J., M. S. King, and K. R. Pryde. 2008. The production of reactive oxygen species by complex I. *Biochem. Soc. Trans.* 36:976–980.

95. Chouchani, E. T., V. R. Pell, …, M. P. Murphy. 2014. Ischaemic accumulation of succinate controls reperfusion injury through mitochondrial ROS. *Nature.* 515:431–435.

96. Rich, P. R., A. E. Jeal, …, A. J. Moody. 1990. Inhibitor effects on redox-linked protonations of the *b* haems of the mitochondrial bc_1 complex. *Biochim. Biophys. Acta.* 1018:29–40.

97. Eddowes, M. J., and H. A. O. Hill. 1979. Electrochemistry of horse heart cytochrome-*c*. *J. Am. Chem. Soc.* 101:4461–4464.

98. Urban, P. F., and M. Klingenberg. 1969. On the redox potentials of ubiquinone and cytochrome *b* in the respiratory chain. *Eur. J. Biochem.* 9:519–525.

99. Ohnishi, T., and B. L. Trumpower. 1980. Differential effects of antimycin on ubisemiquinone bound in different environments in isolated succinate-cytochrome *c* reductase complex. *J. Biol. Chem.* 255:3278–3284.

100. Brandt, U. 2006. Energy converting NADH:quinone oxidoreductase (complex I). *Annu. Rev. Biochem.* 75:69–92.

101. Glaser, E. G., S. W. Meinhardt, and A. R. Crofts. 1984. Reduction of cytochrome b_{561} through the antimycin-sensitive site of the ubiquinol-cytochrome c_2 oxidoreductase complex of *Rhodopseudomonas sphaeroides*. *FEBS Lett.* 178:336–342.

102. Glaser, E. G., and A. R. Crofts. 1984. A new electrogenic step in the ubiquinol:cytochrome c_2 oxidoreductase complex of *Rhodopseudomonas sphaeroides*. *Biochim. Biophys. Acta.* 766:322–333.

103. Robertson, D. E., and P. L. Dutton. 1988. The nature and magnitude of the charge-separation reactions of ubiquinol cytochrome c_2 oxidoreductase. *Biochim. Biophys. Acta.* 935:273–291.

104. Lass, A., S. Agarwal, and R. S. Sohal. 1997. Mitochondrial ubiquinone homologues, superoxide radical generation, and longevity in different mammalian species. *J. Biol. Chem.* 272:19199–19204.

105. Murphy, M. P. 2009. How mitochondria produce reactive oxygen species. *Biochem. J.* 417:1–13.

106. Kuo-chen, C., and J. Shou-ping. 1974. Studies on the rate of diffusion-controlled reactions of enzymes. Spatial factor and force field factor. *Sci. Sin.* 27:664–680.

107. Squire, W., and G. Trapp. 1998. Using complex variables to estimate derivatives of real functions. *SIAM Rev.* 40:110–112.

108. Schrodinger. 2015. The PyMOL Molecular Graphics System, Version 1.8. Schrodinger, LLC, New York, NY.

Biophysical Journal, Volume *113*

Supplemental Information

Analysis of a Functional Dimer Model of Ubiquinol Cytochrome *c* Oxidoreductase

Jason N. Bazil

Supporting Material

Analysis of a Functional Dimer Model of Ubiquinol Cytochrome c Oxidoreductase

Jason N. Bazil

Michigan State University, Department of Physiology, East Lansing, MI

Table S1 shows the fixed model parameters used in the simulations given in the main text and in this supplement.

Table S1. Fixed Parameters

Parameter	Definition	Value[a]	Units	Reference
		Physical Constants		
R	Ideal gas constant	8.314	J/mol/K	-
F	Faraday's constant	96.5	J/mol/mV	-
		K_A values		
K_A^{ISPox}	Oxidized Rieske iron-sulfur cluster acidic group protonation constant	$10^{-6.6}$	-	(1)
K_B^{ISPox}	Oxidized Rieske iron-sulfur cluster basic group protonation constant	$10^{-9.2}$	-	(1)
$K_A^{b_L ox}$	Oxidized heme b_L protonation constant	$10^{-5.9}$	-	(2)
$K_A^{b_L red}$	Reduced heme b_L protonation constant	$10^{-7.9}$	-	(2)
$K_A^{b_H ox}$	Oxidized heme b_H protonation constant	$10^{-5.7}$	-	(2)
$K_A^{b_H red}$	Reduced heme b_H protonation constant	$10^{-7.7}$	-	(2)
Thermodynamic				
$E_m(c^{3+}/c^{2+})$	Cytochrome c midpoint potential	255	mV	(3)
$E_m(c_1^{3+}/c_1^{2+})$	Cytochrome c_1 midpoint potential	270	mV	(4)
$E_m^7(ISP_{ox}/ISP_{red})$	Rieske ISP midpoint potential	300	mV	(4)
$E_m^0(Q/QH_2)$	Ubiquinone midpoint potential	65 (479)	mV	(5)
$E_m^0(b_L^{3+}/b_L^{2+})$	Cytochrome b_L midpoint potential	-30 (39)	mV	(2)
$E_m^0(b_H^{3+}/b_H^{2+})$	Cytochrome b_H midpoint potential	90 (160)	mV	(2)
$E_m^0(Q/SQ)_n$	Qn-site semiquinone midpoint potential	-2.3	mV	(6)
$E_m^0(SQ/QH_2)_n$	Qn-site semiquinone midpoint potential	122 (951)	mV	(6)
$E_m^7(NAD^+/NADH)$	NADH midpoint potential	-320	mV	(7)
β	Fraction of total charge translocation sensed between bL and bH	0.5	-	(2, 8-10)
		Q_{10} Related Constants		
$K_{DQ_p}^{Q_{10}H_2}$	$Q_{10}H_2$ binding constant at Qp-site	1.6	mM	(11)
$K_{DQ_p}^{Q_{10}}$	Q_{10} binding constant at Qp-site	1.6	mM	(11)

$K_{DQ_n}^{Q_{10}}$	Q_{10} binding constant at Qn-site	0.25	mM	(12)[b]
$K_{DQ_n}^{Q_{10}H_2}$	$Q_{10}H_2$ binding constant at Qn-site	2.5	μM	(12)[b]
$Q_{10,tot}$	Q_{10} pool size	20	mM	(13, 14)[c]
Superoxide				
$E_m(O_2/O_2^{\bullet-})$	Superoxide midpoint potential	-160	mV	(15)[d]
kf_{SO}	Superoxide production 2nd order rate constant	10^{10}	$M^{-1}s^{-1}$	(16)

[a]Values are at pH 7 and at 25 °C. Values in parenthesis are at pH 0 and 25 °C. [b]Values chosen to match reported kinetic and thermodynamic behavior of enzyme. [c]Estimated by assuming a total mitochondrial Q pool of 5.8 nmol/mg and an inner membrane volume of 290 nl/mg. [d]The pK of superoxide is 4.7, so the midpoint potential is invariant in the biological pH range.

Table S2. Experimental Data Summary

Species and Tissue Origin	Quinone Substrate	Kinetic Data	Buffer pH	Membrane Potential (mV)	Free Radical Data	Dimeric Function Data	Reference
Bovine heart	DQ	yes	7.5	0	no	no	(17)
Bovine heart	NBH	yes	7.2	0	no	no	(1)
Bovine heart	Q_2	yes	7.4	0	no	no	(18)
Bovine heart	Q_2	yes	7.4	0	no	no	(19)
Rat liver	Q_2 and Q_{10}	yes	7.0	100 - 180	no	no	(20)
Yeast	DQ	no	7.3	100 - 220	yes	no	(21)
Rat skeletal muscle	Q_{10}	no	7.2	0	yes	no	(22)
Yeast	DQ	no	7.0	0	no	yes	(23)
Bovine heart	DQ	no	7.4	0	yes	no	(24)

Q_2, ubiquinone-2; NBH, nonylubihydroquinone; DQ, decylubiquinone; Q_{10}, ubiquonone-10

Table S2 lists the data used to parameterize the model. The list consists of a set of data that contain information on the cytochrome c reduction kinetics and superoxide production rates collected under a wide range of conditions. This is not an exhaustive list of all available data, but it does contain data that enables identification of essential features of bc1 kinetics and free radical generation. The majority of the data come from bc1 complex purified from bovine heart, but some of the superoxide production and dimeric function data were obtained using rat and yeast mitochondria. Ideally, these data would be from the same source as the kinetic data. But unfortunately, we could not find such data. The data was collected using bc1 complex resuspended in detergent, incorporated into lipid membranes, or in its original state in mitochondrial membranes. The kinetic data were collected with a variety of Q-analogues at different pH and membrane energization conditions. The superoxide production data was collected in a similar manner. By fitting all these data with the same set of rate constants and relevant kinetic parameters, the robustness of the model is demonstrated.

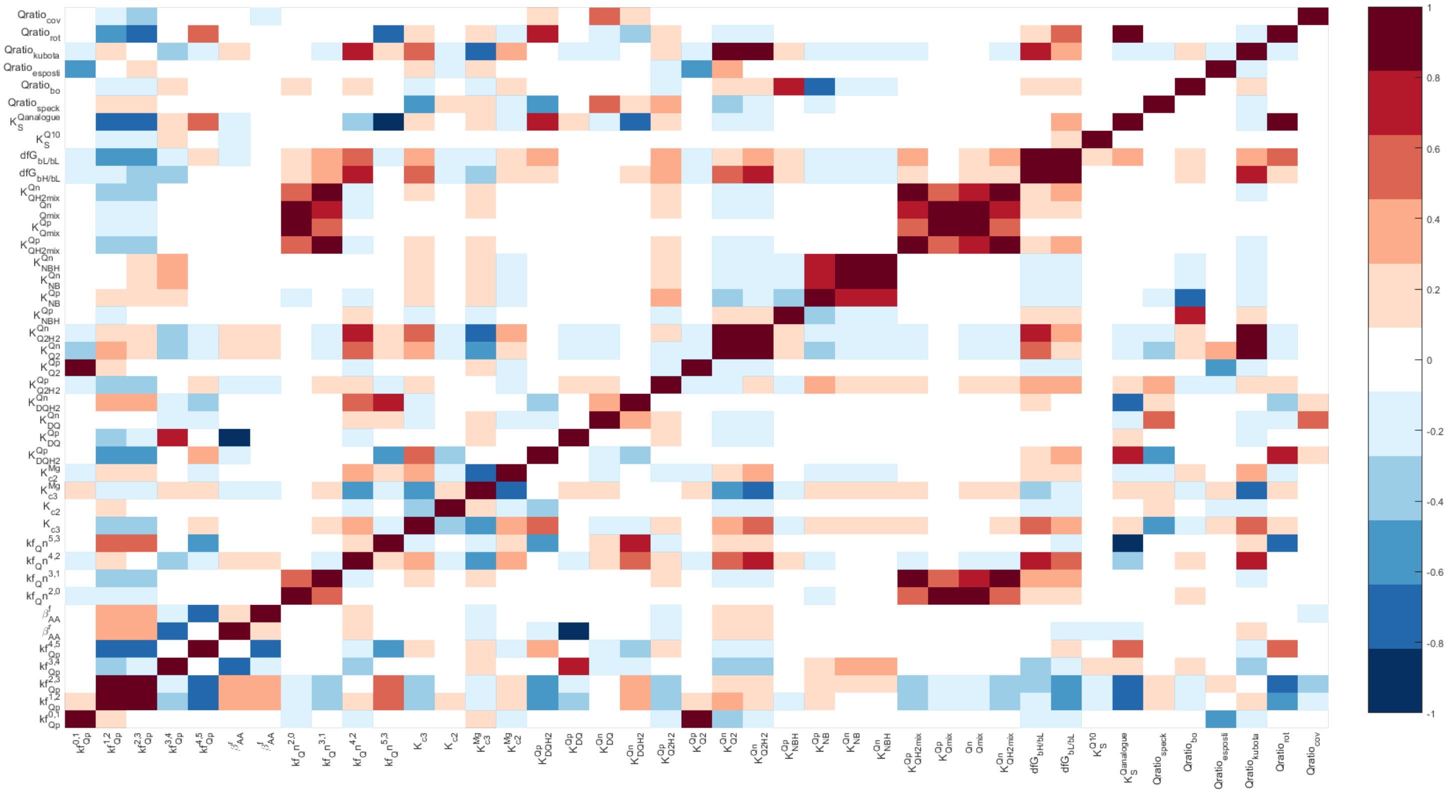

Figure S1. Correlation heat map for adjustable parameters given in Table 1. Parameter correlation coefficients are ordered in the following manner: state transition rate constants, cytochrome c binding constants, quinone analogue binding constants, quinone stability constants, and experimental design parameters. Parameter correlation coefficients were computed from the parameter sensitivity matrix (see Eq. S44). Parameters can be negatively correlated (red pixels, coefficient value of -1), uncorrelated (white pixels, coefficient value of 0), or positively correlated (blue pixels coefficient value of 1).

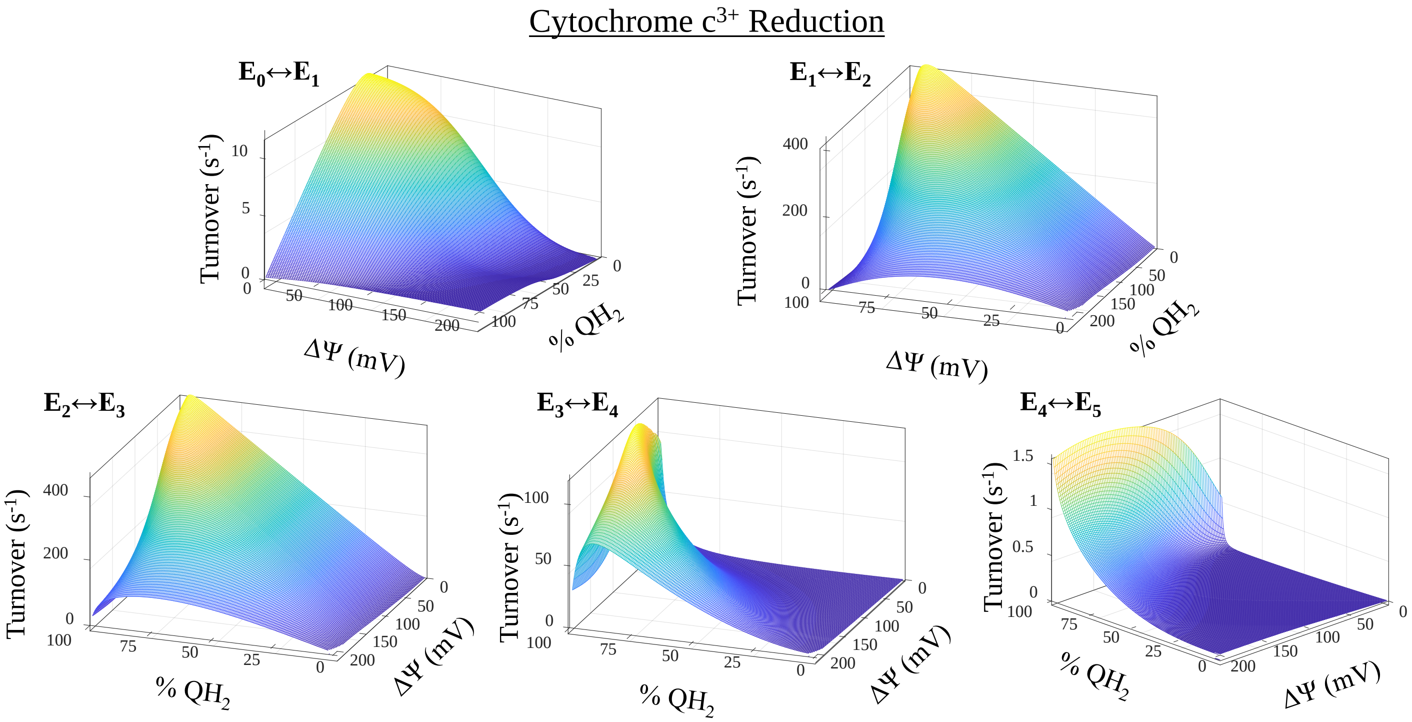

Figure S2. Net cytochrome c^{3+} reduction turnover rates at the Qp-site. The partial reaction for each enzyme state transition is given as indicated. The conditions for these simulations are given in Figure 5.

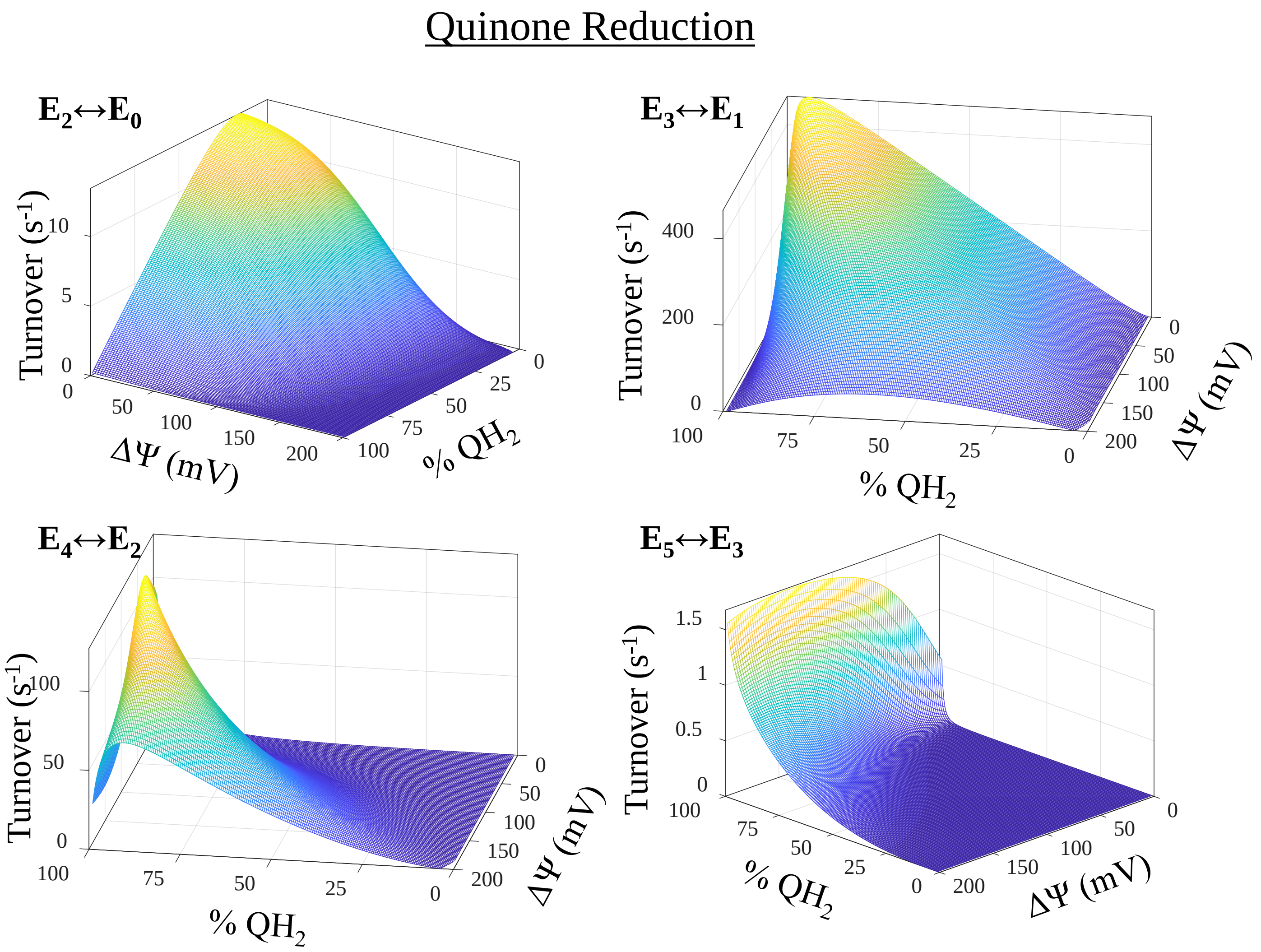

Figure S3. Net quinone reduction turnover rates at the Qn-site. The partial reaction for each each enzyme state transition is given as indicated. The conditions for these simulations are given in Figure 5.

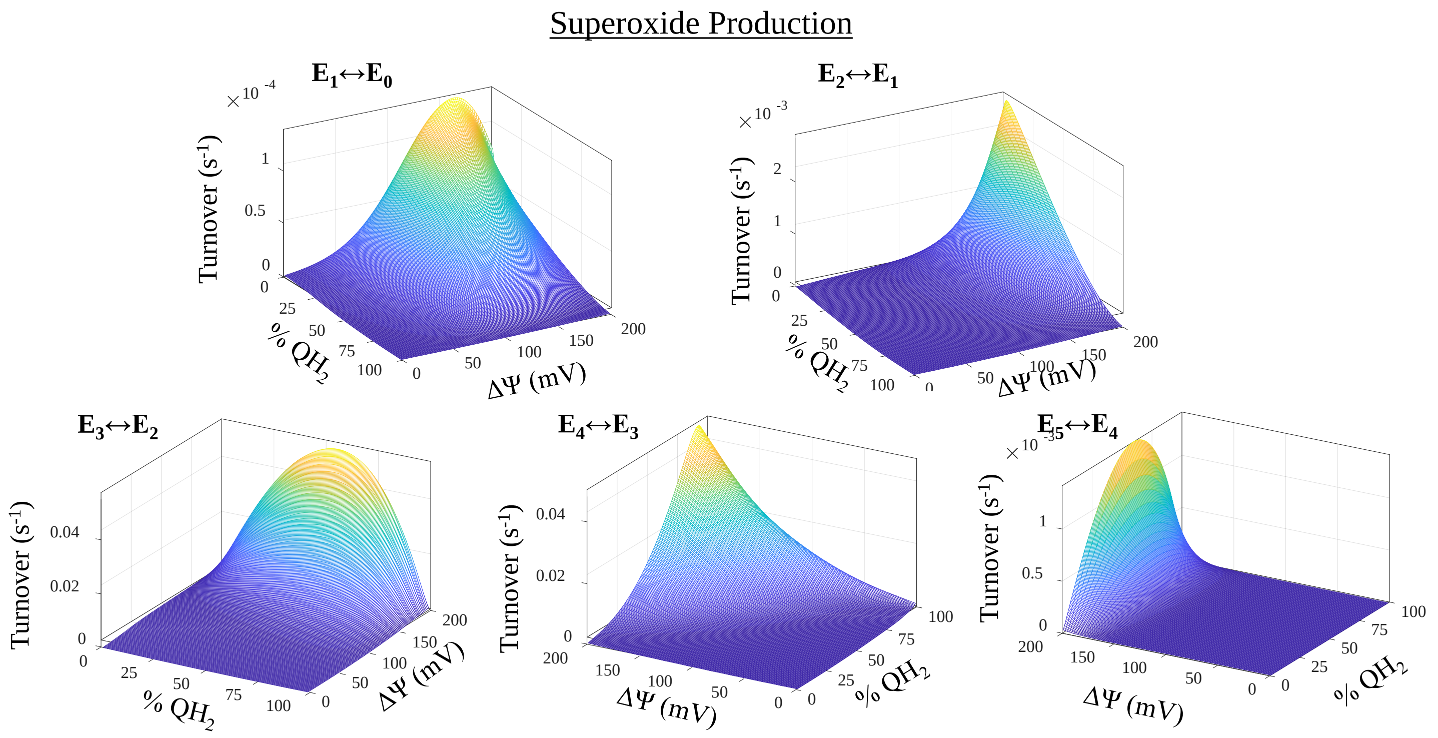

Figure S4. Net superoxide production turnover rates at the Qp-site. The partial reaction for each enzyme state transition is given as indicated. The conditions for these simulations are given in Figure 5.

Midpoint potential to free energy conversions. The midpoint potentials given in Table S1 were converted to free energies using the following equation.

$$\Delta G = -nF\Delta E \qquad \text{Eq. S1}$$

where *n* is the number of electrons transferred in the reaction.

Free energy of electron transfer and pH effects. The free energy of reaction for the mobile electron carriers (quinones and cytochrome c) are defined with respect to their respective pools. So the free energy of electron transfer (midpoint potentials when expressed in mV) for these electron carriers must be adjusted for the free energy of binding and unbinding to the bc1 complex. For the cytochrome c^{3+}/c^{2+} reduction free energy, the free energy of electron transfer is calculated using Eq. S2.

$$\Delta_{et}G_{c^{3+}/c^{2+}} = \Delta G^{0}_{c^{3+}/c^{2+}} - RT\ln(KD_{c^{3+}}) + RT\ln(KD_{c^{2+}}) \qquad \text{Eq. S2}$$

For the quinone/semiquinone and the semiqinone/quinol reduction free energy, the free energy of electron transfer is calculated in a similar manner using Eqs. S3 and S4.

$$\Delta_{et}G_{Q/SQ} = \Delta G^{0}_{Q/SQ} - RT\ln(K_{Q}) \qquad \text{Eq. S3}$$

$$\Delta_{et}G_{SQ/QH_2} = \Delta G^{pH}_{SQ/QH_2} + RT\ln(K_{QH_2}) \qquad \text{Eq. S4}$$

The free energy for the semiquinone/quinol couple is pH dependent and calculated using Eq. S5.

$$\Delta G^{pH}_{SQ/QH_2} = \Delta G^{0}_{SQ/QH_2} - 2RT\ln([H^+]) \quad \text{Eq. S5}$$

For the semiquinone/quinol couple at the Qp-site, the proton concentration is taken with respect to the positive side of the membrane ($[H^+]_p$). For the Qn-site, the proton concentration is taken with respect to the negative side of the membrane ($[H^+]_n$).

In a similar manner, the quinone/quinol couple free energy is pH dependent and calculated using Eq. S6.

$$\Delta G^{pH}_{Q/QH_2} = \Delta G^{0}_{Q/QH_2} - RT\ln([H^+]) \quad \text{Eq. S6}$$

The free energy of electron transfer for the b hemes are also pH-dependent but possess pKs close to neutral pH. These free energies are computed using Eqs. S7 and S8.

$$\Delta G^{pH}_{b_L^{ox}/b_L^{red}} = \Delta G^{0}_{b_L^{ox}/b_L^{red}} + RT\ln\left(([H^+]+10^{-pK_{bLox}})/([H^+]+10^{-pK_{bLred}})\right) \quad \text{Eq. S7}$$

$$\Delta G^{pH}_{b_H^{ox}/b_H^{red}} = \Delta G^{0}_{b_H^{ox}/b_H^{red}} + RT\ln\left(([H^+]+10^{-pK_{bHox}})/([H^+]+10^{-pK_{bHred}})\right) \quad \text{Eq. S8}$$

For the heme b_L, the proton concentration is taken with respect to the positive side of the membrane. And for heme b_H, the proton concentration is taken with respect to the negative side of the membrane.

Semiquinone stability constants and free energies. To calculate the semiquinone free energies from the semiquinone stability constant and ubiquinone/ubiquinol couple free energy, the following relations were used:

$$\Delta G^{pH}_{Q/QH_2} = \Delta G_{Q/SQ} + \Delta G^{pH}_{SQ/QH_2} \quad \text{Eq. S9}$$

$$\Delta G_{Q/SQ} - \Delta G^{pH}_{SQ/QH_2} = -RT\ln(K_S) \quad \text{Eq. S10}$$

Equilibrium constants. The equilibrium constants for electron transfer for quinol oxidation at the Qp-site, quinone reduction at the Qn-site, and superoxide production at the Qp-site are given below.

$$K^{eq}_{Qp} = e^{-(\Delta_{et}G_{c^{3+}/c^{2+}} - \Delta_{et}G^{Qp}_{SQ/QH_2})/RT} \quad \text{Eq. S11}$$

$$K^{eq}_{Qn} = e^{-(\Delta_{et}G^{Qp}_{SQ/QH_2} - \Delta G^{pH}_{b_H^{ox}/b_H^{red}})/RT} \quad \text{Eq. S12}$$

$$K^{eq}_{SO} = e^{-(\Delta G^{0}_{O_2/O_2^{\cdot -}} - \Delta_{et}G^{Qp}_{SQ/QH_2})/RT} \quad \text{Eq. S13}$$

Substate distribution. For each state (oxidized to five-electron reduced), the complex exists in a combination of redox states that depends on the total number of reachable redox centers and the number of electrons on the complex. To account for Coulombic repulsion forces, we include neighbor interactions for intra-monomer b_L/b_H and inter-monomer b_L/b_L pairs. For details, see below.

monomer a

$$\Delta G^{a}_{Q/SQn} = \Delta_{et} G_{Q/SQn} - RT\ln\left([Q]/K^{Qn}_{Q}/P_{Qn}\right) \qquad \text{Eq. S14}$$

If an antimycin A molecule is bound to the Qn-site of monomer a, Eq. S14 becomes

$$\Delta G^{a}_{Q/SQn} = \infty \qquad \text{Eq. S15}$$

$$\Delta G^{a}_{b^{ox}_{H}/b^{red}_{H}} = \Delta G^{pH}_{b^{ox}_{H}/b^{red}_{H}} + 2F\beta\Delta\Psi \qquad \text{Eq. S16}$$

$$\Delta G^{a}_{b^{ox}_{L}/b^{red}_{L}} = \Delta G^{pH}_{b^{ox}_{L}/b^{red}_{L}} \qquad \text{Eq. S17}$$

$$\Delta G^{a}_{Q/SQp} = \Delta_{et} G_{Q/SQp} - RT\ln\left([Q]/K^{Qp}_{Q}/P_{Qp}\right) \qquad \text{Eq. S18}$$

monomer b

$$\Delta G^{b}_{Q/SQn} = \Delta_{et} G_{Q/SQn} - RT\ln\left([Q]/K^{Qn}_{Q}/P_{Qn}\right) \qquad \text{Eq. S19}$$

If an antimycin A molecule is bound to the Qn-site of monomer b, Eq. S19 becomes

$$\Delta G^{b}_{Q/SQn} = \infty \qquad \text{Eq. S20}$$

$$\Delta G^{b}_{b^{ox}_{H}/b^{red}_{H}} = \Delta G^{pH}_{b^{ox}_{H}/b^{red}_{H}} + 2F\beta\Delta\Psi \qquad \text{Eq. S21}$$

$$\Delta G^{b}_{b^{ox}_{L}/b^{red}_{L}} = \Delta G^{pH}_{b^{ox}_{L}/b^{red}_{L}} \qquad \text{Eq. S22}$$

$$\Delta G^{b}_{Q/SQp} = \infty \qquad \text{Eq. S23}$$

If a single antimycin A molecule is bound to the dimer, then Eq. S23 becomes

$$\Delta G^{b}_{Q/SQp} = \Delta_{et} G_{Q/SQp} - RT\ln\left([Q]/K^{Qp}_{Q}/P_{Qp}\right) \qquad \text{Eq. S24}$$

For the Coulombic electrostatic interactions, energy penalties were added to the redox state combinations containing intra-monomer b_L/b_H and inter-monomer b_L/b_L pairs. These energies are given in Table 1. However, in order to satisfy detailed

balance, this penalty was not applied to redox centers including intra-monomer b_L-b_H-SQn combinations for three-electron reduced states and higher.

To calculate the substate distribution for each enzyme state, we need all the possible combinations of redox centers. To do this, we use combinatorics. For example, the two-electron reduced dimer has a total of 8!/2!/(8-2)! = 28 substate combinations, where 8 is the total number of possible redox centers and 2 is the number of electrons on the dimer. To calculate the standard free energy change for each redox center combination, we assume linear combinations of the individual standard free energy change for each redox center described by Eqs. S14-S24. We then use the Boltzmann distribution to calculate the reduced fraction of each redox center combination on the dimer with a given number of electrons.

State occupancy solution. For the state occupancies, Eq. S25 was solved. For brevity, only the matrix equation is shown. The analytical solution consists of several thousand terms. Electrons are added or removed from the complex either one at a time at the Qp-site or two at a time at the Qn-site. The last row in Eq. S25 is a conservation equation to fix the sum of the state occupancies to equal one.

$$\begin{bmatrix} -(k_{01}+k_{02}) & k_{10} & k_{20} & 0 & 0 & 0 \\ k_{01} & -(k_{10}+k_{12}+k_{13}) & k_{21} & k_{31} & 0 & 0 \\ k_{02} & k_{12} & -(k_{20}+k_{21}+k_{23}) & k_{32} & k_{42} & 0 \\ 0 & k_{13} & k_{23} & -(k_{31}+k_{32}+k_{34}) & k_{43} & k_{53} \\ 0 & 0 & k_{24} & k_{34} & -(k_{42}+k_{43}+k_{45}) & k_{54} \\ 0 & 0 & 0 & k_{35} & k_{45} & -(k_{53}+k_{54}) \\ 1 & 1 & 1 & 1 & 1 & 1 \end{bmatrix} \begin{bmatrix} E_0 \\ E_1 \\ E_2 \\ E_3 \\ E_4 \\ E_5 \end{bmatrix} = \begin{bmatrix} 0 \\ 0 \\ 0 \\ 0 \\ 0 \\ 1 \end{bmatrix} \quad \text{Eq. S25}$$

State transition rate constants. The state transition rate constants, $k_{i,j}$, are the summation of the partial one-step and two-step oxidation/reduction reactions. They include both the forward and reverse reactions that facilitate transitions from state *i* to state *j*. For completeness, they are given below in Eqs. S26-S43. These equations are for the antimycin A free turnover conditions. Therefore, only one Qp-site is active. When antimycin A is bound to a single monomer, both are active (23). Both Qn-sites are available for turnover except when antimycin A is bound. The superscript *a* and *b* designates which monomer in the dimer is involved in the partial reaction. The equations for the binding terms B_f, B_r, and B_Q are given in Eqns. S48-S54.

$$k_{01} = kf_{Q_p}^{0,1} B_f + B_Q [O_2^{-}] kr_{SO} \quad \text{Eq. S26}$$

$$k_{10} = \left(kr_{Q_p}^{0,1} B_r + [O_2] kf_{SO} \right) s^1_{r \in \left\{ SQ_p^a \right\}} \quad \text{Eq. S27}$$

$$k_{12} = \left(kf_{Q_p}^{1,2} B_f + B_Q [O_2^{-}] kr_{SO} \right) \sum_{\forall r \notin \left\{ SQ_p^a \right\}} s_r^1 \quad \text{Eq. S28}$$

$$k_{21} = \left(kr_{Q_p}^{1,2} B_r + [O_2] kf_{SO} \right) \sum_{\forall r \in \left\{ SQ_p^a \right\}} s_r^2 \quad \text{Eq. S29}$$

$$k_{20} = e^{-2\beta\Delta\Psi/2RT} kf_{Q_n}^{2,0} \left(s^2_{r\in\left\{b_L^{red,a}\cdot SQ_n^a\right\}} + s^2_{r\in\left\{b_L^{red,b}\cdot SQ_n^b\right\}} \right)$$ Eq. S30

$$k_{02} = 2e^{2\beta\Delta\Psi/2RT} kr_{Q_n}^{2,0}$$ Eq. S31

$$k_{23} = \left(kf_{Q_p}^{2,3} B_f + B_Q [O_2^-] kr_{SO} \right) \sum_{\forall r\notin\left\{SQ_p^a\right\}} s_r^2$$ Eq. S32

$$k_{32} = \left(kr_{Q_p}^{2,3} B_r + [O_2] kf_{SO} \right) \sum_{\forall r\in\left\{SQ_p^a\right\}} s_r^3$$ Eq. S33

$$k_{31} = e^{-2\beta\Delta\Psi/2RT} kf_{Q_n}^{3,1} \left(s^3_{r\in\left\{b_L^{red,a}\cdot SQ_n^a\right\}} + s^3_{r\in\left\{b_L^{red,b}\cdot SQ_n^b\right\}} \right)$$ Eq. S34

$$k_{13} = e^{2\beta\Delta\Psi/2RT} kr_{Q_n}^{3,1} \left(\sum_{r\notin\left\{b_L^{red,a}, SQ_n^a\right\}} s_r^1 + \sum_{r\notin\left\{b_L^{red,b}, SQ_n^b\right\}} s_r^1 \right)$$ Eq. S35

$$k_{34} = \left(kf_{Q_p}^{3,4} B_f + B_Q [O_2^-] kr_{SO} \right) \sum_{\forall r\notin\left\{SQ_p^a\right\}} s_r^3$$ Eq. S36

$$k_{43} = \left(kr_{Q_p}^{3,4} B_r + [O_2] kf_{SO} \right) \sum_{\forall r\in\left\{SQ_p^a\right\}} s_r^4$$ Eq. S37

$$k_{42} = e^{-2\beta\Delta\Psi/2RT} kf_{Q_n}^{4,2} \left(s^4_{r\in\left\{b_L^{red,a}\cdot SQ_n^a\right\}} + s^4_{r\in\left\{b_L^{red,b}\cdot SQ_n^b\right\}} \right)$$ Eq. S38

$$k_{24} = e^{2\beta\Delta\Psi/2RT} kr_{Q_n}^{4,2} \left(\sum_{r\notin\left\{b_L^{red,a}\cdot SQ_n^a\right\}} s_r^2 + \sum_{r\notin\left\{b_L^{red,b}\cdot SQ_n^b\right\}} s_r^2 \right)$$ Eq. S39

$$k_{45} = \left(kf_{Q_p}^{4,5} B_f + B_Q [O_2^-] kr_{SO} \right) \sum_{\forall r\notin\left\{SQ_p^a\right\}} s_r^4$$ Eq. S40

$$k_{54} = \left(kr_{Q_p}^{4,5} B_r + [O_2] kf_{SO}\right) \sum_{\forall r \in \{SQ_p^a\}} s_r^5 \quad \text{Eq. S41}$$

$$k_{53} = e^{-2\beta\Delta\Psi/2RT} kf_{Q_n}^{5,3} \left(s^5_{r \in \{b_L^{red,a} \cdot SQ_n^a\}} + s^5_{r \in \{b_L^{red,b} \cdot SQ_n^b\}} \right) \quad \text{Eq. S42}$$

$$k_{35} = e^{2\beta\Delta\Psi/2RT} kr_{Q_n}^{3,5} \left(\sum_{r \notin \{b_L^{red,a} \cdot SQ_n^a\}} s_r^3 + \sum_{r \notin \{b_L^{red,b} \cdot SQ_n^b\}} s_r^3 \right) \quad \text{Eq. S43}$$

Steady State Equations. State transitions occur from the appropriate substate fraction with rate constants determined by model fitting. Net turnover at each site is computed using the following equations.

$$J_c = B_f \sum_i k_{i,i+1} E_i \sum_{\forall r \notin \{SQ_p\}} s_r^i - B_r \sum_i k_{i+1,i} E_{i+1} \sum_{\forall r \in \{SQ_p\}} s_r^{i+1}, \quad \text{Eq. S44}$$

$$J_{SO} = [O_2] kf_{SO} \sum_i E_i \sum_{\forall r \in \{SQ_p\}} s_r^i - B_Q [O_2^{\cdot -}] kr_{SO} \sum_i E_{i+1} \sum_{\forall r \notin \{SQ_p\}} s_r^{i+1}, \quad \text{Eq. S45}$$

$$J_{Qp} = J_c, \quad \text{Eq. S46}$$

and

$$J_{Qn} = \left(J_c - J_{SO}\right)/2 \quad \text{Eq. S47}$$

where i is for the ith electronic state given by solving Eq. S25. State transitions simulated by the model are represented in Fig. 1B. The net cytochrome c reduction (J_c) and superoxide production (J_{SO}) rates are given by Eqns. S44 and S45, respectively. Quinol oxidation at the Qp-site (J_{Qp}) and quinone reduction at the Qn-site (J_{Qn}) are stoichiometrically linked to the cytochrome c reduction and superoxide production rates as shown in Eqs. S46 and S47, respectively. Only one Qp-site is active on the dimer unless a single antimycin A molecule is bound to the dimer (23). Substrate, product, and proton bound complexes are computed using B_f, B_r, and B_Q as shown in Eqs. S48-S54. Proton concentration for the ISP protonation state given in B_f is taken with respect to the positive side of the membrane. For these equations, we assume substrate, product, and proton bound and unbound states are in rapid equilibrium. In addition, we assume the c^{2+} and c^{3+} compete with each other for the c_1-site, and QH_2 and Q compete with each other for the Qp-site and Qn-site, respectively. And for state transitions to occur, the enzyme needs to be in the proper enzyme-substrate complex (e.g., for Qp-site catalysis, the enzyme needs to be bound with QH_2 and c^{3+}; recall that we lump QH_2 oxidation and c^{3+} reduction into a single step for simplicity).

$$B_f = [QH_2] / K_{QH2}^{Qp} / P_{Qp} \cdot [c^{3+}] / K_{c3+} / P_c \cdot [H^+] / K_B^{ISPox} / P_{ISP} \quad \text{Eq. S48}$$

$$B_r = [c^{2+}]/K_{c2+}/P_c \cdot [H^+]/K_B^{ISPox}/P_{ISP}$$ Eq. S49

$$B_Q = [Q]/K_Q^{Qp}/P_{Qp}$$ Eq. S50

$$P_{ISP} = \left(1+[H^+]/K_A^{ISPox}\right)\left(1+[H^+]/K_B^{ISPox}\right)$$ Eq. S51

$$P_{Qp} = 1+[QH_2]/K_{QH2}^{Qp}+[Q]/K_Q^{Qp}$$ Eq. S52

$$P_{Qn} = 1+[QH_2]/K_{QH2}^{Qn}+[Q]/K_Q^{Qn}$$ Eq. S53

$$P_c = 1+[c^{3+}]/K_{c3+}+[c^{2+}]/K_{c2+}$$ Eq. S54

Parameter sensitivity matrix and correlation coefficients. The normalized parameter sensitivity matrix is computed using Eq. S55. Each model output, f_i, is congruent with the experimental data. The parameter sensitivities were computed using a complex variable approach (25).

$$S_{i,j} = \frac{\partial f_i}{\partial p_j}\frac{p_j}{f_i}$$ Eq. S55

The sensitivity coefficients presented in Table 1 were computed by averaging all the non-zero sensitivity coefficient for a given parameter. This was done by using Eq. S56.

$$\overline{s_j} = \frac{1}{N_j}\sum_{\forall i:S_{i,j}\neq 0} S_{i,j}$$ Eq. S56

where N_j is the number of non-zero elements in jth column of S. The parameter correlation coefficients are pairwise linear correlation coefficients computed for each pair of columns in the normalized parameter sensitivity matrix.

Antimycin inhibited state transition rate constants. When antimycin A is bound to a given monomer, we assume the rate of QH_2 oxidation at the Qp-site is slowed. This is discussed in more detail in the main text. The inhibition factors, β_{AA}^r and β_{AA}^f, are given in Table 1. The antimycin A binding constant, K_{AA}, is 30 pM (26).

$$k_{fQp}^{0,1} = k_{fQp}^{0,1}\left(\frac{1+[AA]/K_{AA}}{1+\beta_{AA}^{r}[AA]/K_{AA}}\right) \quad \text{Eq. S57}$$

$$k_{fQp}^{1,2} = k_{fQp}^{1,2}\left(\frac{1+[AA]/K_{AA}}{1+\beta_{AA}^{r}[AA]/K_{AA}}\right) \quad \text{Eq. S58}$$

$$k_{fQp}^{2,3} = k_{fQp}^{2,3}\left(\frac{1+[AA]/K_{AA}}{1+\beta_{AA}^{r}[AA]/K_{AA}}\right) \quad \text{Eq. S59}$$

$$k_{fQp}^{3,4} = k_{fQp}^{3,4}\left(\frac{1+[AA]/K_{AA}}{1+\beta_{AA}^{r}[AA]/K_{AA}}\right) \quad \text{Eq. S60}$$

$$k_{fQp}^{4,5} = k_{fQp}^{4,5}\left(\frac{1+[AA]/K_{AA}}{1+\beta_{AA}^{f}[AA]/K_{AA}}\right) \quad \text{Eq. S61}$$

Kinetic fits. Model simulations of the kinetic data is shown below. When no uncertainty was given, we assumed an error model of 20% the maximum for a given data set.

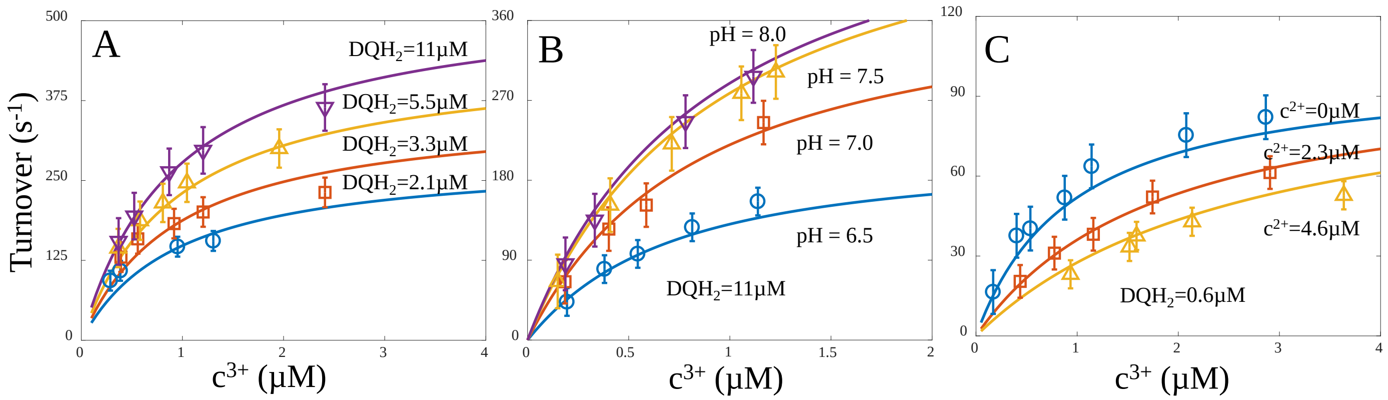

Figure S5. The turnover rate of purified bc_1 complex oxidizing the Q-analogue decylubiquinol (DQH_2). (A) The simulated rate of cyt c reduction versus c^{3+} concentration at various fixed DQH_2 concentrations. (B) The simulated rate of cyt c reduction versus c^{3+} concentration at various fixed pH at a fixed DQH2 concentration. (C) The simulated rate of cyt c reduction versus c^{3+} concentration at various fixed c^{2+} concentrations at a fixed DQH_2 concentration. The symbols represent the experimental data and lines represent model simulations. Data from (17).

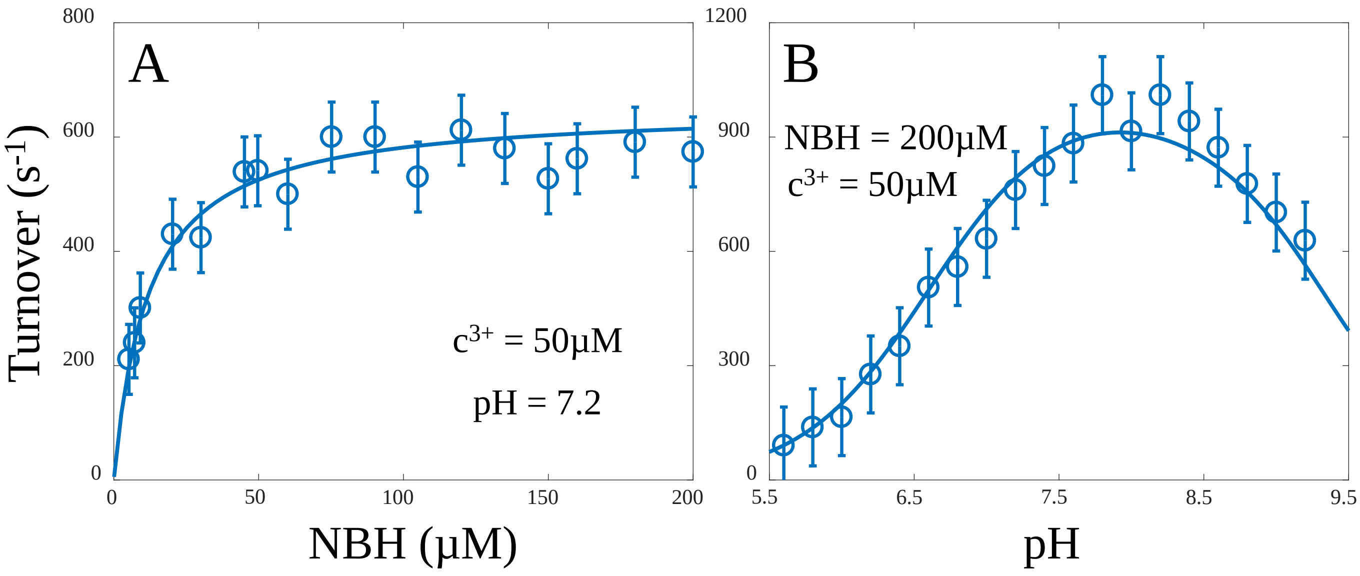

Figure S6. The turnover rate of reconstituted bc_1 complex in proteoliposomes oxidizing the Q-analogue nonylubiquinol (NBH). (A) The simulated turnover rate versus NBH concentration at fixed pH and cytochrome c^{3+} concentration. (B) The simulated turnover rate versus pH at fixed cytochrome c^{3+} and NBH concentrations. Data from (1).

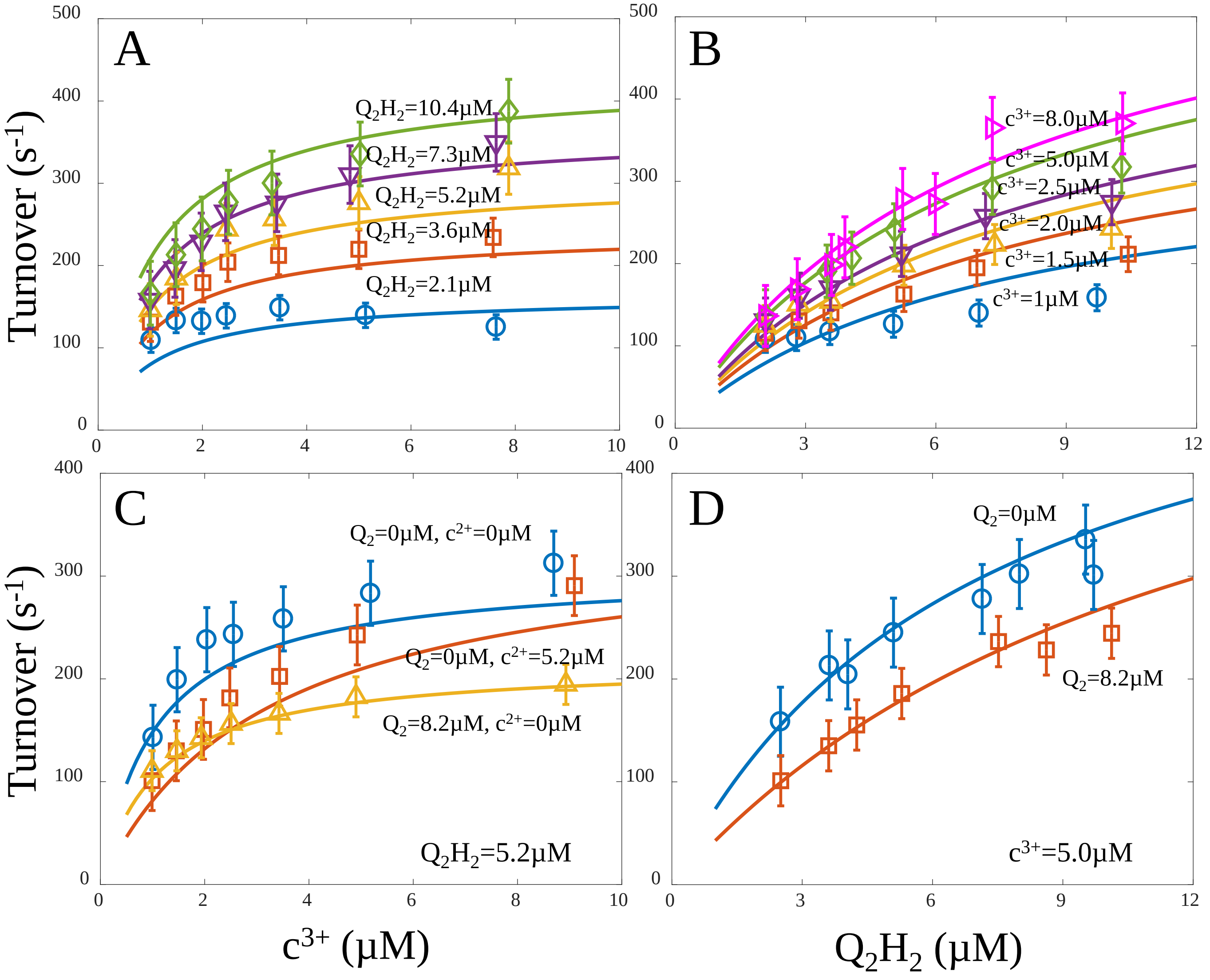

Figure S7. The turnover rate of purified bc_1 complex oxidizing the Q-analogue ubiquinol-2 (Q_2H_2). (A) The simulated turnover rate versus cytochrome c^{3+} concentration at varying Q_2H_2 concentrations. (B) The simulated turnover rate versus Q_2H_2 concentration at varying cytochrome c^{3+} concentrations. (C) The simulated turnover rate versus cytochrome c^{3+} concentration at varying Q_2 and cytochrome c^{2+} concentrations at a fixed Q_2H_2 concentration. (D) The simulated turnover rate versus Q_2H_2 concentration at varying Q_2 concentrations at a fixed cytochrome c^{3+} concentration. Fixed concentrations for each simulation are indicated on each panel. The symbols represent the experimental data and lines represent model simulations. Data from (18).

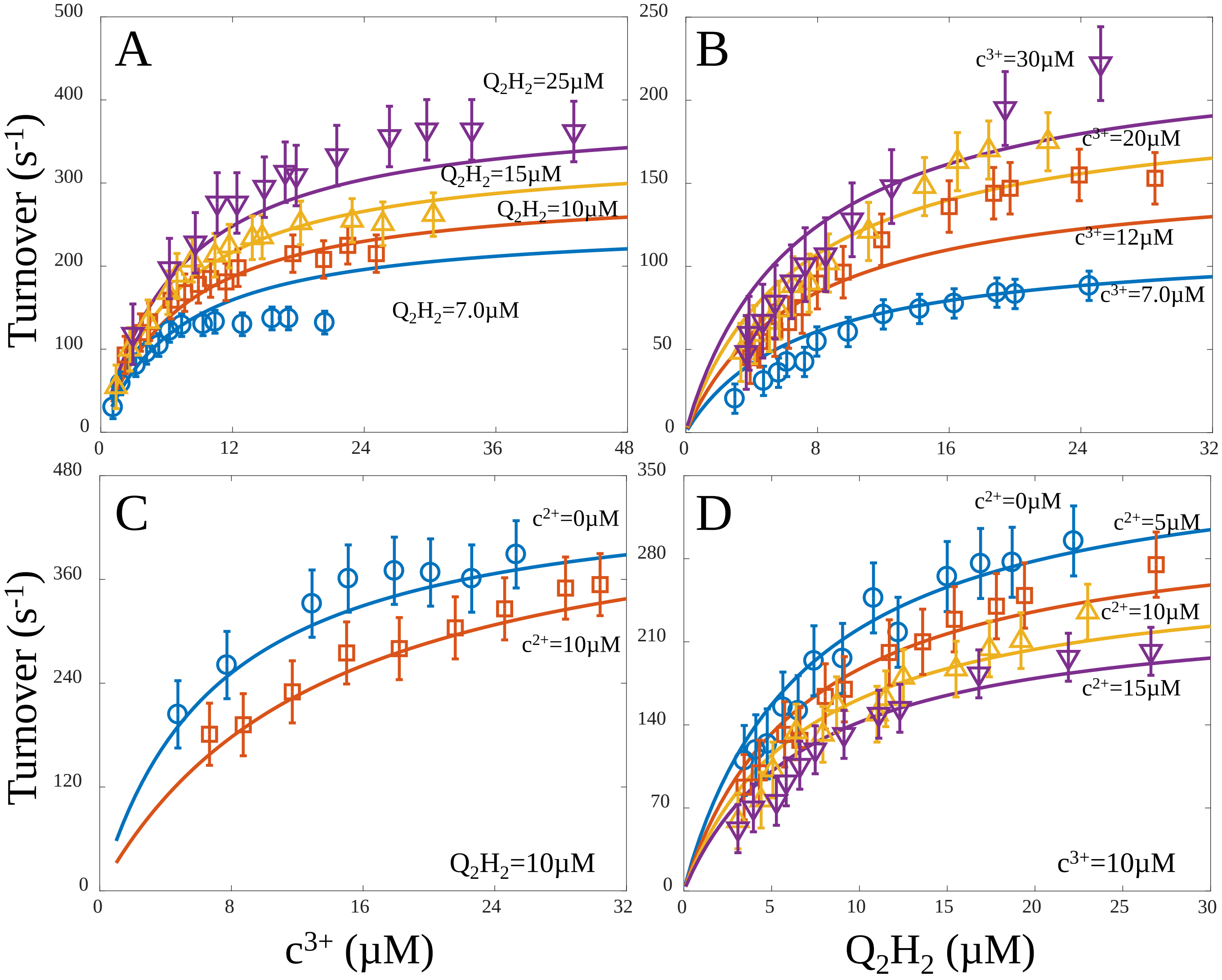

Figure S8. The turnover rate of purified bc_1 complex oxidizing the Q-analogue ubiquinol-2 (Q_2H_2). (A) The simulated turnover rate versus cytochrome c^{3+} concentration at various fixed Q_2H_2 concentrations. (B) The simulated turnover rate versus Q_2H_2 concentration at various fixed cytochrome c^{3+} concentrations. (C) The simulated turnover rate versus cytochrome c^{3+} concentration at various fixed cytochrome c^{2+} concentrations at a fixed Q_2H_2 concentration. (D) The simulated turnover rate versus Q_2H_2 concentration at various fixed cytochrome c^{2+} concentrations at a fixed cytochrome c^{3+} concentration. The symbols represent the experimental data and lines represent model simulations. Data from (19).

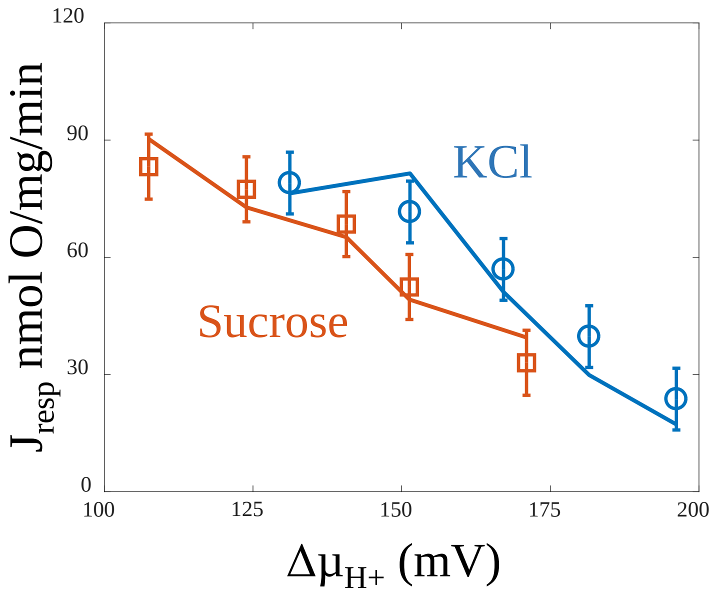

Figure S9. The effect of a proton-motive force on turnover. (A) The steady-state rate of oxygen consumption by isolated rat liver mitochondria in the presence of rotenone and oligomycin versus the proton-motive force is shown. The oxygen consumption rate is equivalent to two times the turnover rate of the bc_1 complex. The blue line and circles reflect the simulated turnover rate and data, respectively, when the mitochondria are suspended in a KCl-based respiration buffer. The orange line and circles show the simulated turnover rate and data, respectively, when the respiration buffer is switched to sucrose-based buffer. Exogenous Q_2H_2 and cytochrome c were supplied to the mitochondrial suspension. The proton-motive force is defined as: $\Delta\mu_{H+} = \Delta\Psi + 59\Delta pH$. Data from (20).

Model Code Description

During the editorial process, each file in the zip was appended with "_V1." In order to run the code without issue, first remove "_V1" from each file.

bc1_code.zip – zip file containing the following files:

> bc1_data.mat – mat file containing data structure
>
> bc1_params.mat – mat file containing model parameters
>
> bc1_dimer.m – function containing dimer model code
>
> Generate_Figures.m – function containing code to simulate the model and plot the figures

Supporting References

1. Brandt, U., and J. G. Okun. 1997. Role of deprotonation events in ubihydroquinone:cytochrome c oxidoreductase from bovine heart and yeast mitochondria. Biochemistry 36:11234-11240.
2. Rich, P. R., A. E. Jeal, S. A. Madgwick, and A. J. Moody. 1990. Inhibitor effects on redox-linked protonations of the b haems of the mitochondrial bc1 complex. Biochimica et biophysica acta 1018:29-40.
3. Eddowes, M. J., and H. A. O. Hill. 1979. Electrochemistry of Horse Heart Cytochrome-C. J Am Chem Soc 101:4461-4464.
4. Crofts, A. R., V. P. Shinkarev, D. R. Kolling, and S. Hong. 2003. The modified Q-cycle explains the apparent mismatch between the kinetics of reduction of cytochromes c1 and bH in the bc1 complex. J Biol Chem 278:36191-36201.
5. Urban, P. F., and M. Klingenberg. 1969. On the redox potentials of ubiquinone and cytochrome b in the respiratory chain. Eur J Biochem 9:519-525.
6. Ohnishi, T., and B. L. Trumpower. 1980. Differential effects of antimycin on ubisemiquinone bound in different environments in isolated succinate . cytochrome c reductase complex. J Biol Chem 255:3278-3284.
7. Brandt, U. 2006. Energy converting NADH:quinone oxidoreductase (complex I). Annu Rev Biochem 75:69-92.
8. Glaser, E. G., S. W. Meinhardt, and A. R. Crofts. 1984. Reduction of cytochrome b-561 through the antimycin-sensitive site of the ubiquinol-cytochrome c2 oxidoreductase complex of Rhodopseudomonas sphaeroides. FEBS letters 178:336-342.
9. Glaser, E. G., and A. R. Crofts. 1984. A new electrogenic step in the ubiquinol:cytochrome c2 oxidoreductase complex of Rhodopseudomonas sphaeroides. Biochimica et biophysica acta 766:322-333.
10. Robertson, D. E., and P. L. Dutton. 1988. The nature and magnitude of the charge-separation reactions of ubiquinol cytochrome c2 oxidoreductase. Biochimica et biophysica acta 935:273-291.
11. Ding, H., C. C. Moser, D. E. Robertson, M. K. Tokito, F. Daldal, and P. L. Dutton. 1995. Ubiquinone pair in the Qo site central to the primary energy conversion reactions of cytochrome bc1 complex. Biochemistry 34:15979-15996.
12. Wikström, M., and Royal Society of Chemistry (Great Britain). 2005. Biophysical and structural aspects of bioenergetics. Royal Society of Chemistry, Cambridge, UK.
13. Lass, A., S. Agarwal, and R. S. Sohal. 1997. Mitochondrial ubiquinone homologues, superoxide radical generation, and longevity in different mammalian species. J Biol Chem 272:19199-19204.
14. Schwerzmann, K., L. M. Cruz-Orive, R. Eggman, A. Sanger, and E. R. Weibel. 1986. Molecular architecture of the inner membrane of mitochondria from rat liver: a combined biochemical and stereological study. J Cell Biol 102:97-103.
15. Murphy, M. P. 2009. How mitochondria produce reactive oxygen species. The Biochemical journal 417:1-13.
16. Kuo-chen, C., and J. Shou-ping. 1974. Studies on the rate of diffusion-controlled reactions of enzymes. Spatial factor and force field factor. Sci Sin 27:664-680.
17. Speck, S. H., and E. Margoliash. 1984. Characterization of the interaction of cytochrome c and mitochondrial ubiquinol-cytochrome c reductase. J Biol Chem 259:1064-1072.
18. Esposti, M. D., and G. Lenaz. 1991. The kinetic mechanism of ubiquinol: cytochrome c reductase at steady state. Archives of biochemistry and biophysics 289:303-312.
19. Kubota, T., S. Yoshikawa, and H. Matsubara. 1992. Kinetic mechanism of beef heart ubiquinol:cytochrome c oxidoreductase. Journal of biochemistry 111:91-98.
20. Brown, G. C., and M. D. Brand. 1985. Thermodynamic control of electron flux through mitochondrial cytochrome bc1 complex. The Biochemical journal 225:399-405.
21. Rottenberg, H., R. Covian, and B. L. Trumpower. 2009. Membrane potential greatly enhances superoxide generation by the cytochrome bc1 complex reconstituted into phospholipid vesicles. J Biol Chem 284:19203-19210.
22. Quinlan, C. L., A. A. Gerencser, J. R. Treberg, and M. D. Brand. 2011. The mechanism of superoxide production by the antimycin-inhibited mitochondrial Q-cycle. J Biol Chem 286:31361-31372.
23. Covian, R., E. B. Gutierrez-Cirlos, and B. L. Trumpower. 2004. Anti-cooperative oxidation of ubiquinol by the yeast cytochrome bc1 complex. J Biol Chem 279:15040-15049.

24. Drose, S., and U. Brandt. 2008. The mechanism of mitochondrial superoxide production by the cytochrome bc1 complex. J Biol Chem 283:21649-21654.
25. Squire, W., and G. Trapp. 1998. Using complex variables to estimate derivatives of real functions. Siam Rev 40:110-112.
26. Castellani, M., R. Covian, T. Kleinschroth, O. Anderka, B. Ludwig, and B. L. Trumpower. 2010. Direct demonstration of half-of-the-sites reactivity in the dimeric cytochrome bc1 complex: enzyme with one inactive monomer is fully active but unable to activate the second ubiquinol oxidation site in response to ligand binding at the ubiquinone reduction site. J Biol Chem 285:502-510.
